# Supplementary material for: TSG101 associates with PARP1 and is essential for PARylation and DNA damage‐induced NF‐κB activation
Source: EMBO J. 2022 Sep 20;41(21):e110372. doi: 10.15252/embj.2021110372 (PMC9627669; doi:10.15252/embj.2021110372)
Supplement: Supplementary file 1 — Expanded View Figures PDF [file EMBJ-41-e110372-s010.pdf]

Expanded View Figures

Figure EV1. Genome-wide siRNA screening and hit selection.

A HEK-Luc(tGFP) NF-κB reporter cells were treated with the indicated conditions (50 μM etoposide, 20 Gy irradiation, or 10 ng/ml TNFα). As the NF-κB-driven expression and translation of turbo GFP require more time than the initial activation of the NF-κB pathway, the treatment conditions were alternatively prolonged to 4.5 h. Whole-cell extracts were obtained and immunoblotted with the indicated antibodies.

B Detailed layout of the genome-wide siRNA screening, carried out using a one-plate-to-one target approach. The indicated controls were added to the siRNA library. As another control, cells were treated with the vehicle DMSO (Column 24). The heatmap displays the Z-scores of tested candidates and controls of a representative plate taken from the siRNA library screen.

C Spearman's rank correlation coefficient test of representative screening plate replicates demonstrated high assay reproducibility. Same screening plates and layouts were tested, and results were compared with each other (R1 vs R2).

D TSG101 cluster was visualized using STRING database (Szklarczyk *et al*, 2019). Arrows or lines between candidate hits represent protein–protein interactions observed in previous publications.

E Representative high-confidence DNA damage-selective hits are shown for etoposide (left panel) and TNFα (right panel) screens. The conditions were compared with an ordinary one-way ANOVA (ns, $P > 0.05$; ****$P < 0.0001$). Results were obtained from three biologically independent experiments. Error bars represent mean ± SD.

© 2022 The Authors

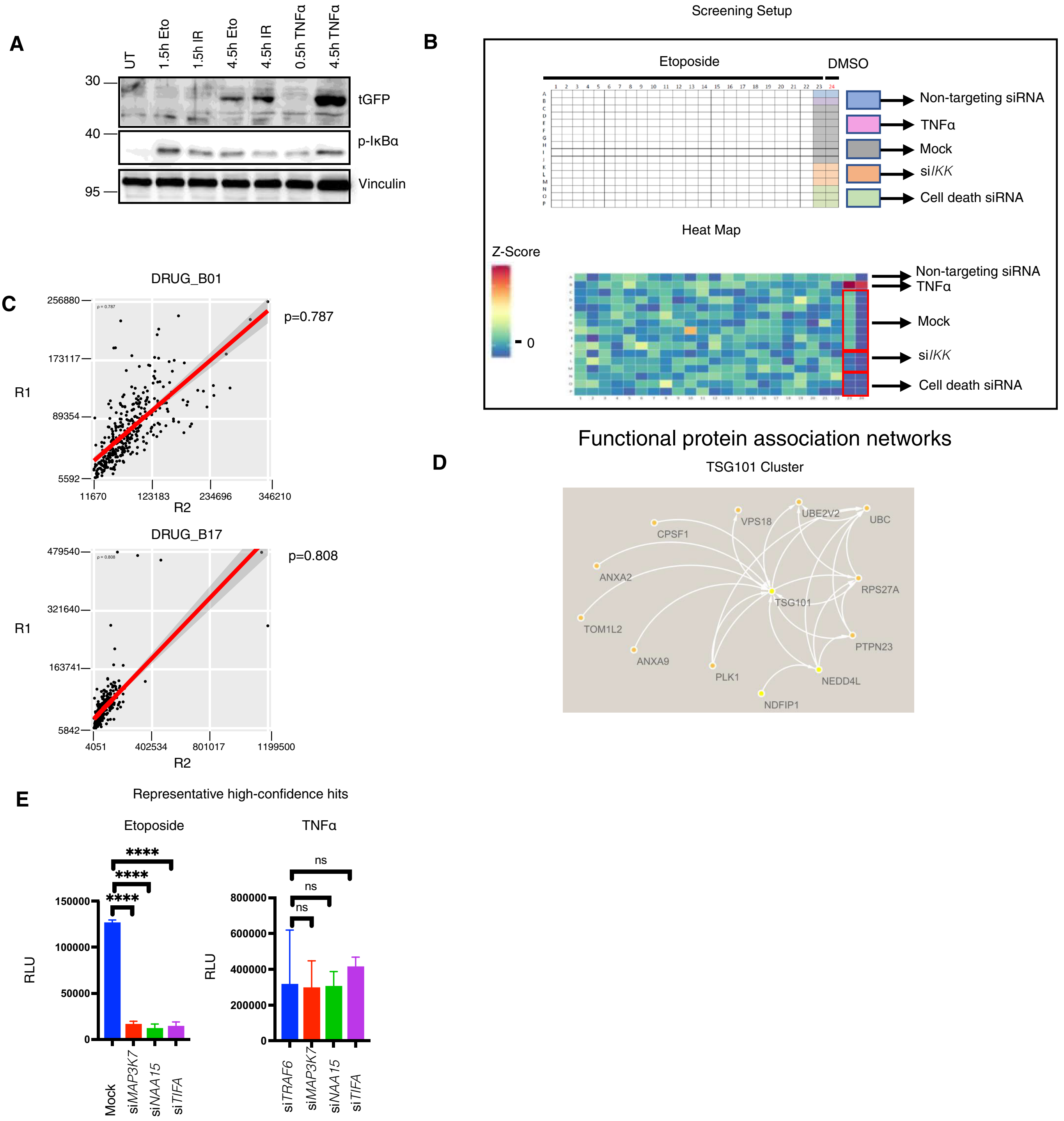
A
UT
1.5h Eto
1.5h IR
4.5h Eto
4.5h IR
0.5h TNFα
4.5h TNFα
30
40
95
tGFP
p-IκBα
Vinculin
B
Screening Setup
Etoposide
DMSO
Non-targeting siRNA
TNFα
Mock
siIKK
Cell death siRNA
Heat Map
Z-Score
0
Non-targeting siRNA
TNFα
Mock
siIKK
Cell death siRNA
C
DRUG_B01
p=0.787
R1
R2
256880
173117
89354
5592
11670
123183
234696
346210
DRUG_B17
p=0.808
479540
321640
163741
5842
4051
402534
801017
1199500
D
Functional protein association networks
TSG101 Cluster
VPS18
UBE2V2
UBC
CPSF1
ANXA2
RPS27A
TSG101
TOM1L2
PTPN23
ANXA9
PLK1
NEDD4L
NDFIP1
E
Representative high-confidence hits
Etoposide
TNFα
RLU
****
****
****
150000
100000
50000
0
Mock
siMAP3K7
siNAA15
siTIFA
ns
ns
ns
800000
600000
400000
200000
0
siTRAF6
siMAP3K7
siNAA15
siTIFA

Figure EV1.

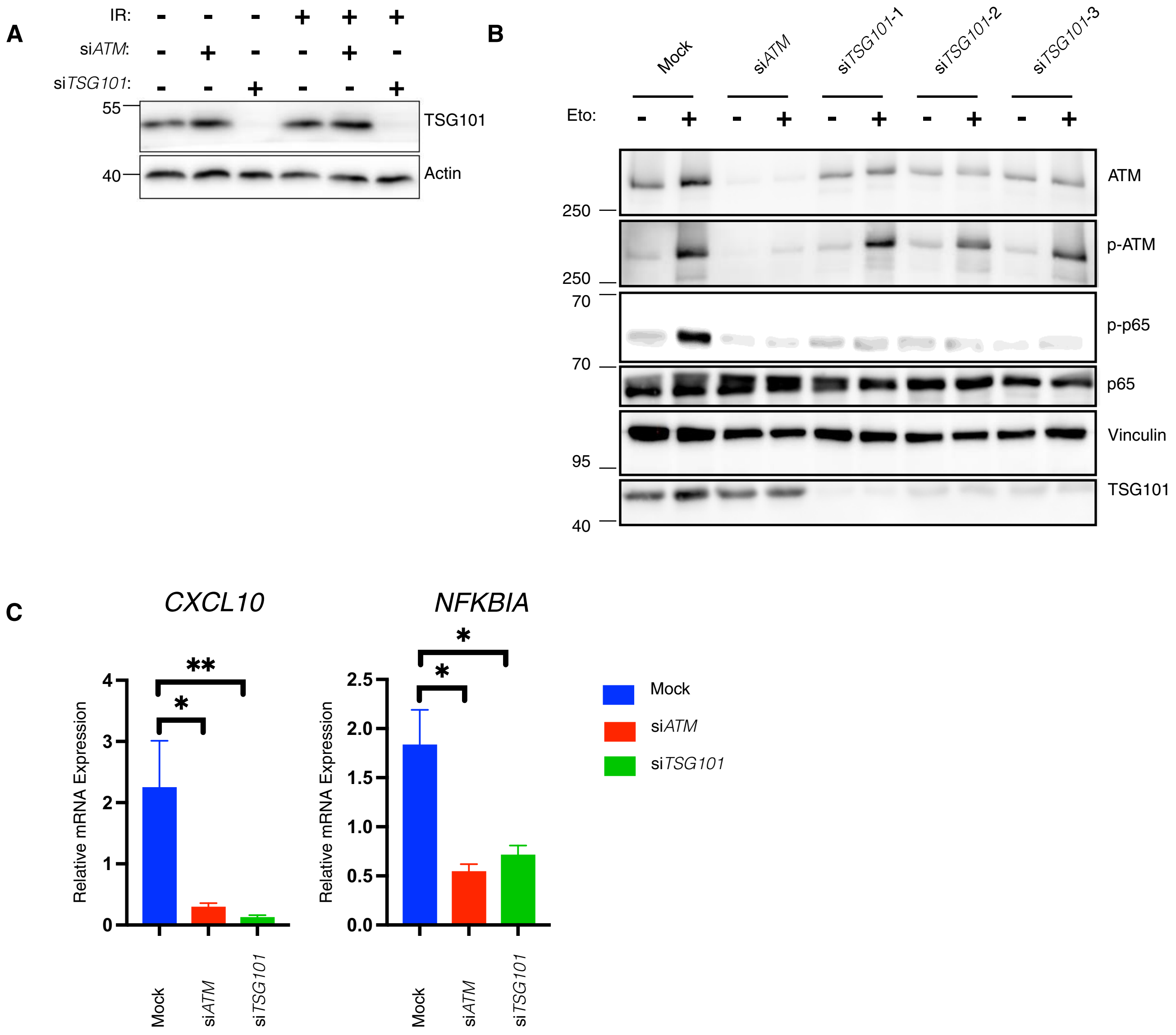

Figure EV2. TSG101 and ATM are equivalently essential for NF-κB activation by DNA damage.

A HEK-Luc(tGFP) NF-κB reporter cells were transfected with the same transfection mixtures as used in Fig 2A. Whole-cell extracts were immunoblotted with antibodies against the indicated proteins. Actin was used as a loading control.

B U2-OS cells were transfected with nontargeting (mock) or *ATM*-directed siRNAs or three different *TSG101*-targeting siRNAs. The indicated cells were treated with etoposide (50 µM, 90 min. Before analysis). Whole-cell extracts were immunoblotted with the indicated antibodies.

C U2-OS cells were transfected with the indicated siRNAs. The NF-κB pathway was activated by etoposide treatment (50 µM, 90 min before analysis). Expression of indicated genes was analyzed using qRT–PCR. The mRNA expression of these genes was normalized to the expression of three housekeeping genes, *ACTA1*, *RPL13A*, and *TBP2*. The gene expression for the indicated conditions is relative to the nontargeting siRNA-transfected vehicle (DMSO)-treated cells. The result is representative of three biologically independent experiments. The conditions were compared with an ordinary one-way ANOVA (**P* < 0.05; ***P* < 0.01). Error bars represent mean ± SEM.

 © 2022 The Authors

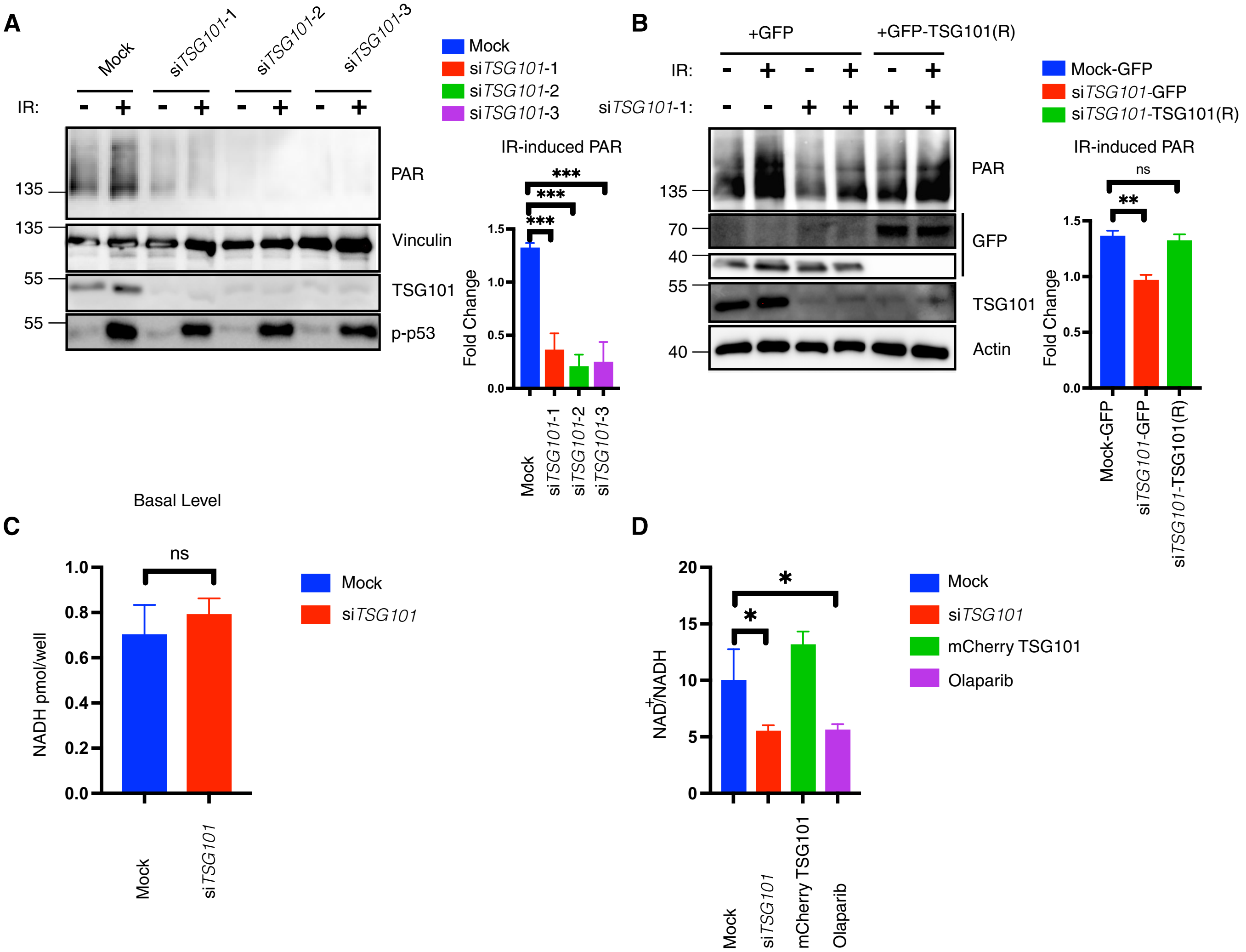

Figure EV3. TSG101 is essential for PARylation.

A U2-OS cells were transfected with nontargeting (mock) or three different *TSG101*-targeting siRNAs. Cells were irradiated (20 Gy) 10 min before analysis. Nuclear extracts were immunoblotted with the indicated reagents and antibodies. Right panel, densitometric measurements of irradiation-induced fold changes in PARylation were obtained from three independent experiments and conditions were compared with an ordinary one-way ANOVA (***$P < 0.001$). Error bars represent mean ± SD.

B A rescue experiment was performed using a siRNA-resistant GFP-TSG101(R) expression vector. Two base substitutions were introduced into the codons for amino acids 61 and 62 of TSG101. U2-OS cells were transfected with either GFP alone or GFP-TSG101 (R) vector. Cells were then transfected with nontargeting or *TSG101*-targeting siRNAs as indicated and were irradiated (20 Gy) 10 min before analysis. Nuclear extracts were immunoblotted with the indicated reagents and antibodies. Densitometric measurements of irradiation-induced fold changes in PARylation (right panel) were obtained from three independent experiments. Conditions were compared with an ordinary one-way ANOVA (ns, $P > 0.05$; **$P < 0.01$). Error bars represent mean ± SD.

C U2-OS cells were transfected with nontargeting or *TSG101*-targeting siRNAs. NADH measurements were performed 72 h after the siRNA transfection. Conditions were compared with Student's *t*-test (Welch Correction) (ns, $P > 0.05$). Error bars represent mean ± SD. Results were obtained from three biologically independent experiments.

D U2-OS cells were transfected with nontargeting (Mock) or *TSG101*-directed siRNAs, with mCherry-*TSG101*, or treated with olaparib (10 μM, 16 h before irradiation), as indicated. Cells were irradiated (20 Gy) 10 min before analysis and NAD+/NADH levels were determined. The result is representative of four independent experiments. The conditions were compared with an ordinary one-way ANOVA (*$P < 0.05$). Error bars represent mean ± SD.

© 2022 The Authors

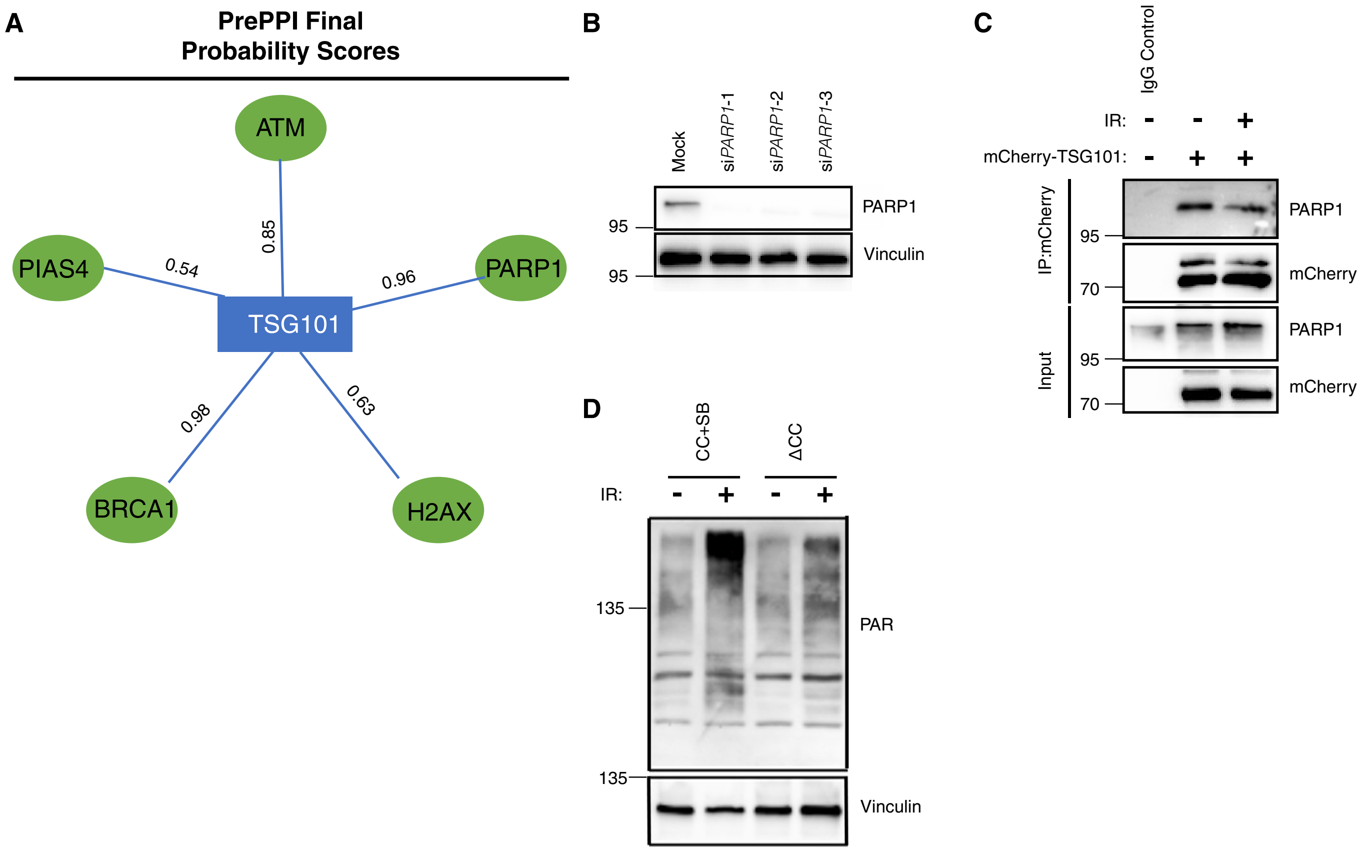

Figure EV4. TSG101 interacts with and enzymatically activates PARP1.

A Final protein–protein interaction probability scores for the indicated interactions were obtained from the PrePPI database (Zhang *et al*, 2013).

B Validation of PARP1 knockdown in A. Different PARP1 siRNAs were used in each independent PLA experiment summarized in Fig 4B. The representative image in Fig 4A was obtained using siPARP1(3).

C U2-OS cells were transfected with the full-length mCherry-tagged TSG101 plasmid and DNA damage was induced by irradiation (20 Gy, 45 min before analysis), as indicated. Immunoprecipitation of mCherry was performed using whole-cell extracts. The result is representative of three biologically independent experiments.

D TSG101 deletion constructs CC+SB and ΔCC were expressed in U2-OS cells. Untreated or irradiated cells were harvested (90 min after 20 Gy exposure) and analyzed by Western blotting for PAR levels using the Pan ADPr reagent. Vinculin was detected as a loading control.

 © 2022 The Authors

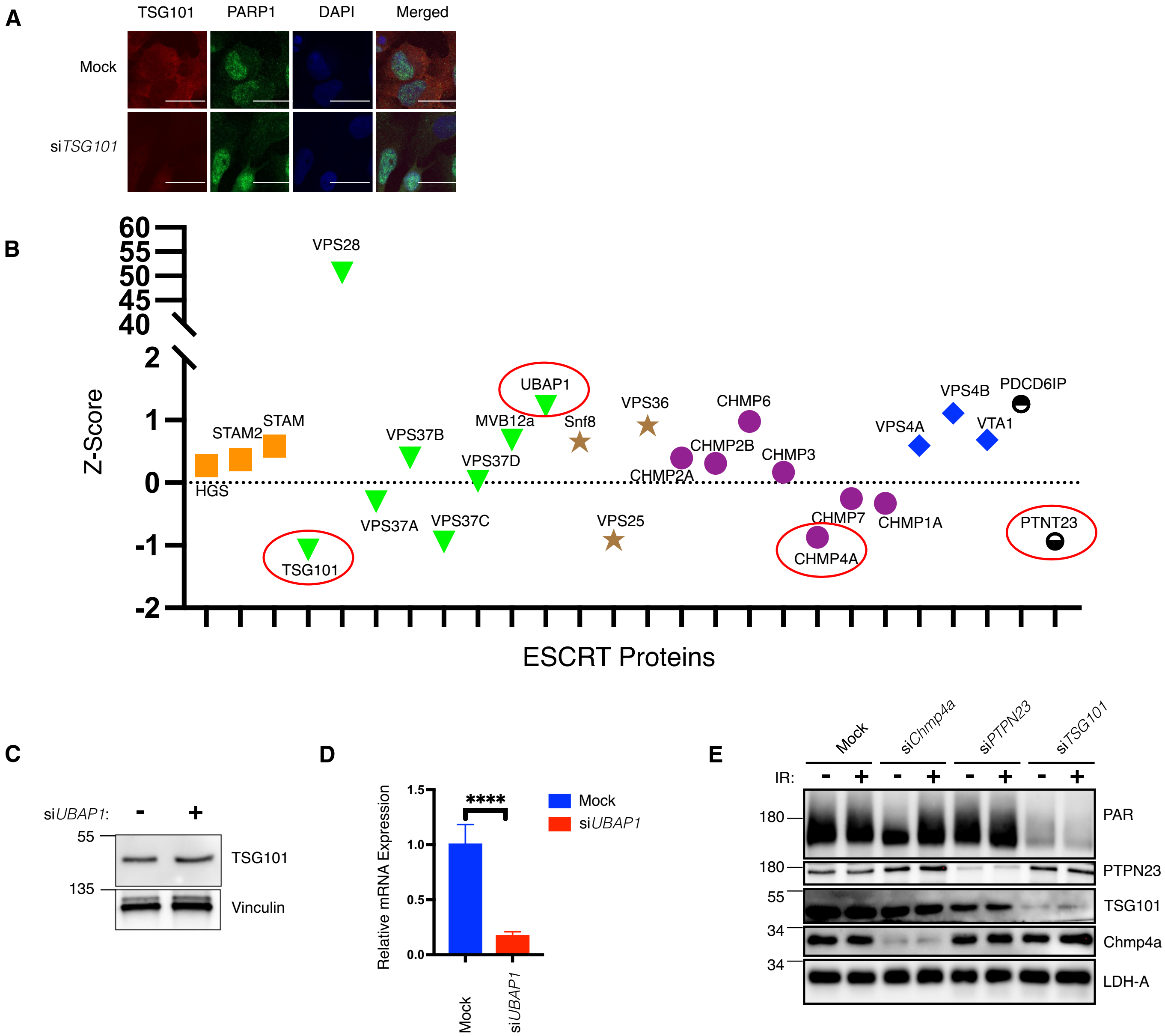

Figure EV5. Control of PARylation by TSG101 is ESCRT complex-independent.

A U2-OS cells were transfected with nontargeting (mock) or *TSG101*-targeting siRNAs. Indirect immunofluorescence visualizes TSG101 (red) and PARP1 (green). DAPI staining shows the nuclei in blue. Scale bar is 10 μm. The image is representative of three biologically independent experiments.

B Z-scores of the mean values of the depicted ESCRT complex members and accessory proteins (ESCRT-0: orange, ESCRT-I: green, ESCRT-II: brown, ESCRT-III: purple, and ESCRT-IV: red) from Dataset EV1. Subsequently analyzed members CHMP4A, PTPN23, TSG101, and UBAP1 are highlighted with red circles.

C U2-OS cells were transfected with nontargeting or *UBAP1*-targeting siRNAs. Whole-cell extracts were immunoblotted with antibodies against the indicated proteins. Vinculin was used as a loading control.

D Total RNA was extracted from cells analyzed in B and converted to cDNA. The relative mRNA expression of *UBAP1* was normalized to two housekeeping genes, *RPL13A*, and *TBP2*. Data are from six biologically independent experiments. Conditions were compared with an unpaired *t*-test with Welch's correction (****$P < 0.0001$). Error bars represent mean ± SD.

E U2-OS cells were transfected with nontargeting siRNAs (mock) or with siRNAs directed against *CHMP4A*, PTPN23, or TSG101 and irradiated (20 Gy) or not, as indicated. PAR levels and expression of indicated proteins were monitored by western blotting with the respective agents and antibodies. LDH-A served as a loading control.

Figure EV6. Efficient apoptosis protection and DNA repair following DNA double strand break generation requires TSG101.

A U2-OS cells were lentivirally transduced with two independent guide RNAs targeting the *TSG101* locus. Due to the lethal side effects of TSG101 deletion on long-term proliferation, bulk cells were used instead of the clonally expanded cells. DNA damage was induced by irradiation (20 Gy, 3 or 9 h before analysis) in wild-type or TSG101 CRISPR knockout bulk cells. Total mRNA was extracted and expression of *NUAK*, *PTX3*, and *PUMA* was analyzed with RT–qPCR. The mRNA expression was normalized to the housekeeping genes *ACTA1*, *RPL13A*, and *TBP2*. Data are from three biologically independent experiments. The conditions were compared with an ordinary one-way ANOVA (***$P < 0.001$; ****$P < 0.0001$). Error bars represent mean ± SEM.

B The efficiency of *TSG101* CRISPR in the cells used in A is shown with immunoblotting. Whole-cell extracts were immunoblotted with antibodies against TSG101. Vinculin was used as a loading control.

C Irradiation-induced cleaved caspase-3 activation in nontargeting gRNA or *Tsg101*-targeting gRNA transduced MEF cells was measured using a colorimetric assay. For knockout efficiencies see Fig EV6E. Bulk cells from *Tsg101* guide-1 were used in this assay. Results were obtained from four biologically independent experiments. The conditions were compared with an unpaired *t*-test (****$P < 0.0001$). Error bars represent mean ± SD.

D Percentage of γH2AX positive staining from Fig 6D is shown. Results were obtained from blind counting of approximately 100 cells for each condition from 3 biologically independent experiments. The conditions were compared with an unpaired *t*-test (****$P < 0.0001$). Error bars represent mean ± SD.

E Wild-type MEF cells from Fig 6J (control or *Tsg101* guide RNA transduced cells) were analyzed for *Tsg101* CRSIPR/Cas9 knockout efficiency. Whole-cell extracts were obtained from these cells and immunoblotted with the indicated antibodies. Vinculin was used as a loading control.

F U2-OS cells were transfected with nontargeting (mock) or two different *TSG101*-targeting siRNAs. Nonirradiated cells (samples 1–3) were compared with senescent cells 7 days postirradiation (samples 4–6). Total mRNA was extracted and expression of *CXCL8*, *IL-6*, and *TSG101* was analyzed by RT–qPCR. The mRNA expression of these genes was normalized to the three housekeeping genes *ACTA1*, *RPL13A*, and *TBP2*. Data are from three biologically independent experiments. Conditions were compared with an ordinary one-way ANOVA (ns, $P > 0.05$; *$P < 0.05$; **$P < 0.01$, ***$P < 0.001$). The conditions were compared with an unpaired *t*-test (****$P < 0.0001$). Error bars represent mean ± SEM.

G Representative brightfield images of *TSG101*-targeting or nontargeting siRNA-transfected *BRCA1* wild-type and mutant MDA-MB 231 and 436 breast cancer cells are shown.

 © 2022 The Authors

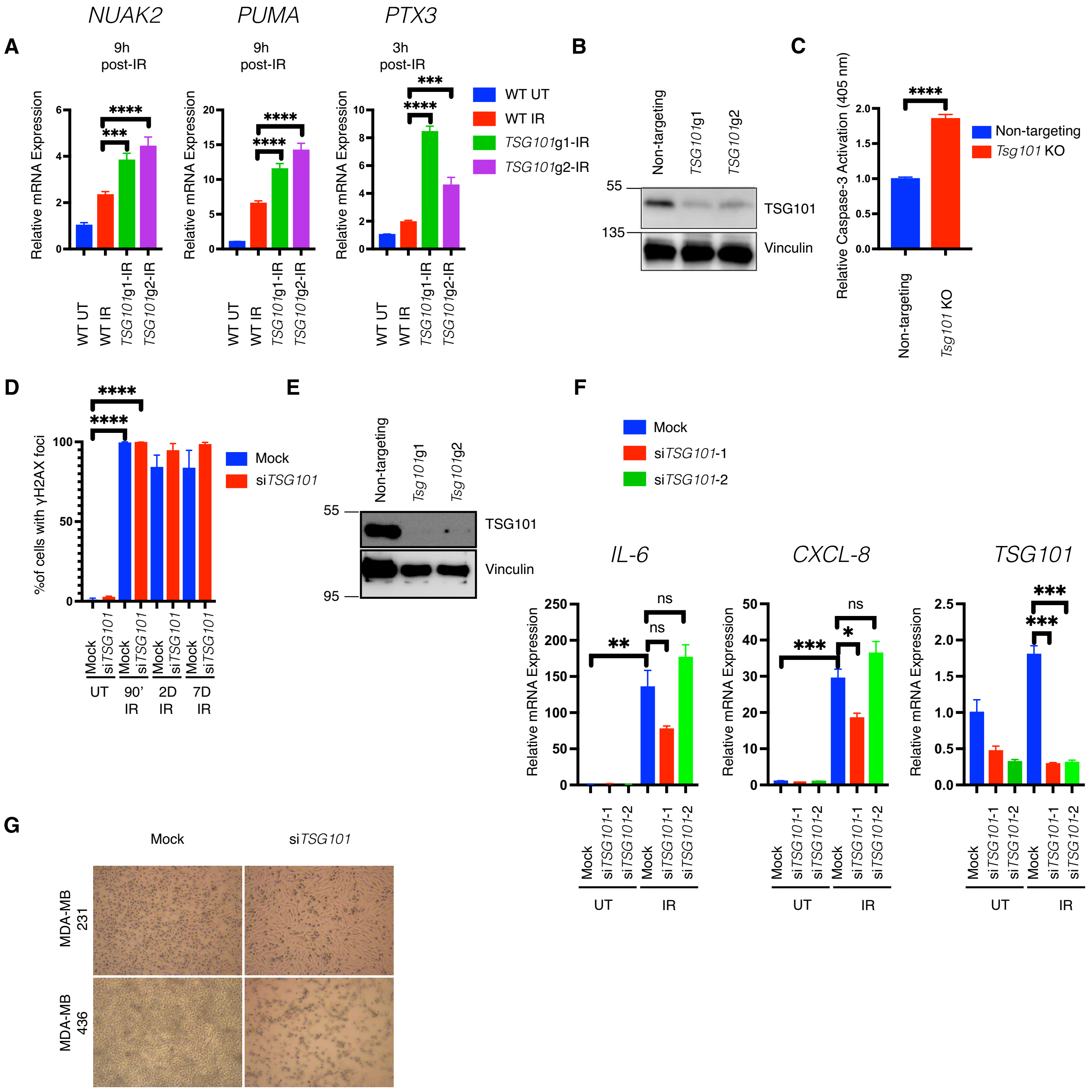
A
NUAK2
9h post-IR
PUMA
9h post-IR
PTX3
3h post-IR
Relative mRNA Expression
WT UT
WT IR
TSG101g1-IR
TSG101g2-IR
B
Non-targeting
TSG101g1
TSG101g2
55
135
TSG101
Vinculin
C
Relative Caspase-3 Activation (405 nm)
Non-targeting
Tsg101 KO
D
%of cells with γH2AX foci
Mock
siTSG101
UT
90' IR
2D IR
7D IR
E
Non-targeting
Tsg101g1
Tsg101g2
55
95
TSG101
Vinculin
F
Mock
siTSG101-1
siTSG101-2
IL-6
CXCL-8
TSG101
Relative mRNA Expression
ns
UT
IR
G
Mock
siTSG101
MDA-MB 231
MDA-MB 436

Figure EV6.
