# Supplementary material for: TSG101 associates with PARP1 and is essential for PARylation and DNA damage‐induced NF‐κB activation
Source: EMBO J. 2022 Sep 20;41(21):e110372. doi: 10.15252/embj.2021110372 (PMC9627669; doi:10.15252/embj.2021110372)
Supplement: Supplementary file 4 — Table EV3 [file EMBJ-41-e110372-s007.docx]

**Table EV3: BRCA1/2 mutation status of breast cancer cell lines**

BRCA1/2 mutations in breast cancer cell lines depicted in Figure 6I and further breast cancer cell lines retrieved from the DepMap database (<https://depmap.org/portal/>).

**CRISPR, TSG101 (DepMap 22Q1 Public+Score, Chronos)**

**List of all 9 cell lines with mutated BRCA1 and/or BRCA2 in the 46 breast cancer cell lines and MDAMB231 wt cells**

| **Cell line** | **BRCA1 status** | **BRCA2 status** | **chronos** |
| --- | --- | --- | --- |
| HCC202 | Damaging frame shift ins, p.K1530fs | WT | -2,04 |
| HCC1395 | Damaging nonsense mut, p.R1751* | Damaging nonsense mut, p.E1593* | -1,60 |
| MDAMB436 | Damaging splice site | WT | -1,59 |
| SUM149PT | Damaging frame shift del, p.N723fs | WT | -1,50 |
| HCC1954 | Damaging frame shift del, p.V1810fs | WT | -1,38 |
| MDAMB468 | WT | Missense, p.M965I, cons. | -1,19 |
| HCC38 | WT | Missense, p.K644R, cons. | -1,11 |
| MDAMB231 | WT | WT | -1,09 |
| KPL1 | WT | Missense, p.S1432N | -0,92 |
| CAL51 | WT | Damaging frame shift del, p.E984fs | -0,86 |
