# Supplementary material for: TSG101 associates with PARP1 and is essential for PARylation and DNA damage‐induced NF‐κB activation
Source: EMBO J. 2022 Sep 20;41(21):e110372. doi: 10.15252/embj.2021110372 (PMC9627669; doi:10.15252/embj.2021110372)
Supplement: Supplementary file 8 — Movie EV3 [file EMBJ-41-e110372-s003.zip › Legend to Movie EV3.docx]

**Legend to Movie EV3**

Movie of PARP1-GFP association with a laser-microirradiation site in si*TSG101*-transfected U2-OS cells, representative for figures 5B and 5C.
