# Supplementary material for: TSG101 associates with PARP1 and is essential for PARylation and DNA damage‐induced NF‐κB activation
Source: EMBO J. 2022 Sep 20;41(21):e110372. doi: 10.15252/embj.2021110372 (PMC9627669; doi:10.15252/embj.2021110372)
Supplement: Supplementary file 14 — PDF+ [file EMBJ-41-e110372-s002.pdf]

Article

TRANSPARENT PROCESS   OPEN ACCESS

THE EMBO JOURNAL

TSG101 associates with PARP1 and is essential for PARylation and DNA damage-induced NF-κB activation

Ahmet Buğra Tufan[1,†], Katina Lazarow[2], Marina Kolesnichenko[1,3], Anje Sporbert[4], Jens Peter von Kries[2] & Claus Scheidereit[1,*]

Abstract

In a genome-wide screening for components of the dsDNA-break-induced IKK-NF-κB pathway, we identified scores of regulators, including tumor susceptibility gene TSG101. TSG101 is essential for DNA damage-induced formation of cellular poly(ADP-ribose) (PAR). TSG101 binds to PARP1 and is required for PARP1 activation. This function of TSG101 is independent of its role in the ESCRT-I endosomal sorting complex. In the absence of TSG101, the PAR-dependent formation of a nuclear PARP1-IKKγ signalosome, which triggers IKK activation, is impaired. According to its requirement for PARP1 and NF-κB activation, TSG101-deficient cells are defective in DNA repair and apoptosis protection. Loss of TSG101 results in PARP1 trapping at damage sites and mimics the effect of pharmacological PARP inhibition. We also show that the loss of TSG101 in connection with inactivated tumor suppressors BRCA1/2 in breast cancer cells is lethal. Our results imply TSG101 as a therapeutic target to achieve synthetic lethality in cancer treatment.

Keywords ADP ribosylation; breast cancer; DNA damage; IKK-NF-κB pathway; TSG101
Subject Categories DNA Replication, Recombination & Repair; Post-translational Modifications & Proteolysis; Signal Transduction
DOI 10.15252/embj.2021110372 | Received 6 December 2021 | Revised 11 August 2022 | Accepted 25 August 2022 | Published online 20 September 2022
The EMBO Journal (2022) 41: e110372

Introduction

Endogenous and exogenous sources of DNA damage compromise the genomic integrity of organisms (Jackson & Bartek, 2009). To prevent genome instability or tumorigenesis, cells developed a highly conserved DNA damage response (DDR) that coordinates a network of signaling pathways required for sensing and repairing damaged DNA (Goldstein & Kastan, 2015). Several effector proteins and post-translational modifications (PTM) tightly regulate the DDR (Polo & Jackson, 2011). ADP-ribose modification is one of the most crucial regulatory PTMs found in eukaryotes (Gibson & Kraus, 2012). The poly(ADP-ribose) polymerase (PARP) family of enzymes catalyzes ADP ribosylation by using NAD^+ as a substrate (D'Amours *et al*, 1999) and generates the highly negatively charged poly(ADP-ribose) (PAR) polymer (Luo & Kraus, 2012). Poly(ADP-ribose) polymerase 1 (PARP1) is the principal PARP that is involved in DNA damage signaling and produces most cellular PAR (Kim *et al*, 2005). PARP1 is activated by binding to DNA strand breaks and catalyzes PARylation of target proteins, including itself, histones, and chromatin-associated proteins (Mortusewicz *et al*, 2007). Enrichment of PAR near the DNA damage site serves as a docking platform for the recruitment of DNA repair factors (Krishnakumar & Kraus, 2010). PARP1 auto-PARylation inhibits its DNA binding capacity and leads to its release from the DNA lesions into the nucleoplasm where it initiates further downstream signaling events (Ahel *et al*, 2009).

As part of the PARP1-mediated genotoxic stress response, cells activate the NF-κB pathway (Stilmann *et al*, 2009). Following activation, auto-modified PARP1 forms a transient nuclear complex with IKKγ, PIASy, and ATM (Stilmann *et al*, 2009). IKKγ is sequentially SUMOylated and phosphorylated in the signalosome complex by PIASy- and ATM (Huang *et al*, 2001; Mabb *et al*, 2006; Stilmann *et al*, 2009). Shortly after the IKKγ modifications, both ATM and IKKγ are exported to the cytoplasm (Wu *et al*, 2006). ATM triggers autoubiquitination of TRAF6, which recruits cIAP1 and TAB2-TAK1, leading to TAK1-dependent activation of IKKβ (Hinz *et al*, 2010). After IKK activation, the downstream signaling events occur similar to the classical NF-κB cascade, in which phosphorylation of IκBα by IKK is followed by its ubiquitination and proteasomal degradation, permitting liberated NF-κB dimers to translocate to the nucleus (Hinz *et al*, 2010; Wang *et al*, 2017).

The NF-κB pathway upregulates the expression of anti-apoptotic genes, including *Bcl-XL* (Stilmann *et al*, 2009). Although DNA

1 Laboratory for Signal Transduction in Tumor Cells, Max Delbrück Center for Molecular Medicine in the Helmholtz Association (MDC), Berlin, Germany
2 Leibniz-Forschungsinstitut for Molecular Pharmacology (FMP), Berlin, Germany
3 Department of Hepatology and Gastroenterology, Charité-Universitätsmedizin Berlin, Berlin, Germany
4 Max Delbrück Center for Molecular Medicine in the Helmholtz Association (MDC), Advanced Light Microscopy Technology Platform, Berlin, Germany
*Corresponding author (Lead Contact). Tel: +49 30 9406 3816; E-mail: scheidereit@mdc-berlin.de
†Present address: Department of Biological Chemistry and Molecular Pharmacology, Harvard Medical School, Boston, MA, USA

© 2022 The Authors. Published under the terms of the CC BY 4.0 license.

damage-induced NF-κB has crucial physiological roles, important steps in this signaling cascade are still unexplored. Identification of essential regulators of the DNA damage-induced IKK-NF-κB pathway is the basis for the development of directed cancer therapeutics.

Here, we systemically identified regulators of the genotoxic stress-induced NF-κB pathway by using genome-wide siRNA screens. Among numerous candidate regulators, we have identified Tumor Susceptibility Gene 101 (TSG101) as an essential component of the DNA damage-induced NF-κB pathway and of PARP1 function in the DNA damage response in general. TSG101 is a member of the endosomal sorting complexes required for the transport complex I (ESCRT-I) (Ferraiuolo *et al*, 2020); however, its role in the DDR was unexpected. Via biochemical pathway mapping of TSG101 in the IKK-NF-κB signaling cascade, we discovered that TSG101 is essential for enzymatic activation of PARP1. TSG101 interacts through its coiled-coil domain with PARP1 and stimulates PARylation *in vitro* and intact cells. Depletion of TSG101 completely abrogates cellular PARylation and causes trapping of PARP1 in DNA lesions due to loss of its auto-PARylation. Because of failed PARylation of PARP1, γH2AX foci indicating unrepaired DNA damage accumulate. TSG101 is required for activation of the PAR-dependent IKK-NF-κB pathway by DNA damage. Targeting the TSG101-PARP1 axis sensitizes cells to apoptosis due to impaired anti-apoptotic NF-κB driven gene expression. We also demonstrate a co-dependency of TSG101 and BRCA1/2 for the survival of breast cancer cells, similar to the synthetic lethality observed for PARP inhibitor olaparib and BRCA1/2 deficiency.

Results

Systematic identification of essential components and regulators of the DNA damage-induced NF-κB pathway via genome-wide siRNA screens

For a systematical identification of regulators required for the DNA damage-induced NF-κB pathway, we performed a high-content genome-wide siRNA screen. We used a transcription activity-based luciferase assay as a readout for stimulus-dependent NF-κB pathway activation (Figs 1A and EV1A). After delivery of the siRNA library, etoposide served as DNA damage-inducing agent to activate NF-κB. Cells transfected with *IKBKG* siRNA, which abolished NF-κB-driven luciferase activity, served as positive controls (Fig EV1B). We selected the 1,000 top hits as candidate activators of the pathway and 100 further hits, which, on the contrary, suppressed NF-κB activation (Fig 1B and Dataset EV1). The putative positive regulatory hits covered many previously described members and regulators of the NF-κB pathway (Fig 1C and Dataset EV1). For example, RELA, a principal transactivating NF-κB subunit, topped the list. Moreover, top hits included ATM, TIFA, CHUK, TRAF6, and IKKγ, all known components of the DNA damage-induced NF-κB pathway (Fig 1C). The other side of the screening curve revealed several previously described negative regulators, whose depletion unleashed NF-κB activation (Fig 1B), including NFKBIA, CYLD, TRAF3, VPS28, and N4BP1. Furthermore, we identified TANK, and SENP1, suppressors specifically of genotoxic stress-induced IKK-NF-κB signaling (see Fig 1C for details). Poly(ADP-ribose) glycohydrolase (PARG) is also classified as a negative regulator. Overall, the screening assay revealed a high degree of robustness ($Z' > 0.5$, Dataset EV2) and technical reproducibility (Fig EV1C).

We further refined the group of 1,000 candidates to 320 (Dataset EV3), by excluding genes encoding members of general transcription or RNA processing machineries, the proteasome, and ribosome, which are generally required for NF-κB-dependent gene expression. The selected 320 hits were subjected to a counter screen with TNFα in comparison with etoposide as inducer. Targeting of known components of the DNA damage-induced IKK-NF-κB pathway, such as *TRAF6*, which have no documented prominent role in TNFα-induced NF-κB signaling, was included to establish an arbitrary threshold (Fig 1D). Following the counter screen, 60 hits remained, which were highly selective for the DNA damage-induced pathway (Dataset EV4). To determine functional protein networks, these hits were analyzed in ENRICHR (Kuleshov *et al*, 2016), REACTOME (Jassal *et al*, 2020), and STRING (Szklarczyk *et al*, 2019) databases. The curated networks revealed that the multifunctional tumor susceptibility gene product TSG101 (Ferraiuolo *et al*, 2020) clusters with several other hits from the primary screen (Fig EV1D), implying a crucial biological function of TSG101 for DNA damage-induced NF-κB signaling.

The candidate hits also included NAA15, an auxiliary subunit of the N-terminal acetyltransferase A (Nat A) complex 15 (Fig EV1E) (Arnesen *et al*, 2005) (Fig 1C), and several mitochondrial enzymes (Table EV2).

TSG101 is essential for the DNA damage-induced NF-κB pathway

To validate the contribution of TSG101 to the DNA damage-induced IKK-NF-κB pathway, we knocked down *TSG101* and, as

Figure 1. Genome-wide siRNA screening.

A Schematic diagram of the two consecutive siRNA screens. NF-κB-driven luciferase activity served as a readout for pathway activation. The primary screen was performed using a genome-wide siRNA library. A total of 320 candidates were selected for a secondary counter screen with TNFα to narrow down DNA damage-specific regulators of the NF-κB pathway. Responsiveness of the reporter cell line to NF-κB activating stimuli was analyzed by western blotting (Fig EV1A).

B Cumulative distribution of Z-scores of the genome-wide siRNA screening. Candidates with low Z-scores are putative positive regulators as their depletion abrogated NF-κB activation by DNA damage (red circle). Putative negative regulators with high Z-scores exhibited elevated NF-κB activity (blue circle).

C Z-scores of the best-scoring siRNAs for selected previously known and newly identified pathway regulators. Hits include VPS28 (Mamińska *et al*, 2016), CYLD (Trompouki *et al*, 2003), NFKBIA (Haskill *et al*, 1991), TRAF3 (Vallabhapurapu *et al*, 2008), N4BP1 (Shi *et al*, 2021), TANK (Wang *et al*, 2015), SENP1 (Lee *et al*, 2011; Shao *et al*, 2015), PARG (Cortes *et al*, 2004), RELA (Li *et al*, 2001), TIFA (Fu *et al*, 2018), CHUK (Colomer *et al*, 2019), TRAF6 (Hinz *et al*, 2010) and IKBKG (Mabb *et al*, 2006) for expected factors.

D Representative relative luciferase units (RLU) of the differential counter screen of the 320 preselected hits are shown. The RLU of hits is displayed for etoposide and TNFα screens (left and right panels). See Dataset EV4 for details. RLU levels of treatment controls are indicated with red lines. Hits that abrogated etoposide-induced NF-κB activity were classified as group I. Hits in the TNFα counter screen with higher activity than in si*TRAF6*-transfected cells constituted group II. The intersection of the two groups indicates putative DNA damage-selective positive regulators. Note that TNFα may trigger secondary NF-κB activation events through autocrine/-paracrine mechanisms. See Dataset EV4 for luminescence values.

 © 2022 The Authors

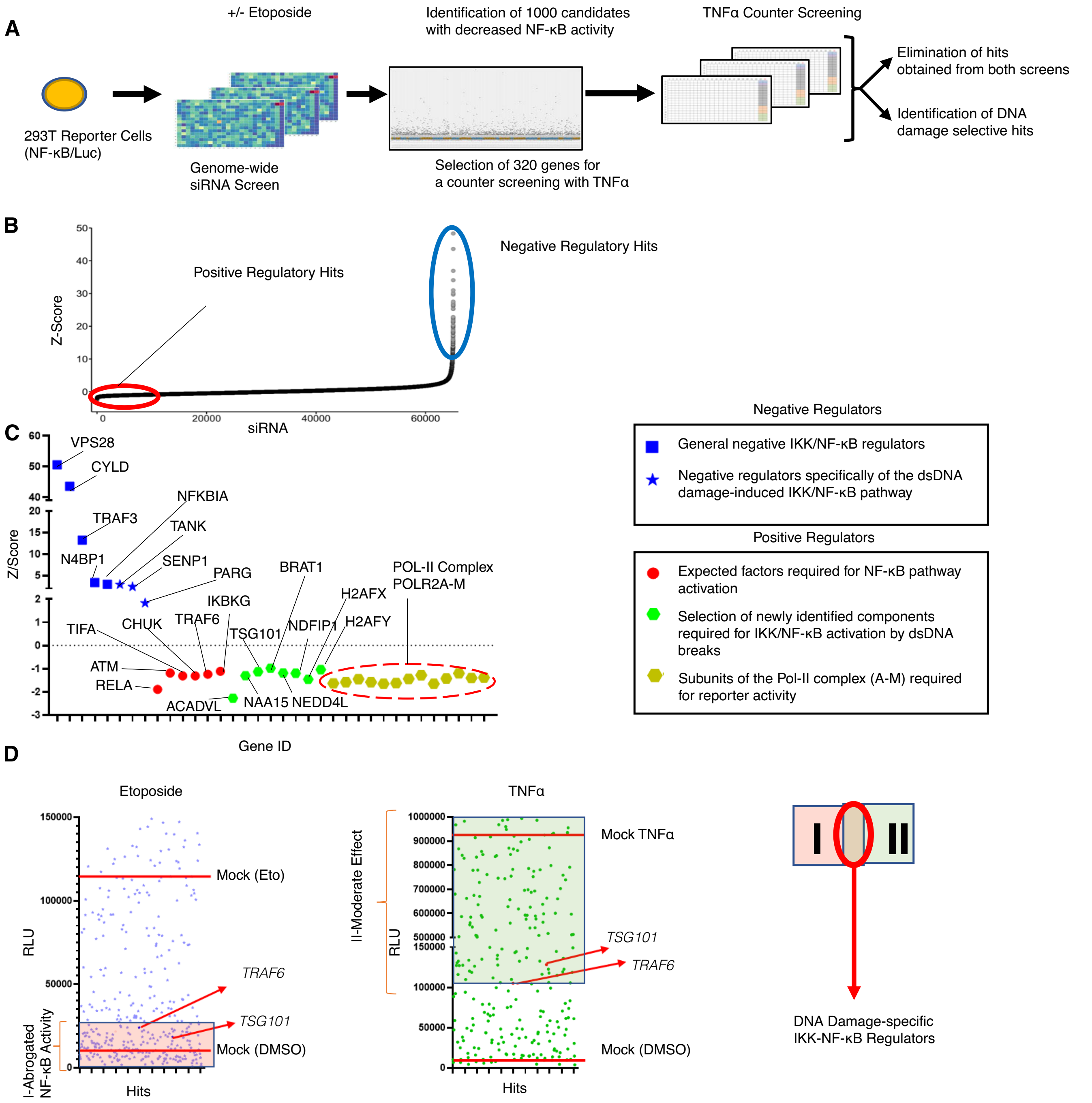

Figure 1.

a positive control, *ATM* (Piret *et al*, 1999). Indeed, depletion of TSG101 or ATM equivalently abrogated NF-κB-driven reporter gene activation (Figs 2A and EV2A). Furthermore, IKK-dependent phosphorylation of p65 at serine 536, a key step in NF-κB activation after DNA damage (Kolesnichenko *et al*, 2021), was abolished in TSG101 or ATM-deficient cells (Fig 2B). Importantly, the *TSG101* knockdown did not affect activation or protein level of ATM (Fig 2B). As a second line of evidence, knockdown TSG101 by two additional siRNAs also resulted in complete loss of DNA damage-induced p65 phosphorylation (Fig EV2B) and ruled out possible off-target effects of the siRNAs.

TSG101 is required in the same manner as ATM for the induced expression of selected NF-κB target genes (*CCL2*, *CXCL10*, *ICAM1*, *CXCL8*, and *NFKBIA*) following irradiation (Fig 2C) or etoposide treatment (Fig EV2C). In agreement with these results, the knockdown of *TSG101* blocked genotoxic stress-induced DNA binding activity of NF-κB (Fig 2D). Taken together, our data demonstrate that TSG101 is essential for the activation of the NF-κB pathway by DNA damage.

TSG101 is required for PARylation

The formation of a transient nuclear PARP1 signalosome complex involving ATM, IKKγ, and PIASy (Stilmann *et al*, 2009) and a cytoplasmic ATM-TRAF6 module, where ATM is exported to the cytoplasm (Hinz *et al*, 2010) are cornerstones of the DNA damage-induced IKK pathway. We next analyzed how and where TSG101 acts on this pathway. To investigate whether TSG101 affects ATM export, we prepared nuclear and cytoplasmic fractions. In cells treated with etoposide, loss of TSG101 influenced neither activation of nuclear ATM, nor cytoplasmic export of phosphorylated ATM (Fig 3A). Likewise, pharmacological inhibition of PARP1 did not impair ATM export. These data indicate that TSG101 does not function upstream of the cytoplasmic export of phosphorylated ATM.

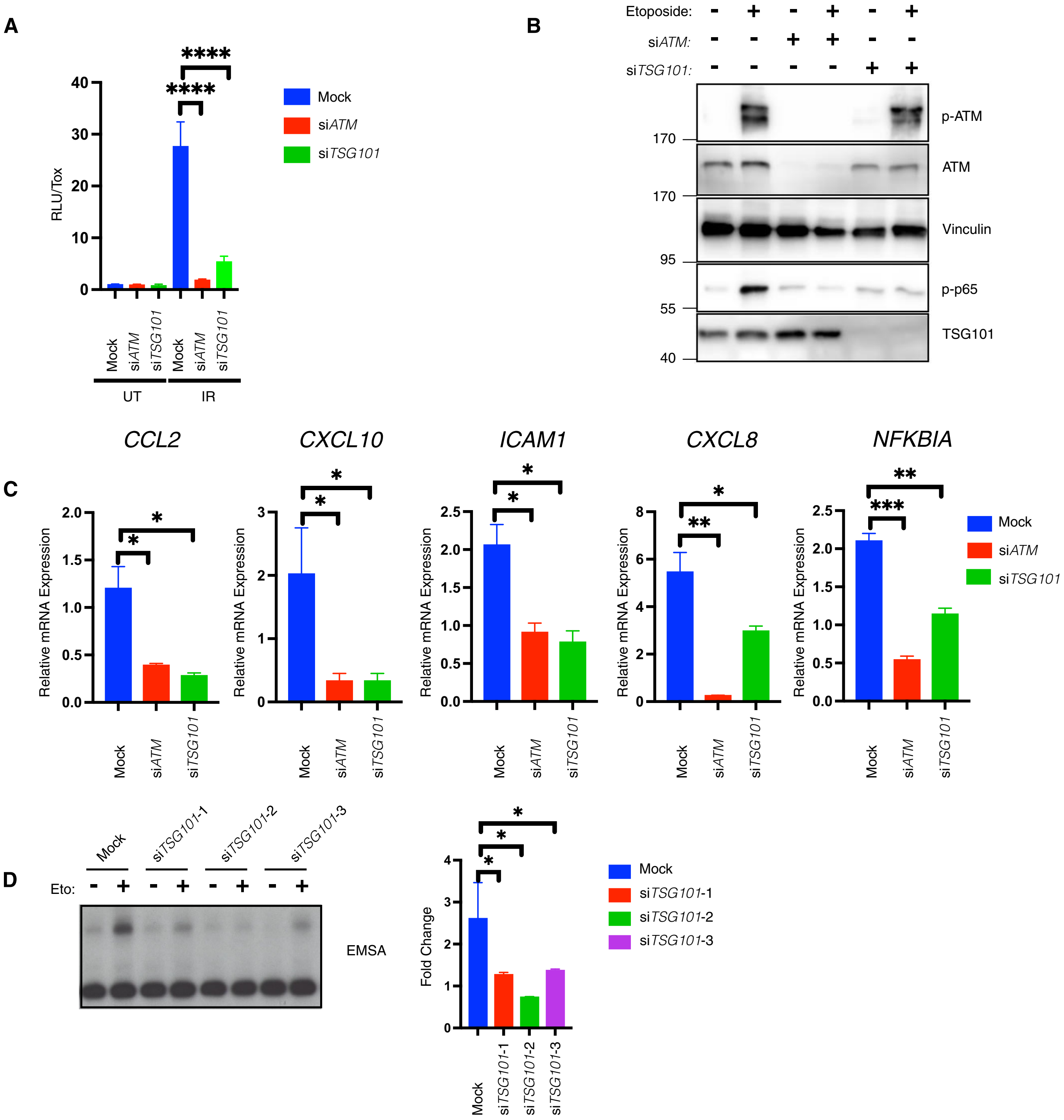

Figure 2.

 © 2022 The Authors

Figure 2. TSG101 is essential for the DNA damage-induced NF-κB pathway.

A IR-induced NF-κB pathway activation was analyzed via luciferase activity of HEK-Luc cells, transfected with the indicated siRNAs. The indicated cells were irradiated (20 Gy, 8 h before analysis). A late time point was used for efficient luciferase expression. Luciferase activity was normalized to the viable cell number (TOX fluorescence). The data represent three biological and five technical replicates. Error bars represent mean ± SD. For knockdown efficiency, see Fig EV2A. The conditions were compared with an ordinary one-way ANOVA (****$P < 0.0001$).

B U2-OS cells were transfected with the indicated siRNAs. The indicated cells were treated with etoposide (50 μM, 90 min. Before analysis). Whole-cell extracts were immunoblotted with the indicated antibodies. The result is representative of three biological replicates.

C U2-OS cells were transfected with the indicated siRNAs and exposed to irradiation (20 Gy, 90 min. Before analysis). The expression of indicated genes was analyzed using RT–qPCR. The mRNA expression was normalized to the expression of the housekeeping genes *ACTA1*, *RPL13A*, and *TBP2*. Data represent three biologically independent experiments +/− SEM. The conditions were compared with an ordinary one-way ANOVA (*$P < 0.05$; **$P < 0.01$, ***$P < 0.001$).

D U2-OS cells were transfected with nontargeting (mock) or *TSG101*-targeting siRNAs and irradiated (20 Gy) for 90 min. Before analysis. Lysates were analyzed by EMSA and results of fold changes in densitometric measurements of the three independent experiments were compared with an ordinary one-way ANOVA (*$P < 0.05$) in the right panel. Error bars represent mean ± SD.

The DNA damage-induced formation of the PARP1 signalosome is highly dependent on PARP1 auto-PARylation, which mediates the interaction between PARP1, ATM, IKKγ, and PIASy (Stilmann *et al*, 2009). Strikingly, in the absence of TSG101, etoposide-induced PARylation was significantly impaired (Fig 3A), implying that TSG101 regulates the DNA damage-induced NF-κB pathway by enabling the formation of the transient PARP1 signalosome complex. Furthermore, depletion of TSG101 led to a complete loss of irradiation-induced PARylation (Fig 3B). Knockdown of TSG101 by two additional siRNAs also led to diminished DNA damage-induced PARylation (Fig EV3A). In agreement with these observations, both basal level and DNA damage-induced mono- or poly-(ADP-ribose) modification of histones was severely diminished in the absence of TSG101 (Fig 3C). Off-target effects were excluded since ectopic expression of TSG101 from an siRNA-resistant construct rescued DNA damage-induced PARylation in TSG101-depleted cells (Fig EV3B). Depletion of TSG101 does not have any effect on cellular NADH levels in nonirradiated cells (Fig EV3C), ruling out that any mechanisms controlling NADH/NAD+ availability required for PARylation have been impaired. Upon irradiation, however, depletion of TSG101 or addition of olaparib decreased the NAD+/NADH ratio, while ectopic TSG101 expression caused an increase (Fig EV3D).

As a member of the ESCRT-I complex, depletion of TSG101 destabilizes the early endosome morphology and dynamics at a wide range (Doyotte *et al*, 2005). Indeed, it was previously shown that Vacuolar Protein Sorting 28 (VPS28), another member of the ESCRT-I complex (Babst *et al*, 2000), is diminished in cells lacking TSG101 (Doyotte *et al*, 2005). VPS28 protein depletion by siRNA expression also strongly reduced TSG101 protein levels (Fig 3D), without affecting TSG101 mRNA levels (Fig 3E), indicating mutual protein stability regulation. During the preparation of this manuscript, (Kolmus *et al*, 2021) reported a cross-destabilization of various ESCRT-I components, including downregulation of TSG101 upon VPS28 knockdown, supporting our conclusion. Consistent with our previous data, irradiation-induced PARylation was impaired upon VPS28 depletion-mediated reduction in TSG101 levels (Fig 3F). Collectively, our data show therefore that TSG101 functions like an essential cofactor for PARP1 and regulates the DNA damage-induced NF-κB pathway activation by enabling PAR-dependent interactions between ATM, PARP1, IKKγ, and PIASy.

TSG101 interacts with and activates PARP1

To investigate how TSG101 may control PARylation we analyzed physical interactions between TSG101 and PARP1. TSG101 was predicted to interact with PARP1 and with several members of the PARP1 signalosome complex (Fig EV4A). We also previously identified TSG101 as an irradiation-enhanced, likely PARP1-mediated interaction partner of IKKγ in a proteome-wide SILAC-based IP analysis (Mikuda *et al*, 2018). With a proximity ligation assay (PLA) we were able to show a nuclear interaction between endogenous TSG101 and PARP1 (Figs 4A and B, and EV4B). Complex formation was enhanced following DNA damage induction by etoposide (Fig 4A and B) and was independent from PARylation, as it was insensitive to the PARP inhibitor olaparib (Fig 4A and B). Interestingly, the abundance of the nuclear TSG101-PARP1 interaction was enhanced upon pharmacological inhibition of PARylation (Fig 4B). We also observed that ectopically expressed TSG101 interacts with PARP1 (Fig EV4C).

We then determined the minimal region of TSG101 necessary for PARP1 interaction and found that the coiled-coil (CC) domain of TSG101 is essential for its association with PARP1 (Fig 4C and D). To investigate whether increased levels of TSG101's PARP1-interacting portion have any impact on cellular PARylation, we tested the effect of this domain on irradiation-induced consumption rates of NADH (Fig 4E). Strikingly, we observed that expression of the CC + SB domains of TSG101, but not of the CC-deleted TSG101 version ΔCC, significantly enhanced the NADH consumption (Fig 4E). Similar to overexpression of full-length TSG101 (Fig EV3D), CC+SB, but not ΔCC, caused an elevated NAD^+/NADH ratio (Fig 4E). Consistent with these observations, expression of the CC+SB, but not the ΔCC construct significantly enhanced PARylation (Fig EV4D). To examine the effect of TSG101 on the catalytic activity of PARP1 directly, we performed an *in vitro* PARylation assay. As expected, PARylation was catalyzed upon incubation of recombinant PARP1 with NAD^+, $MgCl_2$, and sheared salmon sperm DNA (Fig 4F). Strikingly, the addition of TSG101, unlike equivalent amounts of other unrelated proteins, such as BSA or RNF113A, strongly enhanced the catalytic activity of PARP1 (Fig 4F). Collectively, our data suggest that the interaction between PARP1 and TSG101 is functionally crucial for PARylation.

TSG101 prevents trapping of PARP1 in DNA lesions

Rapid recruitment of PARP1 to the DNA lesions enables its direct interaction with the DNA breaks, which triggers its enzymatic activity (Mortusewicz *et al*, 2007). Therefore, TSG101 might be required either for the recruitment of PARP1 to DNA damage sites or for its dissociation. To gain insights into the mechanism of how TSG101 controls these processes, we analyzed the dynamics

© 2022 The Authors

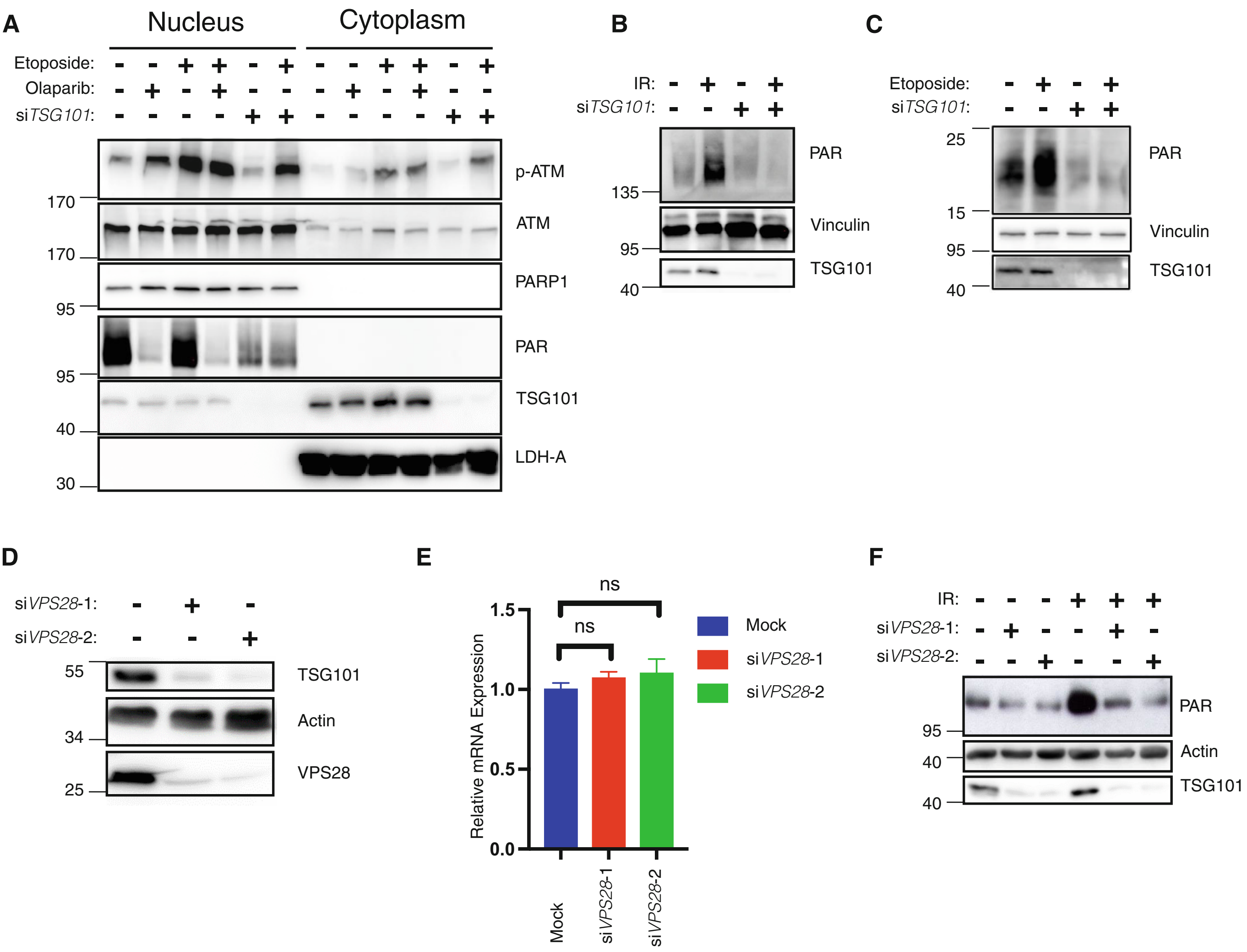

Figure 3. TSG101 controls PARylation.

A Nuclear-cytoplasmic fractionation of U2-OS cells. Indicated samples were transfected with siRNAs against *TSG101*. PARylation was inhibited by olaparib (10 µM, 24 h before analysis). Cytoplasmic export of p-ATM was induced by etoposide (50 µM, 45 min before analysis). Nuclear and cytoplasmic fractions were immunoblotted with the indicated antibodies. The fractionation efficiency was controlled by the respective subcellular marker proteins (PARP1 and LDH-A). PARylation was detected using the Pan ADPr reagent. Note that PARylation is activated by shearing forces during extract preparation (lane 1) (Jungmichel *et al*, 2013).

B U2-OS cells were transfected with nontargeting (−) or *TSG101*-targeting (+) siRNAs and cells were irradiated (+) or not (−) as indicated (20 Gy) 10 min. Before analysis. Whole-cell extracts were immunoblotted with the 10H PAR monoclonal antibody.

C siRNA-transfected U2-OS cells as in B were etoposide-treated or not, as indicated. Whole-cell extracts were immunoblotted with the Pan ADPr reagent that recognizes both poly- and mono-(ADP-ribose). Note that PARylation also occurred during cell lysis in untreated cells.

D U2-OS cells were transfected with nontargeting or *VPS28*-targeting siRNAs. Whole-cell extracts were immunoblotted with indicated antibodies.

E Total mRNA was extracted from the samples in D. Expression of *TSG101* was analyzed by RT–qPCR and normalized to two housekeeping genes, *ACTA1* and *RPL13A*. The conditions were compared with an ordinary one-way ANOVA (ns, $P > 0.05$). Error bars represent mean ± SEM.

F Cells were irradiated (20 Gy) as indicated 10 min before analysis. Whole-cell extracts were immunoblotted with the indicated antibodies.

Data information: The results are each presentative for three (panels A–D and F) or six (panel E) independent experiments.

of PARP1 enrichment at DNA lesions upon laser microirradiation by live-cell imaging (Fig 5A). PARP1 rapidly accumulated at the DNA damage sites in control cells and in cells with TSG101 knockdown (Fig 5B). While PARP1 foci showed dissociation within minutes after microirradiation in control cells, it remained captured on DNA lesions in both si*TSG101*-transfected and olaparib-treated cells (Fig 5B). Normal recruitment of PARP1 to DNA lesions in the absence of TSG101 rules out that TSG101 will direct PARP1 to the DNA breaks. A quantitative analysis of the kinetics of PARP1 recruitment revealed that siRNA depletion of TSG101 or PARP inhibition by olaparib does not affect the rapid PARP1 association with DNA lesions. However, both treatments caused an equivalent and significantly elevated PARP1 retention at later time points (Fig 5C and D). We next investigated whether TSG101 would accumulate at DNA damage sites. An indirect immunofluorescence analysis of DNA damage sites (Fig 5E)

 © 2022 The Authors

revealed that TSG101 is abundantly present in the nucleus and evenly distributed throughout the nucleoplasm and including the DNA damage sites (Figs 5F and EV5A). Collectively, our data indicate that due to the requirement of TSG101 for PARylation, PARP1 remains trapped on DNA lesions in its absence.

Based on our screening results we hypothesized that the role of TSG101 in PARylation is independent from its ESCRT-related functions as TSG101 was the sole member of the ESCRT complex that abrogated the DNA damage-induced NF-κB activity (Fig EV5B). We sought to determine whether disruption of the ESCRT machinery

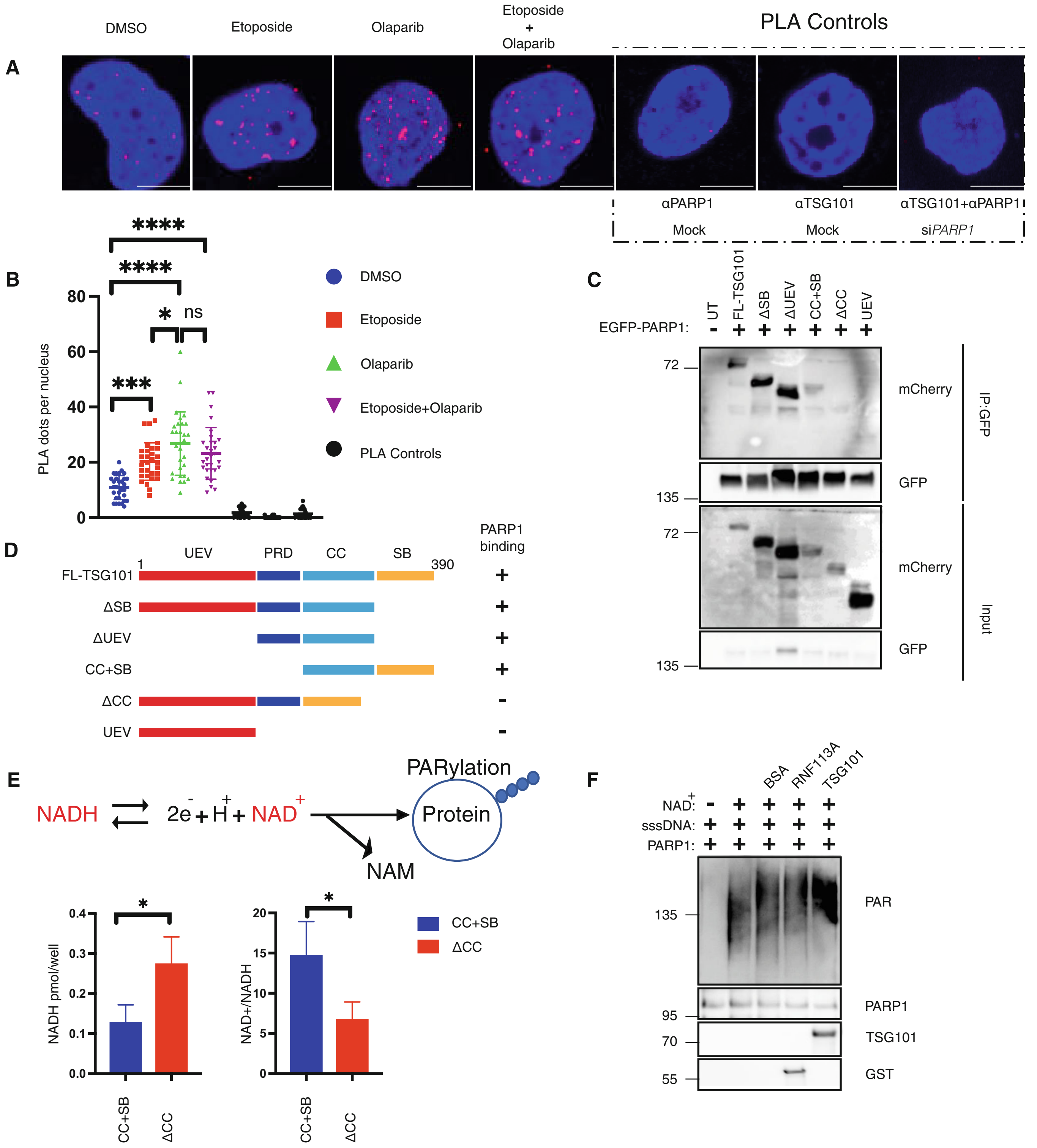

Figure 4.

Figure 4. TSG101 interacts with PARP1.

A Proximity ligation assay (PLA) assay with unstimulated, etoposide (Eto)-treated (50 μM, 10 min. Before analysis), or etoposide plus olaparib-(Olap) co-treated (50 μM of etoposide, 10 min. Before analysis and 10 μM of olaparib 24 h before analysis) U2-OS cells. The red dots represent PARP1-TSG101 interaction. The nucleus is stained with DAPI (blue). Scale bar: 10 μm. As negative controls, PLA was performed without primary PARP1 or TSG101 antibodies. As an additional control, PLA was performed in PARP1 depleted cells to show the specificity of the PLA signal. The data are representative of three biologically independent experiments.

B Quantification of PLA dots per nuclei in (A). Blind counting was performed from 30 replicates of three biologically independent experiments. The conditions were compared with an ordinary one-way ANOVA (ns, $P > 0.05$; *$P < 0.05$; ***$P < 0.001$; ****$P < 0.0001$). Error bars represent mean ± SD.

C U2-OS cells were cotransfected with the indicated deletion constructs of mCherry-tagged TSG101 and EGFP-tagged PARP1. Immunoprecipitation of GFP was performed using whole-cell extracts.

D Diagram showing the summary of PARP1 interacting and noninteracting TSG101 domain deletion mutants.

E U2-OS cells were transfected with the indicated, coiled-coil containing CC+SB and coiled-coil deleted ΔCC constructs. Cells were irradiated (20 Gy, 5 min before analysis) and the decomposed NADH and total NAD^+ levels were quantified. The NADH/NAD^+ ratio is shown in the right panel. The conditions were compared with an unpaired *t*-test (*$P < 0.05$). Error bars represent mean ± SD.

F *In vitro* PARylation reactions were carried out with the indicated recombinant and purified proteins, including 8 ng PARP1 in the presence of ssDNA (sheared salmon sperm DNA) and $MgCl_2$. 100 ng of TSG101 was added to the reaction, as indicated. The PARylation reaction was also carried out using 100 ng of BSA or RNF113A as negative controls.

affects PARylation. Therefore, we analyzed the recruitment kinetics of PARP1 to the DNA lesions following depletion of UBAP1, another component of the ESCRT-I complex (Ferraiuolo *et al*, 2020). Importantly, efficient *UBAP1* knockdown did not affect TSG101 protein level (Fig EV5C and D). Dissociation of PARP1 from the laser microirradiated areas occurred normally in si*UBAP1*-transfected cells (Fig 5G), suggesting that the defective ESCRT system does not influence PARylation. Furthermore, in contrast to TSG101, CHMP4A and PTPN23 were not required for PARylation (Fig EV5B and E). Collectively our data revealed that TSG101's function in PARylation is independent of its role in the ESCRT complex.

Loss of TSG101 sensitizes cells to apoptosis and impairs DNA repair

Since impaired enzymatic PARP1 activation should result in genomic instability and decreased cell viability, we analyzed cell death and DNA damage in TSG101-depleted cells. Notably, the knockdown of TSG101 severely reduced cell viability following etoposide treatment in a time and concentration-dependent manner (Fig 6A). Furthermore, in the absence of TSG101, DNA damage-induced expression of a crucial anti-apoptotic NF-κB target gene, *Bcl-XL* (Stilmann *et al*, 2009), was impaired (Fig 6B). Loss of TSG101 also significantly increased the expression of pro-apoptotic genes, including PUMA (Fig EV6A and B).

Induction of apoptosis was visualized by cleaved caspase-3 (Fig 6C). Strikingly, over 50% of the cells stained positive for cleaved caspase-3 at 7 days postirradiation (Fig 6D). Furthermore, depletion of *Tsg101* in murine cells also increased IR-induced caspase-3 activation (Fig EV6C). Importantly, the early stages of cell death are associated with a size increase in γH2Ax foci (Bonner *et al*, 2008; Solier & Pommier, 2009), and accordingly, puncta-like γH2Ax foci emerged only in irradiated and TSG101-depleted cells at later time points (Figs 6E and F, and EV6D).

Altogether, our data suggest targeting TSG101 sensitizes cells to DNA damage-induced apoptosis. We additionally examined gene expression in cells at 7 days postirradiation. As we recently showed, DNA damage causes biphasic NF-κB activation, with the second phase occurring days after the initial exposure in a PARP1- and IKK-independent manner and driving the expression of senescence-associated-secretory phenotype (SASP) genes (Kolesnichenko *et al*, 2021). Therefore, we analyzed *bona fide* SASP factors IL-6 and IL-8 (Fig EV6F). The expression of these SASP factors was significantly upregulated at 7 days postirradiation irrespective of the presence of TSG101. These data underline that TSG101 does not play a role in the regulation of SASP expression.

A clinically implemented synthetic lethality principle is based on the finding that cancer patients carrying BRCA1/2 mutations are sensitive to inhibition of PARP1 (Helleday, 2011). This prompted us

Figure 5. PARP1 is trapped in DNA lesions in the absence of TSG101.

A Schematic of the live-cell PARP1 recruitment assay. U2-OS cells transfected with GFP-tagged PARP1 were sensitized with DAPI (10 μg/ml, for 10 min). After 405 nm microirradiation, PARP1 recruitment to and dissociation from the DNA lesion was recorded over time.

B Representative images of PARP1-GFP association with laser-microirradiation sites in untreated (mock), olaparib-treated (10 μM, 24 h before analysis) or si*TSG101*-transfected U2-OS cells at indicated times postirradiation. Scale bar: 7.5 μm. The image is representative of nine replicates from three biologically independent experiments. Go to Movies EV1–EV3 for further illustration.

C Kinetics of PARP1-GFP recruitment and dissociation from DNA lesions were measured at times indicated. Nine nuclei were analyzed for each indicated condition from three biologically independent experiments. The data are shown as mean GFP intensity in the microirradiated area ± SEM, normalized to the mean GFP intensity of corresponding whole nuclei. The conditions were compared with an ordinary one-way ANOVA (ns, $P > 0.05$; ***$P < 0.001$).

D Relative PARP1-GFP recruitment to DNA lesions for each indicated condition at 12 min postirradiation. The conditions were compared with an ordinary one-way ANOVA (**$P < 0.01$). Error bars represent mean ± SEM.

E Scheme of indirect immunofluorescence analysis of DNA damage sites. U2-OS cells were GFP-PARP1-transfected and microirradiated as in (A). Cells were fixed shortly after rapid PARP1 recruitment to the DNA lesions (1 min post-microirradiation) and stained for TSG101 by indirect immunofluorescence.

F Indirect immunofluorescence of TSG101 at indicated times post damage. DNA lesions were generated as in B. Stippled lines indicate the applied laser beam. Recruitment of PARP1 to DNA lesions was recorded from live cells. Cells were fixed at 1 min post-microirradiation and stained for TSG101. For specificity of the TSG101 staining see Fig EV5A. Scale bar: 10 μm.

G Representative images of PARP1-GFP association/dissociation at laser-microirradiation sites in untreated (mock) or si*UBAP1*-transfected U2-OS cells at indicated times postirradiation. Scale bar: 10 μm. The image is representative of four independent experiments.

© 2022 The Authors

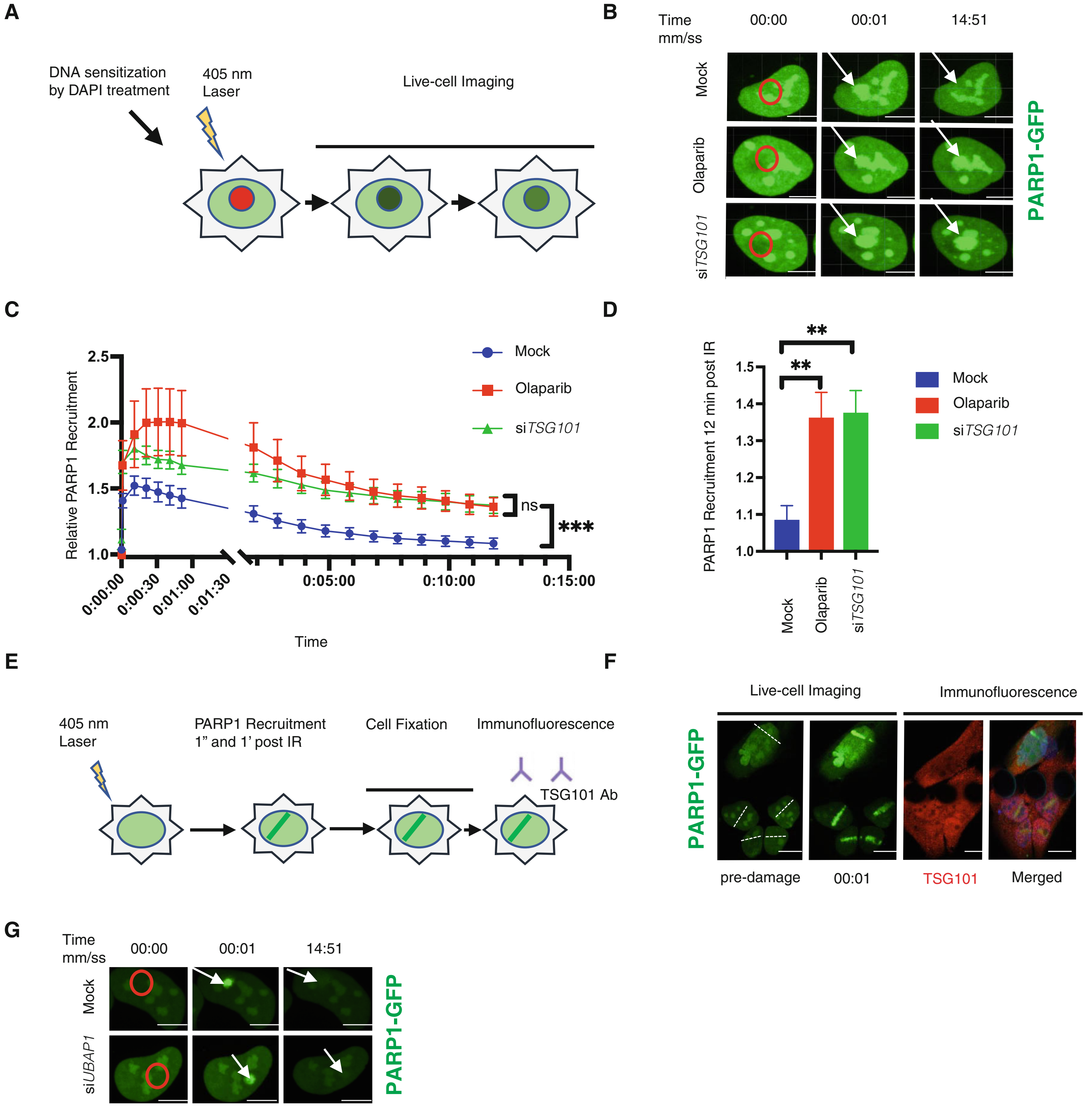

Figure 5.

to investigate the effect of TSG101 loss in the context of *BRCA1/2* deficiency. Strikingly, depletion of TSG101 significantly reduced the viability of *BRCA1* mutant cells (MDA-MB-436), while *BRCA1* wild-type cells (MDA-MB-231) were unaffected (Figs 6G and EV6G). Furthermore, targeting TSG101 affected the cellular integrity of *BRCA1* deficient cells in a similar manner as pharmacological inhibition of PARylation by olaparib (Fong *et al*, 2009) (Fig 6G). Importantly, we found that depletion of TSG101 does not further elevate the killing capacity of pharmacological PARP inhibition in MDA-MB-436 cells (Fig 6H), suggesting an epistatic effect. As a second line of evidence, we analyzed the dependency status of *BRCA1/2*-deficient breast cancer cells to TSG101 expression in the DepMap database (Tsherniak *et al*, 2017). In agreement with our findings, five out of six breast cancer cell lines bearing *BRCA1* mutations showed a strong dependency on TSG101 (Fig 6I and Table EV3), while the sum of breast cancer cell lines was less sensitive to the loss of TSG101

© 2022 The Authors

(Fig 6I), indicating that loss of PARylation in the TSG101 knockout might cause synthetic lethality in these cells. To obtain further evidence, we also targeted *Tsg101* in *Brca1*$^{-/-}$ murine embryonic fibroblasts (MEF) with a CRISPR system. A knockout of *Tsg101* in the *Brca1* null background (Fig EV6E) indeed resulted in severely increased cell death (Fig 6J). Thus, in the context of *BRCA1/2* deficiency, TSG101 has a comparable effect on cell survival as a pharmacological PARP1 inhibition. Taken together, our data demonstrate that TSG101 prevents DNA damage-induced cell death by enabling PARylation and subsequent PARP1-dependent NF-κB pathway activation and plays a crucial role in PARP1-dependent DNA repair processes.

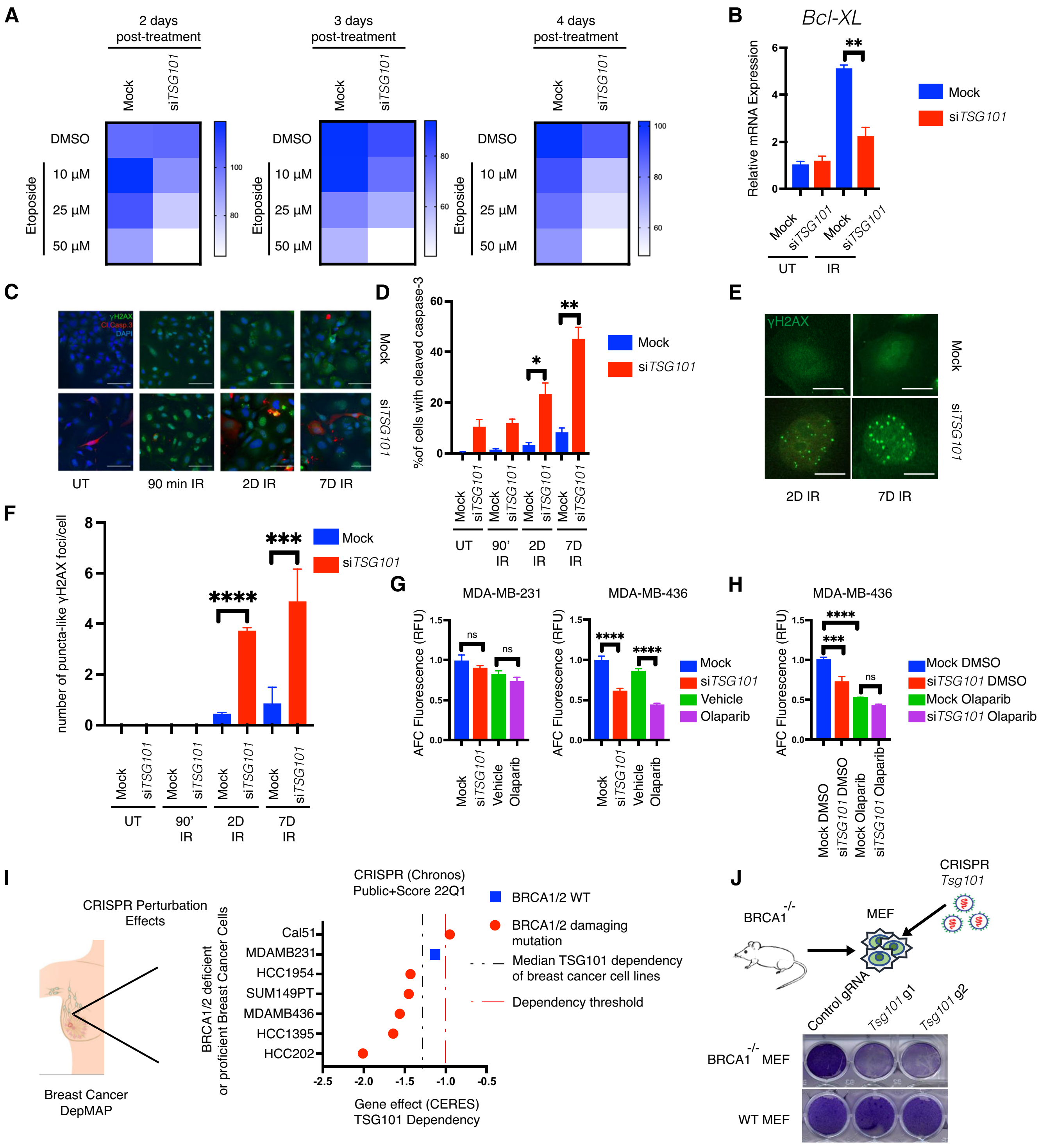

Figure 6.

 © 2022 The Authors

Figure 6. Loss of TSG101 sensitizes cells to apoptosis and impairs DNA repair.

A Survival curve assay. U2-OS cells were transfected with nontargeting or *TSG101*-targeting siRNAs. Cells were treated with increasing concentrations of etoposide (10, 25, and 50 μM). Relative numbers of viable cells in culture were measured using a fluorogenic, cell-permeant peptide substrate (glycyl-phenylalanyl-aminofluorocoumarin; GF-AFC) at 2, 3, or 4 days post-treatment. The data represent five biologically independent experiments. The heatmap displays the relative survival fractions of TSG101-targeted compared with nontargeting siRNA-transfected cells exposed to increasing etoposide concentration at each time point, 2, 3, or 4 days post-treatment, respectively.

B U2-OS cells were transfected with nontargeting (mock) or TSG101-directed siRNAs, kept untreated (UT) or irradiated (20 Gy), and analyzed 1 day later. Total RNA was extracted, and the expression of *BCL2L1* was analyzed by RT–qPCR. *BCL2L1* expression was normalized to the housekeeping genes *ACTA1*, *RPL13A* and *TBP2*.in three biologically independent experiments. The conditions were compared with an unpaired *t*-test (**$P < 0.01$). Error bars represent mean ± SEM.

C Cells were transfected with nontargeting or *TSG101*-targeting siRNAs. Immunofluorescence staining was performed with the indicated antibodies (red: cleaved caspase-3; green: γH2AX). Nuclei were stained with DAPI (blue). The image is a representative of 3 biologically independent experiments. Scale bar: 30 μm. See Fig EV6D for the percentage of γH2AX positive cells as evidence of IR-treatment.

D Percentage of cleaved caspase-3 was determined by blind counting offv approximately 100 cells for each condition from 3 biologically independent experiments. Conditions were compared with an unpaired *t*-test (*$P < 0.05$, **$P < 0.01$). Error bars represent mean ± SEM.

E γH2AX staining of *TSG101*-targeting or nontargeting siRNA-transfected U2-OS cells reveals puncta-like foci at 2 or 7 days after irradiation. Scale bar: 30 μm.

F The number of puncta-like γH2AX foci per cell was determined by blind counting of approximately 100 cells for each condition from 3 biologically independent experiments. Conditions were compared with an unpaired *t*-test (***$P < 0.001$, ****$P < 0.0001$). Error bars represent mean ± SD.

G MDA-MB-231 and MDA-MB-436 cells were transfected with *TSG101*-targeting or nontargeting siRNAs. Cells were treated with vehicle (DMSO) or olaparib (10 μM) 96 h prior to the analysis, as indicated. Results were obtained from five biologically independent experiments. Relative number of viable cells in culture was measured using GF-AFC. See Fig EV6G for brightfield images of cells expressing *TSG101*-targeting or nontargeting siRNAs. Conditions were compared with an unpaired *t*-test (ns, ****$P < 0.0001$). Error bars represent mean ± SD.

H MDA-MB-436 cells were transfected with *TSG101*-targeting or nontargeting (mock) siRNAs. Cells were treated with vehicle (DMSO) or olaparib (10 μM) 96 h prior to the analysis, as indicated. Results were obtained from three biologically independent experiments. Relative number of viable cells in culture was measured using GF-AFC. Conditions were compared with an unpaired *t*-test (ns, ***$P < 0.001$, ****$P < 0.0001$). Error bars represent mean ± SD.

I Analysis of breast cancer cell lines bearing damaging *BRCA1/2* mutations in the DepMap database. The CERES score represents the gene effect, which is based on cell depletion assays of the CRISPR (Avana) public 22Q1 dataset. A CERES score lower than (−1) indicates a high likelihood that the gene of interest is essential in the given cell line. Correspondingly, a − 1 CERES score was indicated with a discontinued red line. The median dependency of all breast cancer cell types to TSG101 expression was indicated with a discontinued black line.

J *BRCA1* deficient MEF cells were lentivirally transduced with two independent guide RNAs targeting mouse *Tsg101* or with empty CRISPR. Cell viability was analyzed with crystal violet staining. CRISPR knockout of *Tsg101* in wild-type MEF cells did not cause cell death. Note that the figure is composed of two separate culture images. The knockout efficiency in wild-type cells was determined by immunoblotting (See Fig EV6E).

Discussion

Genome-wide siRNA screening revealed multiple regulators of the DNA Damage-Induced IKK-NF-κB pathway

NF-κB signaling is important for many physiological processes; however, a genome-wide loss of function screen assessing the role of each particular gene product in the regulation of a specific pathway, to the best of our knowledge, is presented here for the first time. With the advent of high-throughput screening technologies, analysis of the signaling pathway dynamics is now possible, and we applied this approach to systematically identify factors required for the regulation of DNA damage-induced IKK-NF-κB signaling. By combining the screening outcome of genotoxic stress-induced signaling with a counter screening using TNFα, we were not only able to identify DNA damage-selective regulators of the NF-κB pathway but also provide a comprehensive overview of the general regulation of NF-κB signaling. Our dataset underscored previously described key elements of IKK-NF-κB signaling. Importantly, the essential components of the pathway led to abrogated activity in the screen, while targeting negative regulators unleashed the NF-κB pathway (Fig 1C). Previously described negative regulators included TRAF3, which suppresses noncanonical NF-κB signaling by degrading NIK (Sun, 2017), and ESCRT-I components, including VPS28, the depletion of which leads to the accumulation of TNFR receptors in the endosomes, and subsequent activation of NF-κB (Mamińska *et al*, 2016). More essentially, depletion of PARG, which is an enzyme that recycles the PAR chains and attenuates PARylation (Cortes *et al*, 2004), also enhanced the DNA damage-induced NF-κB activation. As expected for the nuclear DNA damage initiated and PARP1-dependent cascade (Dunphy *et al*, 2018), the cGAS-STING pathway did not contribute to NF-κB activation (MB21D1 and TMEM173 in Dataset EV1). Collectively, these results suggest that our screening results represent a rich resource that provides previously unknown protein complexes or biological processes required for or linked to NF-κB signaling, as well as negative regulators that restrict aberrant activation.

DNA damage-induced activation of the IKK-NF-κB pathway is highly dependent on PARylation

In response to DNA damage, a transient PARP1 signalosome complex is formed containing the DNA damage sensors ATM and PARP1 bound to IKKγ and PIASy (Stilmann *et al*, 2009). The interactions between the proteins in this complex are facilitated by PARylation and are requisite for the DNA damage-induced activation of the NF-κB (Stilmann *et al*, 2009). The strong dependence of the activation of the NF-κB pathway on PARylation enabled us to identify components that regulate PARylation. TSG101 is required for PARylation (Figs 3 and EV3). Intriguingly, a recent study revealed that mitochondria-derived NAD^+ is consumed by nuclear PARP1 in response to DNA damage (Hopp *et al*, 2021). Furthermore, a large group of metabolic enzymes (47 hits) was identified in our screening dataset with very high confidence (Table EV2). Investigation of these components will decipher the metabolic networks underlying the NAD homeostasis and NAD-dependent PARylation under genotoxic stress conditions.

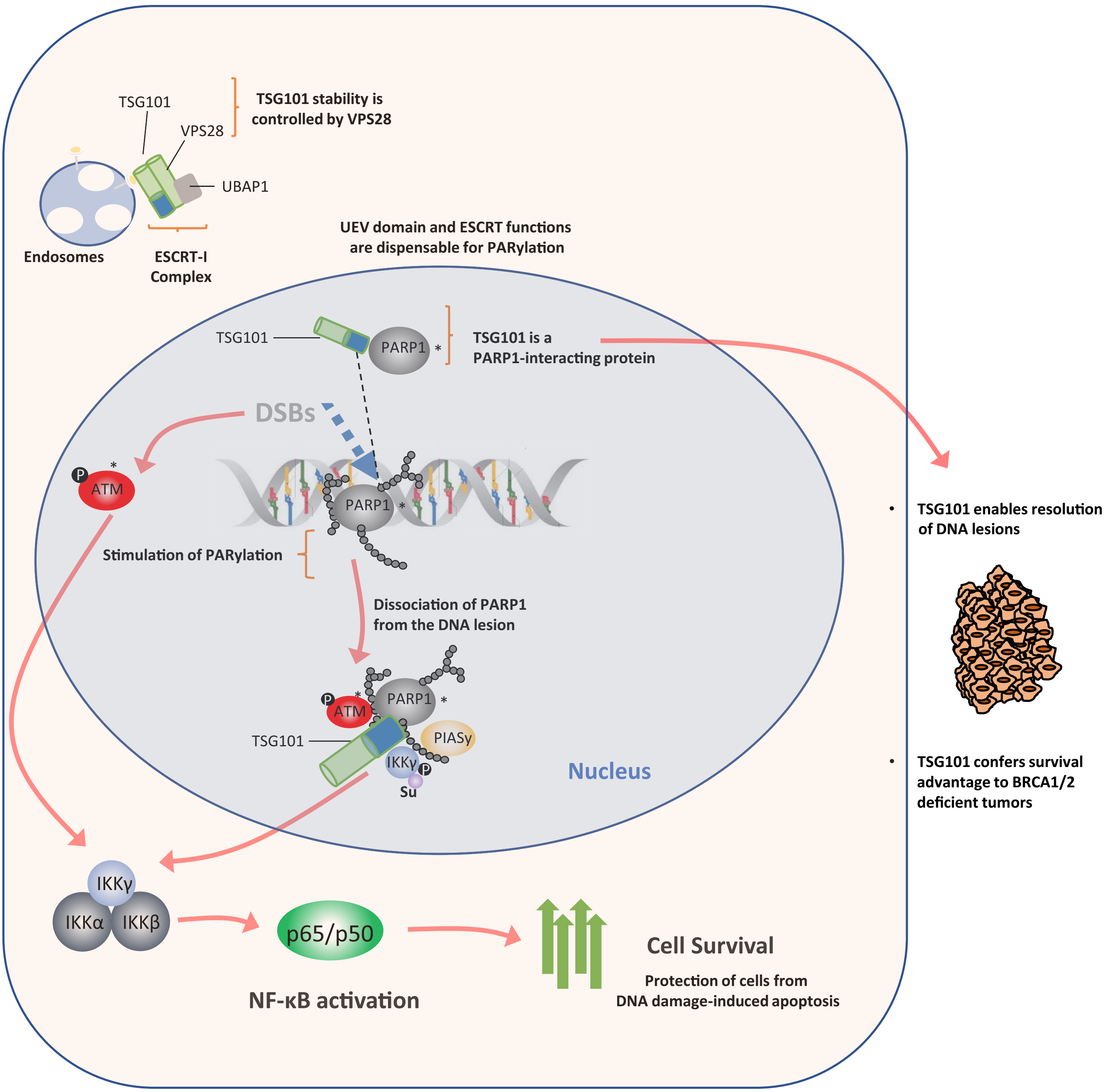

Figure 7. Schematic summary.

Activation of the NF-κB pathway by DNA damage depends on PARylation-dependent formation of a transient nuclear PARP1 signalosome complex with ATM, PIASy, and IKKγ. TSG101 is required for enzymatic PARP1 activity and PAR-dependent DNA damage-induced NF-κB activation. TSG101 interacts with PARP1 and is required for the dissociation of activated PARP1 from DNA lesions. TSG101-dependent PARylation and formation of the TSG101-PARP1 complex are independent of the role of TSG101 in the ESCRT complex. Activation of NF-κB through the TSG101-PARylation axis protects cells from DNA damage-induced apoptosis. TSG101 is also crucial for DNA repair functions and is synthetically lethal in conjunction with BRCA1/2 mutations.

We also provide evidence that TSG101 enables PARylation through protein–protein interaction with PARP1 (Figs 3 and 4). We showed that TSG101 exhibits a robust interaction with PARP1 that is slightly enhanced upon PARP inhibition or DNA damage induction (Fig 4A). In contrast to PARP1, we did not observe an accumulation of TSG101 at the damage sites (Fig 5E). However, TSG101 is present throughout the nucleus and including the DNA lesions and is therefore available for direct interaction with and activation of PARP1.

We have previously identified proteins that associate with increased strength with IKKγ after DNA damage using a SILAC-

 © 2022 The Authors

based IKKγ pull-down (Mikuda *et al*, 2018). These factors included TSG101 and further hits of our genome-wide siRNA screen (Dataset EV5). The increase in TSG101 binding to IKKγ after DNA damage (Mikuda *et al*, 2018) can be explained by the much stronger binding of IKKγ to PARylated PARP1 (Stilmann *et al*, 2009), while TSG101 is drawn into this complex by a PAR-independent association with PARP1 (Fig 4A). We further showed that the CC domain of TSG101 is crucial for PARP1 interaction (Fig 4B) and ectopic overexpression of the CC of TSG101 further enhances IR-induced NADH consumption and PAR formation (Figs 4F and EV4C). In agreement with these observations, TSG101 hyperactivated PARP1 in an *in vitro* system using purified proteins (Fig 4G). Intriguingly, a recent study showed that TSG101 binds with its CC domain to the nuclear glucocorticoid receptor (GR) and folds a disordered region in GR to enhance its transcriptional activity (White *et al*, 2021). Thus, we speculate that a similar scenario may underlie the enzymatic activation of PARP1. Discoveries of PARP1 interaction partners (HPF1 (Gibbs-Seymour *et al*, 2016), CHD1L (Blessing *et al*, 2020), p97 (Krastev *et al*, 2022), TRIP12 (Gatti *et al*, 2020), RNF4 (Martin *et al*, 2009), PARP2 (Schreiber *et al*, 2002), PARP3 (Loseva *et al*, 2010), PARG (Sharifi *et al*, 2013) and CARM1 (Genois *et al*, 2021)) revealed several levels of modulation of the enzymatic activity of PARP1. However, none of these proteins act in a manner functionally equivalent to TSG101, and their depletion, to our knowledge, did not result in complete loss of cellular PARylation. Future determination of the structure of TSG101 bound to PARP1 will reveal how this interaction mediates PARylation and this may facilitate the development of drugs to modulate PARP1 activity.

TSG101 regulates PARylation independently of the ESCRT complex

One major issue targeting TSG101 would be its functions in cellular homeostasis, particularly through the ESCRT system. Our screening results suggested that the role of TSG101 in PARylation is independent of its ESCRT function as TSG101 was the sole ESCRT member that abrogated the DNA damage-induced NF-κB activation (Fig EV5B an Dataset EV1). Importantly, it was previously shown that targeting the ESCRT complex members perturbs TNFR family internalization and upregulates the NF-κB pathway (Mamińska *et al*, 2016). Depletion of VPS28 or UBAP1 led to high superactivation, only of TSG101 to inhibition of NF-κB activity (Dataset EV1). There is an overlap of the two functions for TSG101, activator in the DNA damage pathway and negative regulator endocytosis-related NF-κB activation. To further support the finding that TSG101 controls PARylation independently of the ESCRT complex, we demonstrated that the UEV domain of TSG101, which is responsible for binding to ubiquitinated cargo proteins and mediating the ESCRT-related functions (Vietri *et al*, 2020), does not interact with PARP1 (Fig 4D). Importantly, we showed that TSG101 is found in the nucleus (Fig 5F) at an abundant level, unlike the other ESCRT members, which are localized in the cytoplasm and in endosomes (Thul & Lindskog, 2018), revealing the existence of ESCRT-independent nuclear functions of TSG101. We further demonstrated that another member of the ESCRT complex, UBAP1, does not influence the dissociation of PARP1 from DNA lesions (Fig 5G). Taken together, our data suggest that pharmacological targeting of TSG101-PARP1 complex formation would not interfere with other essential cellular functions of TSG101.

TSG101 is crucial for DNA repair and synthetically lethal with BRCA1/2

Previous attempts to generate a *Tsg101*$^{-/-}$ mouse model revealed that its knockout is embryonically lethal (Ruland *et al*, 2001). Intriguingly, p53 protein accumulation, which was independent of Mdm2 and p53 transcript levels, was observed in the homozygous Tsg101$^{-/-}$ embryos (Ruland *et al*, 2001). This observation further supported our findings that TSG101 is required for PARylation because the increased p53 signaling in Tsg101 null embryos might be a consequence of prolonged loss of PARylation, which is known to accelerate replication fork speed above a tolerated threshold and initiates DNA damage response (Maya-Mendoza *et al*, 2018). Indeed, the requirement of TSG101 for PARylation implicates that TSG101 might be indispensable for DNA repair functions. We showed that in the absence of TSG101, γH2AX foci, likely caused by increased apoptosis, appear days after DNA damage generation as puncta-like large foci (Fig 6E and F). In line with this evidence, we showed that mutant *BRCA1* breast cancer cell types have a strong dependency on TSG101 as demonstrated by the cell viability scores (Fig 6G) and the DepMap database (Tsherniak *et al*, 2017; Fig 6I and Table EV3). TSG101 inactivation and pharmacological PARP inhibition are epistatic (Fig 6H), implicating TSG101 as the equivalent target for the inhibition of PARylation. Taken together, our results (see Fig 7 for a global summary) implicate that pharmacological interference with the TSG101-PARylation signaling axis could exploit synthetic lethality in tumors and impede tumor growth.

Materials and Methods

Materials

Materials, including antibodies, chemicals, peptides, recombinant proteins, commercial assays, recombinant DNA and plasmids, oligonucleotides, and software are listed in Table EV4.

Cell lines

All cell lines were grown under sterile and standard cell culture conditions (humidified atmosphere, 5% CO_2) and routinely tested for mycoplasma.

The NF-κB/293/GFP-Luc™ cell line was cultured with Roswell Park Memorial Institute Medium (RPMI) supplemented with 10% FBS.

A 293T, MEF, and U2-OS cell lines were cultured in high glucose-containing Dulbecco's modified Eagle's medium (DMEM) supplemented with 5% penicillin/streptomycin (P/S) and 10% FBS. The *BRCA1*$^{-/-}$ MEF cells (Callen *et al*, 2013) were kindly provided by Dr. Michela Di Virgilio.

The TSG101 CRISPR knockout bulk cells of U2-OS and MEF origin were cultured in high glucose-containing Dulbecco's modified Eagle's medium (DMEM) supplemented with 5% penicillin/streptomycin (P/S), 10% FBS, and 2 μg/ml puromycin.

© 2022 The Authors

MDA-MB-231 cells were cultured in Dulbecco's modified Eagle's medium (DMEM) supplemented with 5% penicillin/streptomycin (P/S), 10% FBS, and 5% nonessential amino acids. MDA-MB-436 cells were cultured in RPMI 1640 medium supplemented with 5% penicillin/streptomycin and 20% FBS.

High-content siRNA screening

The screening was performed with a genome-wide siRNA library (Ambion Silencer^R Human Genome siRNA library v3, Thermo Fisher), composed of siRNAs targeting approximately 21,000 genes arrayed on 384-well plates. Each screening plate contained several controls such as a nontargeting siRNA (Silencer Negative Control from Ambion), a cell death inducing siRNA mixture, (AllStars Hs Cell Death Control siRNA from Qiagen), and siRNA against IKKγ. For a detailed plate layout, see Fig EV1B.

The NF-κB-driven luciferase activity of the reporter cells (NF-κB/293/GFP-Luc™ cell line) was used as a pathway readout. For the transfection of the siRNA library, on a Freedom EVO 200 workstation (Tecan) 5 µl of a 500 nM library-siRNA-OptiMEM solution was mixed in each well of the 384-well assay plate with 0.05 µl Lipofectamine RNAiMAX transfection reagent previously diluted in 4.95 µl OptiMEM (Life Technologies). Subsequently, 1,000 cells/well in 40 µl antibiotic-free complete medium were seeded onto the predispensed transfection mixture using an EL406™ (BioTek) dispenser resulting in a 50 nM final siRNA concentration in each well.

The screening was performed in duplicate. The first replicate was analyzed in a luciferase assay while the latter was used for high-content imaging to enable visual inspection of the transfected cells. For luciferase measurements, plates were equilibrated for 10 min at room temperature and each well was aspirated to 10 µl volume. A 12.5 µl of ONE-Glo reagent was added to each well and the plate was incubated at room temperature for 30 min. Luminescence was measured using the Safire2 (BioTek) microplate reader with 100 ms integration time. For the high-content imaging, the second replica plate was processed as in the first replicate and fixed using 35 µl/well 4% PFA for 30 min at room temperature. Cells were stained with 10 µM Hoechst for 1 h at room temperature. Plates were sealed and scanned using the ArrayScan™ XTI High Content Platform (Thermo Fisher). In the primary screen, one individual siRNA was transfected per well and measured. The siRNA library used provides three individual siRNAs per target gene, resulting in over 60,000 data points for the approximately 20,000 genes targeted.

Drug treatments

In the screening assays, cells were treated with the indicated agents for 4.5 h (25 µM Etoposide and 10 ng/ml TNFα). For the other experiments, unless otherwise stated, cells were treated with the following agents, at the indicated concentrations, and for the indicated amount of time: Etoposide (50 µM) for 90 min, TNFα (10 ng/ml) for 20 min, or olaparib (10 µM) for 12 h. Irradiation (20 Gy) was applied to cells by using a 137-Cs source (STS OB 29 device) for 45 or 90 min, or at days indicated before experimental analysis.

ONE-GLO™+Tox luciferase reporter assay

The luciferase assays were performed using a ONE-GLO™+Tox kit according to the manufacturer's protocol. In brief, 7,000 cells (NF-κB/293/GFP-Luc™) were seeded into 96-well plates with a black bottom and walls using a 100 µl growth medium RPMI supplemented with 5% penicillin/streptomycin (P/S) and 10% FBS, treated and transfected with indicated siRNAs if needed. The GF-FC dye was mixed in TOX substrate buffer in a 1:200 ratio and 20 µl of this solution was added to each well and incubated for 30 min at 37°C. The TOX cell viability signal was measured using a plate reader (Cytation 1) with 380 nm (Excitation) and 500 nm (Emission) filter sets. Subsequently, 100 µl of the luciferase substrate was added to each well and incubated for 3 min at RT. The luminescence signal was measured with the plate reader and normalized to the respective viability signal (TOX).

siRNA and plasmid transfections

The transient knockdown of indicated genes was performed using Lipofectamine RNAiMAX according to the manufacturer's protocols. In brief, the day after seeding cells into a 6 cm dish, at 2.5×10^5 cells per dish quantity, 25 nM siRNA was mixed with 5 µl lipofectamine in 500 µl serum-free OptiMEM and incubated for 5 min at RT before adding to the cells in a dropwise manner. The downstream experiments were performed 3 days after the transfection. A scrambled siRNA was used as a control for each experiment.

Ectopic expression of indicated recombinant DNA for the live-cell imaging experiments was performed using Lipofectamine 2000 according to the manufacturer's protocols. In brief, the day after seeding cells into µ-Slide 8 well chamber (Ibidi), at 2.5×10^4 cells per dish quantity, 1.5 µg recombinant DNA was mixed with 3 µl of lipofectamine in 50 µl serum-free OptiMEM and incubated for 5 min at RT before adding to the cells in a dropwise manner. For immunoprecipitation experiments, the indicated recombinant DNAs were transfected to the cells using polyethyleneimine (PEI). Cells were grown in 15 cm dishes at roughly 70% confluency, 15 µg of recombinant DNA was mixed with 90 µl of PEI in 2.85 ml serum and antibiotic-free DMEM and incubated for 30 min at RT before adding the cells. The downstream experiments were performed 2 days after the transfection.

Immunoprecipitation

Immunoprecipitation of proteins was performed as previously described (Mikuda *et al*, 2018). In brief, cells were harvested in an ice-cold CHAPS buffer (Tris–HCl pH 7.4, 110 mM NaCl, and 50 mM EDTA, supplemented with 1% (v/v) CHAPS, complete protease and phosphatase inhibitors cocktails), incubated with primary antibodies for overnight at 4°C, with G Fast Flow Sepharose Beads for 1 h at 4°C. Samples were boiled and subjected to standard SDS–PAGE.

Immunoprecipitation of GFP-tagged proteins was performed using GFP-trap (Chromotek) according to the manufacturer's instructions. Briefly, cells were harvested in ice-cold RIPA buffer. Volume-matched 1 µg lysates were incubated with the bead slurry by end-over-end rotation for 1 h at 4 °C. The beads were then sedimented and washed 3 times with RIPA buffer. After the last washing step, samples were mixed with 2X SDS-sample buffer and boiled.

 © 2022 The Authors

Beads were then sedimented again and the supernatants were subjected to standard SDS–PAGE. Immunoprecipitation of Flag-tagged proteins was performed using anti-Flag M2 agarose beads (Sigma) according to the manufacturer's instructions. Briefly, cells were harvested using Triton X-100 lysis buffer (50 mM Tris–HCl, pH 7.4, 150 mM NaCl, 1 mM EDTA, 1% Triton X-100). Volume-matched 1.5 μg lysate proteins were incubated with the bead slurry by end-over-end rotation overnight at 4°C. The beads were then sedimented and washed 3 times with TBS buffer. After the last washing step, samples were mixed with 2X SDS-sample buffer and boiled. Beads were then sedimented once again and the supernatants were analyzed in standard SDS–PAGE.

Protein purification

A 293T cells were transfected with GFP-TSG101 plasmid using PEI. Cells were harvested in ice-cold RIPA buffer 48 h after transfection. GFP-TSG101 was immunoprecipitated using the GFP-trap method. To dissociate TSG101 from its binding partners, beads were additionally washed with high-salt (2M NaCl) and high detergent (1% Triton™ X-100) containing wash buffers separately. Each washing step was repeated 3 times. Proteins were eluted from the beads using an acidic elution buffer at room temperature. Solution was immediately neutralized with a neutralization buffer prior to storage.

In vitro ADP-ribosylation assay

In vitro ADP ribosylation was performed as previously described (Di Giammartino *et al*, 2013). The indicated amounts of TSG101 and PARP1 were incubated in buffer D (dialysis buffer) with 1 mM NAD^+, 400 ng sss (sheared salmon sperm) DNA, and 10 mM $MgCl_2$. The reaction was carried out at 37°C for 10 min and stopped by adding 2X SDS-loading buffer. After boiling, samples were subjected to standard SDS–PAGE.

Total NAD and NADH measurements

Total NAD and NADH measurements were performed using a colorimetric NAD/NADH assay kit according to the manufacturer's instructions. Briefly, 2.5×10^5 cells were used for each condition. Cells were washed and scraped using ice-cold PBS. Cell pellets were mixed with NADH extraction buffer and the lysates were obtained by repeated freezing and thawing cycles. To remove any NADH-consuming enzymes, lysates were filtered. For the detection of NADH, lysates were incubated at 60°C for 30 min. Under these conditions, all NAD^+ will be decomposed while the NADH will still be intact. Samples were then cooled on ice. The NAD decomposed samples were converted to NADH using NAD Cycling Enzyme Mix. NADH developer was then added to the reaction and OD 450 nm reads were obtained using a plate reader. Total NAD was quantified as in NADH measurement and NAD^+ was calculated by subtracting NADH from the total NAD value.

Cleaved Caspase-3 measurement (colorimetric)

Enzymatic activity of the cleaved caspase-3 was measured using a Caspase-3 Assay Kit (Colorimetric) (Abcam, ab39401), according to the manufacturer's instructions. Briefly, cells were harvested in PBS and cytoplasmic extracts were obtained. Protein concentrations were determined by a Bradford Assay and equal quantities of protein (100 μg for each condition) were tested in a colorimetric assay. Cleaved caspase-3 activity was measured using a plate reader. Values were subtracted from only buffer-containing reaction and normalized to the level of nontargeting CRISPR transduced cells.

CellTiter-Fluor™ cell viability assay

Cell viability was assayed using the CellTiter-Fluor™ kit (Promega, G6081) according to the manufacturer's instructions. Briefly, cells were seeded onto 96-well plates with white walls and bottom at 10^5 cells/well density. Indicated treatments and transfections were all performed in that plate. For cell viability measurements, GF-AFC substrates were added to each well. Cells were incubated at 37°C for 30 min and cell viability was measured using a plate reader. After subtracting the measurement from the reaction containing only medium, the values were normalized to the level of control siRNA-transfected cells.

Western blotting

For western blots, cell pellets were lysed in whole-cell extraction buffer (150 mM NaCl, 50 mM Tris–pH 7.5, 1% NP-40 supplemented with proteinase and phosphatase inhibitors) for 20 min at 4°C while vigorously shaking, insoluble material was spun out, relative protein concentration was determined by BCA (BioRad). SDS-sample buffer with 100 mM DTT was added to samples, which were loaded onto 8% Protein gels and separated by electrophoresis at 90–140 V for 2–3 h. Proteins were transferred and immobilized onto a nitrocellulose membrane (GE Healthcare) by electrophoresis for 2 h at RT in a standard transfer buffer containing 20% methanol. Membranes were blocked in 5% nonfat dry milk in TBS-T. All rabbit primary antibodies were probed with Peroxidase AffiniPure Donkey anti-rabbit IgG (Jackson ImmunoResearch) and all mouse primary antibodies were probed with Peroxidase AffiniPure Donkey anti-mouse IgG (Jackson ImmunoResearch). Chemiluminescent signals were visualized with Fusion Solo Imager.

Proximity ligation assay (PLA)

The DuoLink (Merck) PLA assay was performed according to the manufacturer's protocols. Cells were seeded in 12 mm glass coverslips 48 h before the experiment and treated if needed, fixed with 4% PFA at RT for 10 min, and permeabilized for 10 min at RT in PBS supplemented with 0.1% Triton X-100 (Sigma Aldrich). For blocking, cells were incubated for 1 h at 37°C in the provided blocking solution. Incubation with primary antibodies (PARP1, Thermo Fisher, Cat#MA 3-950, TSG101, Thermo Fisher, Cat#MA 1-23296, both diluted 1:200) was done overnight at 4°C.

Laser microirradiation and live-cell imaging

Cells were grown in a μ-Slide 8-well chamber (Ibidi) and transiently transfected with GFP-tagged PARP1 and imaged at 24 h

© 2022 The Authors

post-transfection. Throughout the experiment, cells were maintained in Dulbecco's modified Eagle's medium (DMEM) supplemented with and 10% FBS, at 37°C with 5% CO_2. For sensitization, cells were incubated with a DNA damage sensitizer DAPI (10 µg/ml) for 10 min before the microirradiation. Cells with low to moderate GFP intensities were selected for the experiments. Targeted laser microirradition was induced by a 405 nm laser (100 mW) using FRAPPA of the confocal spinning disk microscope (Andor CSU-W1 with Borealis on a Nikon Ti Eclipse microscope equipped with an iXON DU888 EMCCD camera). The laser power output was frequently measured to ensure a constant DNA damage effect. A preselected area in the nucleus (a round-shaped with approximately 130-µm diameter) was microirradiated using a 100X CFI PLAN-Apo NA 1.4 oil objective. The stimulation was performed using 25% laser power for 500 µs dwell time. In preliminary experiments, the dosage of 405 nm laser light was adjusted to the lowest possible amount of laser needed to induce DNA damage without compromising cell viability. Subsequent image acquisition was performed using a 488 nm laser line with the same objective and a BP525/45 emission filter. Z-stacks of 20 slices were selected toward their top and bottom ends. The recruitment and dissociation of PARP1 to and from the DNA damage sites were followed with the time series up to 15 min with the first time point immediately after the microirradiation event.

For endogenous protein recruitment assays, cells were grown in a µ-Slide 8-well-gridded chamber (Ibidi). The sensitization and transfection of the cells were done as in the live-cell imaging. To facilitate easy recognition of laser microirradiated cells during the imaging of endogenous TSG101, the DNA damage sites were created on a straight line in the nucleus of the selected cells, but the parameters (405 nm laser wavelength with 25% laser power and 500 µs Dwell time) were kept consistent. Recruitment of PARP1 to the DNA damage site was recorded for either 1 or 5 min and cells were fixed with PFA and stained for TSG101 with standard indirect immunofluorescence. The microirradiated cells were refound and imaged with the confocal microscope (CSU Yokogawa Spinning Disk Field Scanning Confocal System, Nikon).

Indirect immunofluorescence

For all IF experiments (except the endogenous recruitment assay), cells were grown on 12 mm glass coverslips, treated if required, fixed in 4% PFA for 10 min at RT, and permeabilized for 10 min at RT in PBS supplemented with 0.1% Triton X-100 (Sigma Aldrich). Cells were blocked in filtered PBS supplemented with 0.1% Triton X-100 and 5% BSA for 1 h. The primary antibodies were diluted in the blocking buffer and cells were incubated with the antibody solution overnight at 4°C. All secondary antibodies were diluted in the blocking buffer and incubated for 1 h at RT. After each antibody incubation cells were washed 3 times with PBS. Following the last washing, coverslips were mounted with ProLong™ Gold Antifade Mountant with DAPI. The primary antibodies used for IF at the indicated concentrations were against cleaved caspase-3 (Cell Signaling, 1:400), p65 (Cell Signaling, 1:500), PARP1 (Cell Signaling, 1:1,000), and TSG101 (Thermo Fisher, 1:500). See Key Resources Table for the secondary antibodies and the fluorophores. Confocal immunofluorescence microscopy was done as previously described (Mikuda *et al*, 2018; Kolesnichenko *et al*, 2021).

qRT–PCR

qRT–PCR was performed as previously described (Mikuda *et al*, 2018). In brief, primers were designed according to MIQE guidelines (Minimum Information for Publication of qRT–PCR Experiments) using an NCBI primer blast, and 62°C melting temperature was selected. To determine the fold induction of target genes, two to three reference genes (*ACTA1*, *RPL13A*, and *TBP2*) with an m value lower than 0.5 were used. The indicated primers were obtained from previous studies (von Hoff *et al*, 2019; Mikuda *et al*, 2020; Kolesnichenko *et al*, 2021). See Key Resources Table for qRT–PCR primers for details.

Nuclear and cytoplasmic fractionation

The subcellular fractionation was performed as previously described (Mikuda *et al*, 2018). In brief, cells were washed with ice-cold PBS and harvested by scraping in 500 µl buffer A (10 mM Tris–HCl (pH 7.9, 1.5 mM $MgCl_2$, 10 mM KCl, supplemented with complete protease and phosphatase inhibitors)). After incubation on ice, NP-40 was added to a final concentration of 0.15% (v/v). Samples were washed and centrifuged, and supernatants containing the cytoplasmic fraction were collected. The remaining pellet was further washed with buffer A containing NP-40 and resuspended with two volumes of buffer C (20 mM Tris–HCl pH 7.9, 25% glycerol, 0.42 M NaCl, 1.5 mM $MgCl_2$, 0.5% NP-40, supplemented with complete protease and phosphatase inhibitors). The insoluble nuclear debris was pelleted by centrifugation and nuclear lysates were subsequently collected.

Electrophoretic mobility shift assay (EMSA)

Nuclear extracts were used for the EMSA experiments. The assay was performed as previously described (Hinz *et al*, 2010). In brief, 5 µg of nuclear extract was mixed with radioactively labeled (P^{32}) H2K probes supplemented with shift buffer (40 mM HEPES pH 7.9, 120 mM KCl, and 8% (v/v) Ficoll), 2 µg/ml poly dI/dC, 100 mM DTT and 10 µg/ml BSA and incubated for 30 min at RT. Samples were separated by electrophoresis and transferred to Whatman filter paper by vacuum dryer. The dried gel was incubated with Hyperfilm™ MP at −80°C overnight and developed with standard procedures.

CRISPR-Cas9 knockout bulk cell generation

CRISPR-Cas9-mediated knockout experiments were performed as previously described (Ran *et al*, 2013). In brief, the indicated guide RNA sequences were annealed and subcloned into lentiCRISPRv2 (Addgene, Cat#52961). The cloning was verified by Sanger sequencing. For lentiviral production, 293T cells were seeded into 15 cm plates at 90% confluency 1 day before transfection. The lentiCRISPRv2 vectors (30 µg) containing respective guide RNAs were transfected to the cells together with 20 µg of psPAX2 (Addgene, Cat#8454) and 10 µg of pCMV-VSV-G (Addgene, Cat#12260) using PEI. Lentiviruses were harvested and concentrated with PEG Virus Precipitation Kit (Abcam, Cat#ab102538) according to the manufacturer's protocol. The target cells were seeded into 24-well plates at 20–40% confluency and lentivirally transduced with the

© 2022 The Authors

concentrated viruses at a 1:25 ratio in antibiotic-free DMEM supplemented with 10 μg/ml Polybrene (Sigma Aldrich). For the elimination of untransduced cells, puromycin (2 μg/ml) was added to the growth medium 3 days after the transduction. Knockout efficiencies were determined with western blotting. Due to the conclusive roles of TSG101 in cell division and development, a double knockout cell line was not established after single-cell expansion experiments.

Crystal violet staining

Cells were seeded into 6-well plates, transfected, and treated if needed. The growth medium was gently aspirated and cells were fixed in ice-cold methanol for 10 min at 4°C. The plate was equilibrated to RT and incubated with crystal violet staining solution for 10 min at RT. The staining solution was removed and cells were washed with water. Crystal violet intensities were measured at 570 nm with a plate reader (Cytation 1, Biotek).

Analysis of siRNA screening data

The statistical effect size (Z-factor) was calculated for each plate (189 in total) using the mock-transfected vehicle (DMSO) or etoposide (Sigma) treated cells with negative and positive control, respectively. The Z-factor cut-off was 0.1, 107 plates have a Z-factor below 0.5, 82 plates have a Z-factor equal to or larger than 0.5, and the mean Z-Prime is 0.45. Several plates were repeated to improve quality. See Dataset EV2 for detailed Z-factor determination.

To determine assay reproducibility of the screening, 12 plates from the Druggable Genome Library were randomly selected for a replicate experiment. Results obtained from both replicates were compared and their correlation was determined by Spearman's rank correlation coefficiency test (Table EV1).

For plate-wise normalization, the data obtained in the genome-wide screen were processed on the KNIME Analytics Platform (KNIME). A Z-score was calculated from luminescence values as follows: *x.zscore = (x − median(x[subset]))/mad(x[subset])*, where x is the luminescence value and the subset are all sample wells in the plate.

For the gene set enrichment analysis of the genome-wide screening hits, the REACTOME database (Jassal *et al*, 2020) was used.

Analysis of quantitative live-cell imaging

Imaris software (Oxford Instruments) was used to quantify the recruitment and dissociation kinetics of PARP1 to and from the DNA lesions. Images of pre- and post-microirradiated cells were combined and the laser microirradiated area was segmented as a region of interest (ROI) in Imaris. The mean fluorescence intensity of the ROI was acquired for each time point. The fluorescence intensity of the whole nucleus was also acquired as a reference ROI and fluorescence intensity at the DNA damage site was normalized to the respective reference ROI.

Statistical tests

For statistical analysis, the normalized mean fluorescence intensities from 9 independent experiments were compared using an ordinary one-way ANOVA (ns, $P > 0.05$; *$P < 0.05$; **$P < 0.01$, ***$P < 0.001$; ****$P < 0.0001$).

Data availability

The raw luciferase assay data from the genome-wide siRNA screen can be accessed using the following DOI: 10.5281/zenodo.6805593 (Data ref: Tufan *et al*, 2022).

Expanded View for this article is available online.

Acknowledgements

We thank Dr. Michela Di Virgilio for discussion and Brca1$^{-/-}$ MEF cells. We thank Dr. Ivan Ahel for providing Flag-tagged PARP1 and PARP2 plasmids (Gibbs-Seymour *et al*, 2016). A.B.T. acknowledges DFG, GSC 1091, Berlin Graduate School for Integrative Oncology (B.S.I.O.), and Deutscher Akademischer Austauschdienst (DAAD) for support. The work was funded in part by DFG grant KO6390/1-1 to M.K. and in part by grants from BMBF, CancerSys project ProSiTu and Helmholtz Association iMed and PreGo-Bio and European Regional Development Fund (EFRE), State of Berlin, Germany, to C.S. Open Access funding enabled and organized by Projekt DEAL.

Author contributions

Ahmet Buğra Tufan: Data curation; formal analysis; validation; investigation; methodology; writing – original draft; writing – review and editing. **Katina Lazarow:** Resources; data curation; formal analysis; validation; investigation; methodology. **Marina Kolesnichenko:** Data curation; formal analysis; validation; investigation. **Anje Sporbert:** Data curation; formal analysis; validation; investigation; visualization; methodology. **Jens Peter von Kries:** Conceptualization; resources; data curation; supervision; methodology. **Claus Scheidereit:** Conceptualization; supervision; funding acquisition; investigation; writing – original draft; project administration; writing – review and editing.

Disclosure and competing interests statement

The authors declare that they have no conflict of interest.

References

Ahel D, Horejsí Z, Wiechens N, Polo SE, Garcia-Wilson E, Ahel I, Flynn H, Skehel M, West SC, Jackson SP *et al* (2009) Poly(ADP-ribose)-dependent regulation of DNA repair by the chromatin remodeling enzyme ALC1. *Science* 325: 1240 – 1243

Arnesen T, Anderson D, Baldersheim C, Lanotte M, Varhaug JE, Lillehaug JR (2005) Identification and characterization of the human ARD1-NATH protein acetyltransferase complex. *Biochem J* 386: 433 – 443

Babst M, Odorizzi G, Estepa EJ, Emr SD (2000) Mammalian tumor susceptibility gene 101 (TSG101) and the yeast homologue, Vps23p, both function in late endosomal trafficking. *Traffic* 1: 248 – 258

Blessing C, Mandemaker IK, Gonzalez-Leal C, Preisser J, Schomburg A, Ladurner AG (2020) The oncogenic helicase ALC1 regulates PARP inhibitor potency by trapping PARP2 at DNA breaks. *Mol Cell* 80: 862 – 875.e6

Bonner WM, Redon CE, Dickey JS, Nakamura AJ, Sedelnikova OA, Solier S, Pommier Y (2008) GammaH2AX and cancer. *Nat Rev Cancer* 8: 957 – 967

Callen E, Di Virgilio M, Kruhlak MJ, Nieto-Soler M, Wong N, Chen H-T, Faryabi RB, Polato F, Santos M, Starnes LM *et al* (2013) 53BP1 mediates productive

and mutagenic DNA repair through distinct phosphoprotein interactions. *Cell* 153: 1266 – 1280

Colomer C, Margalef P, Villanueva A, Vert A, Pecharroman I, Solé L, González-Farré M, Alonso J, Montagut C, Martinez-Iniesta M *et al* (2019) IKKα kinase regulates the DNA damage response and drives chemo-resistance in cancer. *Mol Cell* 75: 669 – 682.e5

Cortes U, Tong W-M, Coyle DL, Meyer-Ficca ML, Meyer RG, Petrilli V, Herceg Z, Jacobson EL, Jacobson MK, Wang Z-Q (2004) Depletion of the 110-kilodalton isoform of poly(ADP-ribose) glycohydrolase increases sensitivity to genotoxic and endotoxic stress in mice. *Mol Cell Biol* 24: 7163 – 7178

D'Amours D, Desnoyers S, D'Silva I, Poirier GG (1999) Poly(ADP-ribosyl)ation reactions in the regulation of nuclear functions. *Biochem J* 342: 249 – 268

Di Giammartino DC, Shi Y, Manley JL (2013) PARP1 represses PAP and inhibits polyadenylation during heat shock. *Mol Cell* 49: 7 – 17

Doyotte A, Russell MRG, Hopkins CR, Woodman PG (2005) Depletion of TSG101 forms a mammalian 'class E' compartment: a multicisternal early endosome with multiple sorting defects. *J Cell Sci* 118: 3003 – 3017

Dunphy G, Flannery SM, Almine JF, Connolly DJ, Paulus C, Jønsson KL, Jakobsen MR, Nevels MM, Bowie AG, Unterholzner L (2018) Non-canonical activation of the DNA sensing adaptor STING by ATM and IFI16 mediates NF-κB signaling after nuclear DNA damage. *Mol Cell* 71: 745 – 760.e5

Ferraiuolo R-M, Manthey KC, Stanton MJ, Triplett AA, Wagner K-U (2020) The multifaceted roles of the tumor susceptibility gene 101 (TSG101) in normal development and disease. *Cancers* 12: 450

Fong PC, Boss DS, Yap TA, Tutt A, Wu P, Mergui-Roelvink M, Mortimer P, Swaisland H, Lau A, O'Connor MJ *et al* (2009) Inhibition of poly(ADP-ribose) polymerase in tumors from BRCA mutation carriers. *N Engl J Med* 361: 123 – 134

Fu J, Huang D, Yuan F, Xie N, Li Q, Sun X, Zhou X, Li G, Tong T, Zhang Y (2018) TRAF-interacting protein with forkhead-associated domain (TIFA) transduces DNA damage–induced activation of NF-κB. *J Biol Chem* 293: 7268 – 7280

Gatti M, Imhof R, Huang Q, Baudis M, Altmeyer M (2020) The ubiquitin ligase TRIP12 limits PARP1 trapping and constrains PARP inhibitor efficiency. *Cell Rep* 32: 107985

Genois M-M, Gagné J-P, Yasuhara T, Jackson J, Saxena S, Langelier M-F, Ahel I, Bedford MT, Pascal JM, Vindigni A *et al* (2021) CARM1 regulates replication fork speed and stress response by stimulating PARP1. *Mol Cell* 81: 784 – 800.e8

Gibbs-Seymour I, Fontana P, Rack JGM, Ahel I (2016) HPF1/C4orf27 is a PARP-1-interacting protein that regulates PARP-1 ADP-ribosylation activity. *Mol Cell* 62: 432 – 442

Gibson BA, Kraus WL (2012) New insights into the molecular and cellular functions of poly(ADP-ribose) and PARPs. *Nat Rev Mol Cell Biol* 13: 411 – 424

Goldstein M, Kastan MB (2015) The DNA damage response: implications for tumor responses to radiation and chemotherapy. *Annu Rev Med* 66: 129 – 143

Haskill S, Beg AA, Tompkins SM, Morris JS, Yurochko AD, Sampson-Johannes A, Mondal K, Ralph P, Baldwin AS (1991) Characterization of an immediate-early gene induced in adherent monocytes that encodes I kappa B-like activity. *Cell* 65: 1281 – 1289

Helleday T (2011) The underlying mechanism for the PARP and BRCA synthetic lethality: clearing up the misunderstandings. *Mol Oncol* 5: 387 – 393

Hinz M, Stilmann M, Arslan SÇ, Khanna KK, Dittmar G, Scheidereit C (2010) A cytoplasmic ATM-TRAF6-cIAP1 module links nuclear DNA damage signaling to ubiquitin-mediated NF-κB activation. *Mol Cell* 40: 63 – 74

von Hoff L, Kärgel E, Franke V, McShane E, Schulz-Beiss KW, Patone G, Schleussner N, Kolesnichenko M, Hübner N, Daumke O *et al* (2019) Autocrine LTA signaling drives NF-κB and JAK-STAT activity and myeloid gene expression in Hodgkin lymphoma. *Blood* 133: 1489 – 1494

Hopp A-K, Teloni F, Bisceglie L, Gondrand C, Raith F, Nowak K, Muskalla L, Howald A, Pedrioli PGA, Johnsson K *et al* (2021) Mitochondrial NAD+ controls nuclear ARTD1-induced ADP-ribosylation. *Mol Cell* 81: 340 – 354.e5

Huang S, Pettaway CA, Uehara H, Bucana CD, Fidler IJ (2001) Blockade of NF-kappaB activity in human prostate cancer cells is associated with suppression of angiogenesis, invasion, and metastasis. *Oncogene* 20: 4188 – 4197

Jackson SP, Bartek J (2009) The DNA-damage response in human biology and disease. *Nature* 461: 1071 – 1078

Jassal B, Matthews L, Viteri G, Gong C, Lorente P, Fabregat A, Sidiropoulos K, Cook J, Gillespie M, Haw R *et al* (2020) The reactome pathway knowledgebase. *Nucleic Acids Res* 48: D498 – D503

Jungmichel S, Rosenthal F, Altmeyer M, Lukas J, Hottiger MO, Nielsen ML (2013) Proteome-wide identification of poly(ADP-ribosyl)ation targets in different genotoxic stress responses. *Mol Cell* 52: 272 – 285

Kim MY, Zhang T, Kraus WL (2005) Poly(ADP-ribosyl)ation by PARP-1: 'PAR-laying' NAD+ into a nuclear signal. *Genes Dev* 19: 1951 – 1967

Kolesnichenko M, Mikuda N, Höpken UE, Kärgel E, Uyar B, Tufan AB, Milanovic M, Sun W, Krahn I, Schleich K *et al* (2021) Transcriptional repression of NFKBIA triggers constitutive IKK- and proteasome-independent p65/RelA activation in senescence. *EMBO J* 40: e104296

Kolmus K, Erdenebat P, Szymańska E, Stewig B, Goryca K, Derezińska-Wołek E, Szumera-Ciećkiewicz A, Brewińska-Olchowik M, Piwocka K, Prochorec-Sobieszek M *et al* (2021) Concurrent depletion of Vps37 proteins evokes ESCRT-I destabilization and profound cellular stress responses. *J Cell Sci* 134: jcs250951

Krastev DB, Li S, Sun Y, Wicks AJ, Hoslett G, Weekes D, Badder LM, Knight EG, Marlow R, Pardo MC *et al* (2022) The ubiquitin-dependent ATPase p97 removes cytotoxic trapped PARP1 from chromatin. *Nat Cell Biol* 24: 62 – 73

Krishnakumar R, Kraus WL (2010) The PARP side of the nucleus: molecular actions, physiological outcomes, and clinical targets. *Mol Cell* 39: 8 – 24

Kuleshov MV, Jones MR, Rouillard AD, Fernandez NF, Duan Q, Wang Z, Koplev S, Jenkins SL, Jagodnik KM, Lachmann A *et al* (2016) Enrichr: a comprehensive gene set enrichment analysis web server 2016 update. *Nucleic Acids Res* 44: W90 – W97

Lee MH, Mabb AM, Gill GB, Yeh ETH, Miyamoto S (2011) NF-κB induction of the SUMO protease SENP2: a negative feedback loop to attenuate cell survival response to genotoxic stress. *Mol Cell* 43: 180 – 191

Li N, Banin S, Ouyang H, Li GC, Courtois G, Shiloh Y, Karin M, Rotman G (2001) ATM is required for IkappaB kinase (IKKk) activation in response to DNA double strand breaks. *J Biol Chem* 276: 8898 – 8903

Loseva O, Jemth A-S, Bryant HE, Schüler H, Lehtiö L, Karlberg T, Helleday T (2010) PARP-3 is a mono-ADP-ribosylase that activates PARP-1 in the absence of DNA. *J Biol Chem* 285: 8054 – 8060

Luo X, Kraus WL (2012) On PAR with PARP: Cellular stress signaling through poly(ADP-ribose) and PARP-1. *Genes Dev* 26: 417 – 432

Mabb AM, Wuerzberger-Davis SM, Miyamoto S (2006) PIASy mediates NEMO sumoylation and NF-κB activation in response to genotoxic stress. *Nat Cell Biol* 8: 986 – 993

Mamińska A, Bartosik A, Banach-Orłowska M, Pilecka I, Jastrzębski K, Zdżalik-Bielecka D, Castanon I, Poulain M, Neyen C, Wolińska-Nizioł L *et al* (2016) ESCRT proteins restrict constitutive NF-κB signaling by trafficking cytokine receptors. *Sci Signal* 9: ra8

 © 2022 The Authors

Martin N, Schwamborn K, Schreiber V, Werner A, Guillier C, Zhang X-D, Bischof O, Seeler J-S, Dejean A (2009) PARP-1 transcriptional activity is regulated by sumoylation upon heat shock. *EMBO J* 28: 3534 – 3548

Maya-Mendoza A, Moudry P, Merchut-Maya JM, Lee M, Strauss R, Bartek J (2018) High speed of fork progression induces DNA replication stress and genomic instability. *Nature* 559: 279 – 284

Mikuda N, Kolesnichenko M, Beaudette P, Popp O, Uyar B, Sun W, Tufan AB, Perder B, Akalin A, Chen W *et al* (2018) The IκB kinase complex is a regulator of mRNA stability. *EMBO J* 37: e98658

Mikuda N, Schmidt-Ullrich R, Kärgel E, Golusda L, Wolf J, Höpken UE, Scheidereit C, Kühl AA, Kolesnichenko M (2020) Deficiency in IκBα in the intestinal epithelium leads to spontaneous inflammation and mediates apoptosis in the gut. *J Pathol* 251: 160 – 174

Mortusewicz O, Amé J-C, Schreiber V, Leonhardt H (2007) Feedback-regulated poly(ADP-ribosyl)ation by PARP-1 is required for rapid response to DNA damage in living cells. *Nucleic Acids Res* 35: 7665 – 7675

Piret B, Schoonbroodt S, Piette J (1999) The ATM protein is required for sustained activation of NF-kappaB following DNA damage. *Oncogene* 18: 2261 – 2271

Polo SE, Jackson SP (2011) Dynamics of DNA damage response proteins at DNA breaks: A focus on protein modifications. *Genes Dev* 25: 409 – 433

Ran FA, Hsu PD, Wright J, Agarwala V, Scott DA, Zhang F (2013) Genome engineering using the CRISPR-Cas9 system. *Nat Protoc* 8: 2281 – 2308

Ruland J, Sirard C, Elia A, MacPherson D, Wakeham A, Li L, Luis de la Pompa J, Cohen SN, Mak TW (2001) p53 accumulation, defective cell proliferation, and early embryonic lethality in mice lacking tsg101. *Proc Natl Acad Sci U S A* 98: 1859 – 1864

Schreiber V, Amé J-C, Dollé P, Schultz I, Rinaldi B, Fraulob V, Ménissier-de Murcia J, de Murcia G (2002) Poly(ADP-ribose) polymerase-2 (PARP-2) is required for efficient base excision DNA repair in association with PARP-1 and XRCC1. *J Biol Chem* 277: 23028 – 23036

Shao L, Zhou HJ, Zhang H, Qin L, Hwa J, Yun Z, Ji W, Min W (2015) SENP1-mediated NEMO deSUMOylation in adipocytes limits inflammatory responses and type-1 diabetes progression. *Nat Commun* 6: 8917

Sharifi R, Morra R, Appel CD, Tallis M, Chioza B, Jankevicius G, Simpson MA, Matic I, Ozkan E, Golia B *et al* (2013) Deficiency of terminal ADP-ribose protein glycohydrolase TARG1/C6orf130 in neurodegenerative disease. *EMBO J* 32: 1225 – 1237

Shi H, Sun L, Wang Y, Liu A, Zhan X, Li X, Tang M, Anderton P, Hildebrand S, Quan J *et al* (2021) N4BP1 negatively regulates NF-κB by binding and inhibiting NEMO oligomerization. *Nat Commun* 12: 1379

Solier S, Pommier Y (2009) The apoptotic ring: a novel entity with phosphorylated histones H2AX and H2B and activated DNA damage response kinases. *Cell Cycle* 8: 1853 – 1859

Stilmann M, Hinz M, Arslan SC, Zimmer A, Schreiber V, Scheidereit C (2009) A nuclear poly(ADP-ribose)-dependent signalosome confers DNA damage-induced IkappaB kinase activation. *Mol Cell* 36: 365 – 378

Sun S-C (2017) The non-canonical NF-κB pathway in immunity and inflammation. *Nat Rev Immunol* 17: 545 – 558

Szklarczyk D, Gable AL, Lyon D, Junge A, Wyder S, Huerta-Cepas J, Simonovic M, Doncheva NT, Morris JH, Bork P *et al* (2019) STRING v11: protein-protein association networks with increased coverage, supporting functional discovery in genome-wide experimental datasets. *Nucleic Acids Res* 47: D607 – D613

Thul PJ, Lindskog C (2018) The human protein atlas: a spatial map of the human proteome. *Protein Sci* 27: 233 – 244

Trompouki E, Hatzivassiliou E, Tsichritzis T, Farmer H, Ashworth A, Mosialos G (2003) CYLD is a deubiquitinating enzyme that negatively regulates NF-kappaB activation by TNFR family members. *Nature* 424: 793 – 796

Tsherniak A, Vazquez F, Montgomery PG, Weir BA, Kryukov G, Cowley GS, Gill S, Harrington WF, Pantel S, Krill-Burger JM *et al* (2017) Defining a cancer dependency map. *Cell* 170: 564 – 576.e16

Tufan AB, Lazarow K, Kolesnichenko M, Sporbert A, von Kries JP, Scheidereit C (2022) TSG101 associates with PARP1 and is essential for PARylation and DNA damage-induced NF-κB activation. *Zenodo* https://doi.org/10.5281/zenodo.6805594 [DATASET]

Vallabhapurapu S, Matsuzawa A, Zhang W, Tseng P-H, Keats JJ, Wang H, Vignali DAA, Bergsagel PL, Karin M (2008) Nonredundant and complementary functions of TRAF2 and TRAF3 in a ubiquitination cascade that activates NIK-dependent alternative NF-kappaB signaling. *Nat Immunol* 9: 1364 – 1370

Vietri M, Radulovic M, Stenmark H (2020) The many functions of ESCRTs. *Nat Rev Mol Cell Biol* 21: 25 – 42

Wang W, Huang X, Xin H-B, Fu M, Xue A, Wu Z-H (2015) TRAF family member-associated NF-κB activator (TANK) inhibits genotoxic nuclear factor κB activation by facilitating Deubiquitinase USP10-dependent Deubiquitination of TRAF6 ligase. *J Biol Chem* 290: 13372 – 13385

Wang W, Mani AM, Wu Z-H (2017) DNA damage-induced nuclear factor-kappa B activation and its roles in cancer progression. *J Cancer Metastasis Treat* 3: 45 – 59

White JT, Rives J, Tharp ME, Wrabl JO, Thompson EB, Hilser VJ (2021) Tumor susceptibility gene 101 regulates the glucocorticoid receptor through disorder-mediated Allostery. *Biochemistry* 60: 1647 – 1657

Wu Z-H, Shi Y, Tibbetts RS, Miyamoto S (2006) Molecular linkage between the kinase ATM and NF-kappaB signaling in response to genotoxic stimuli. *Science* 311: 1141 – 1146

Zhang QC, Petrey D, Garzón JI, Deng L, Honig B (2013) PrePPI: A structure-informed database of protein-protein interactions. *Nucleic Acids Res* 41: D828 – D833

License: This is an open access article under the terms of the Creative Commons Attribution License, which permits use, distribution and reproduction in any medium, provided the original work is properly cited.

Expanded View Figures

Figure EV1. Genome-wide siRNA screening and hit selection.

A HEK-Luc(tGFP) NF-κB reporter cells were treated with the indicated conditions (50 μM etoposide, 20 Gy irradiation, or 10 ng/ml TNFα). As the NF-κB-driven expression and translation of turbo GFP require more time than the initial activation of the NF-κB pathway, the treatment conditions were alternatively prolonged to 4.5 h. Whole-cell extracts were obtained and immunoblotted with the indicated antibodies.

B Detailed layout of the genome-wide siRNA screening, carried out using a one-plate-to-one target approach. The indicated controls were added to the siRNA library. As another control, cells were treated with the vehicle DMSO (Column 24). The heatmap displays the Z-scores of tested candidates and controls of a representative plate taken from the siRNA library screen.

C Spearman's rank correlation coefficient test of representative screening plate replicates demonstrated high assay reproducibility. Same screening plates and layouts were tested, and results were compared with each other (R1 vs R2).

D TSG101 cluster was visualized using STRING database (Szklarczyk *et al*, 2019). Arrows or lines between candidate hits represent protein–protein interactions observed in previous publications.

E Representative high-confidence DNA damage-selective hits are shown for etoposide (left panel) and TNFα (right panel) screens. The conditions were compared with an ordinary one-way ANOVA (ns, $P > 0.05$; ****$P < 0.0001$). Results were obtained from three biologically independent experiments. Error bars represent mean ± SD.

 © 2022 The Authors

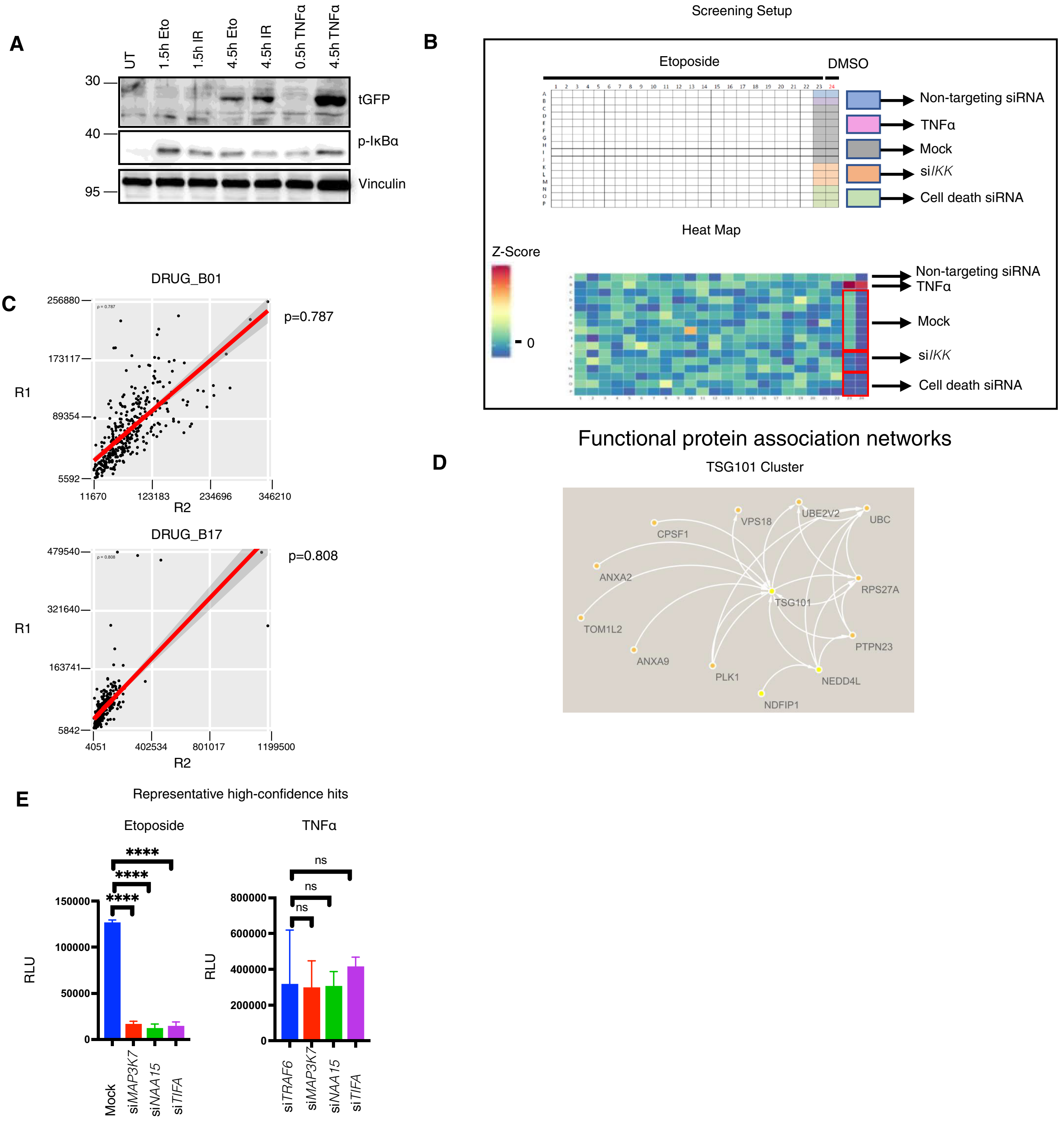
A
UT
1.5h Eto
1.5h IR
4.5h Eto
4.5h IR
0.5h TNFα
4.5h TNFα
30
40
95
tGFP
p-IκBα
Vinculin
B
Screening Setup
Etoposide
DMSO
Non-targeting siRNA
TNFα
Mock
siIKK
Cell death siRNA
Heat Map
Z-Score
0
C
DRUG_B01
p=0.787
R1
R2
DRUG_B17
p=0.808
D
Functional protein association networks
TSG101 Cluster
CPSF1
VPS18
UBE2V2
UBC
ANXA2
RPS27A
TSG101
TOM1L2
ANXA9
PLK1
PTPN23
NEDD4L
NDFIP1
E
Representative high-confidence hits
Etoposide
TNFα
RLU
Mock
siMAP3K7
siNAA15
siTIFA
siTRAF6
ns

Figure EV1.

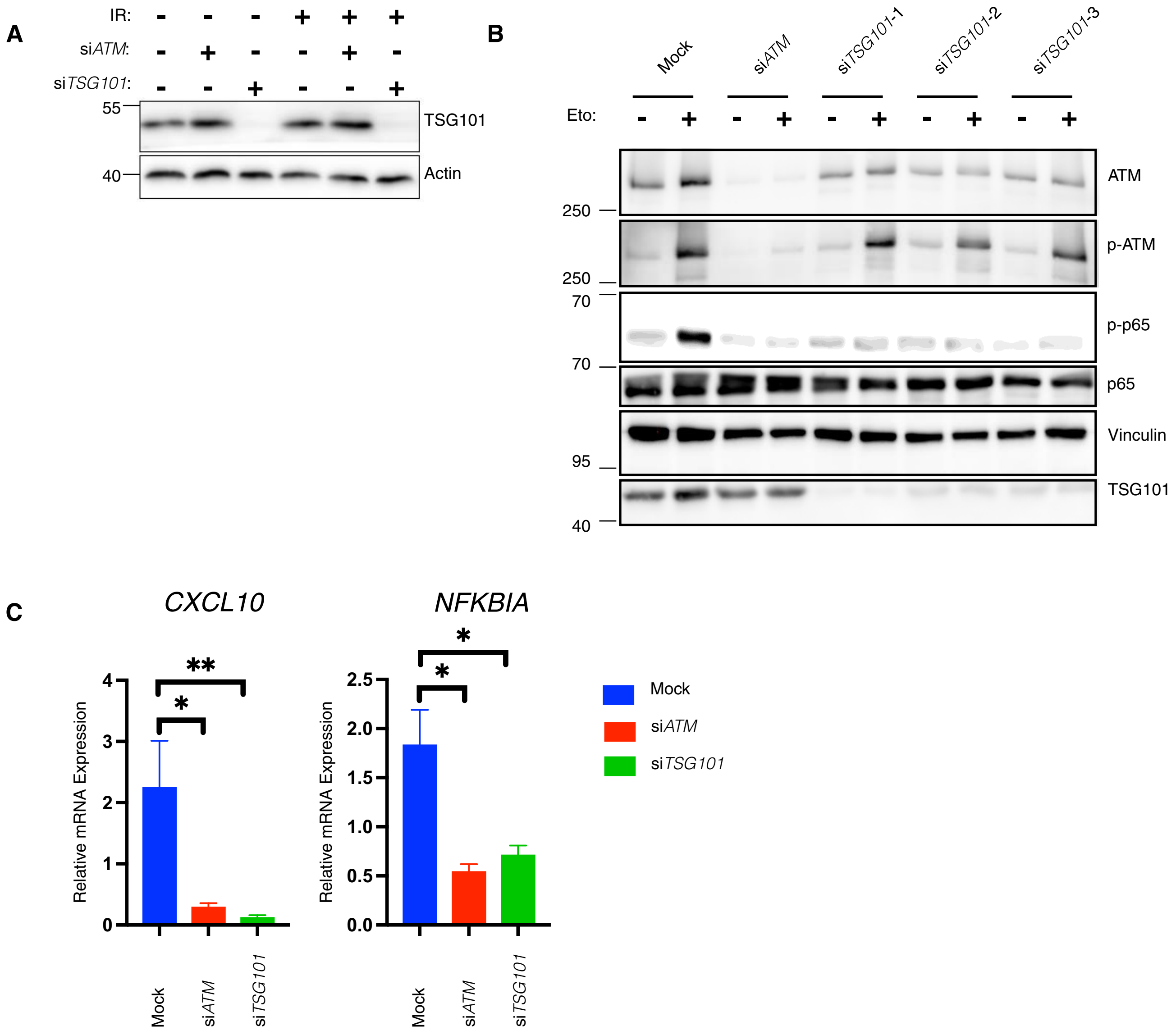

Figure EV2. TSG101 and ATM are equivalently essential for NF-κB activation by DNA damage.

A HEK-Luc(tGFP) NF-κB reporter cells were transfected with the same transfection mixtures as used in Fig 2A. Whole-cell extracts were immunoblotted with antibodies against the indicated proteins. Actin was used as a loading control.

B U2-OS cells were transfected with nontargeting (mock) or *ATM*-directed siRNAs or three different *TSG101*-targeting siRNAs. The indicated cells were treated with etoposide (50 μM, 90 min. Before analysis). Whole-cell extracts were immunoblotted with the indicated antibodies.

C U2-OS cells were transfected with the indicated siRNAs. The NF-κB pathway was activated by etoposide treatment (50 μM, 90 min before analysis). Expression of indicated genes was analyzed using qRT–PCR. The mRNA expression of these genes was normalized to the expression of three housekeeping genes, *ACTA1*, *RPL13A*, and *TBP2*. The gene expression for the indicated conditions is relative to the nontargeting siRNA-transfected vehicle (DMSO)-treated cells. The result is representative of three biologically independent experiments. The conditions were compared with an ordinary one-way ANOVA (*$P < 0.05$; **$P < 0.01$). Error bars represent mean ± SEM.

 © 2022 The Authors

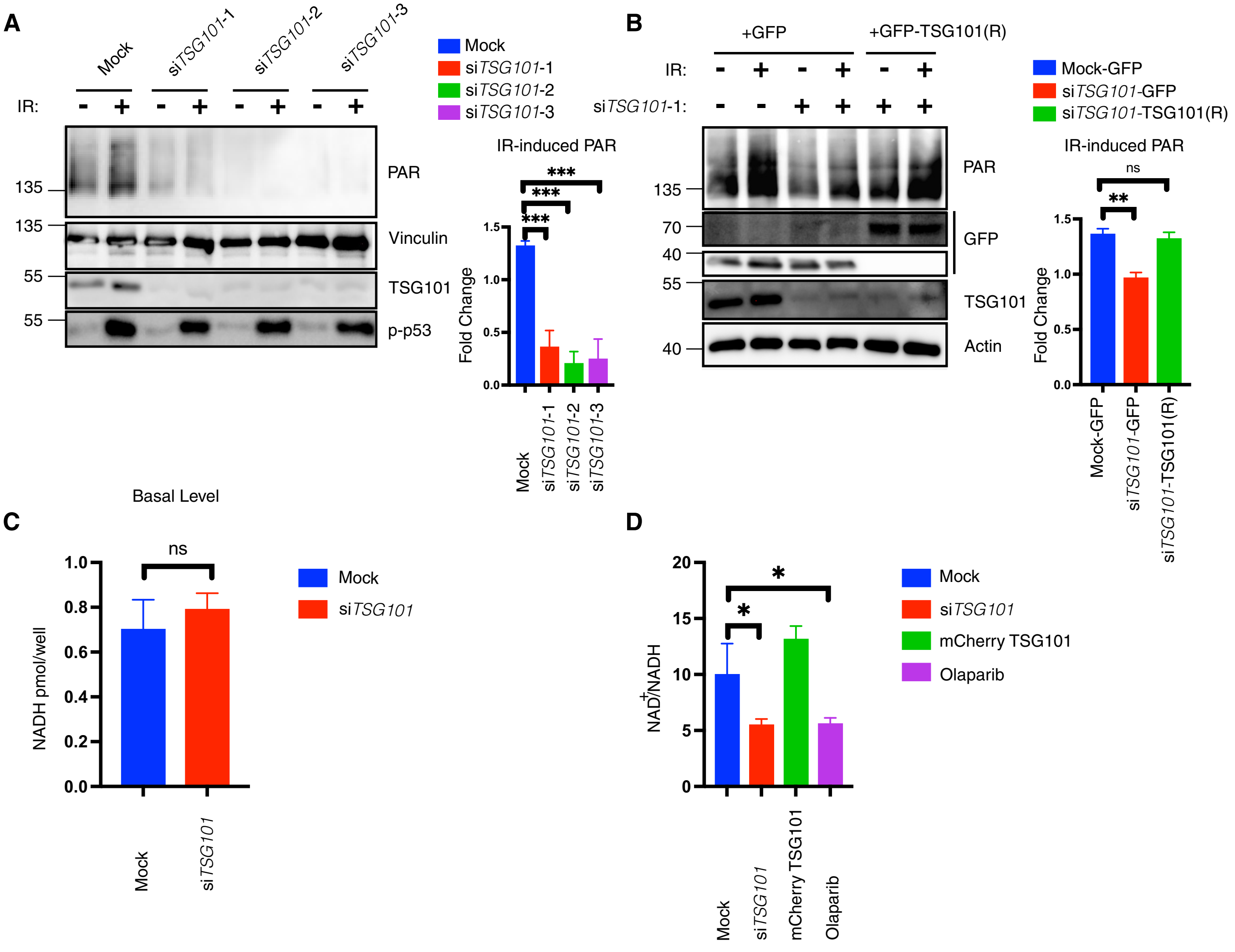

Figure EV3. TSG101 is essential for PARylation.

A U2-OS cells were transfected with nontargeting (mock) or three different *TSG101*-targeting siRNAs. Cells were irradiated (20 Gy) 10 min before analysis. Nuclear extracts were immunoblotted with the indicated reagents and antibodies. Right panel, densitometric measurements of irradiation-induced fold changes in PARylation were obtained from three independent experiments and conditions were compared with an ordinary one-way ANOVA (***$P < 0.001$). Error bars represent mean ± SD.

B A rescue experiment was performed using a siRNA-resistant GFP-TSG101(R) expression vector. Two base substitutions were introduced into the codons for amino acids 61 and 62 of TSG101. U2-OS cells were transfected with either GFP alone or GFP-TSG101 (R) vector. Cells were then transfected with nontargeting or *TSG101*-targeting siRNAs as indicated and were irradiated (20 Gy) 10 min before analysis. Nuclear extracts were immunoblotted with the indicated reagents and antibodies. Densitometric measurements of irradiation-induced fold changes in PARylation (right panel) were obtained from three independent experiments. Conditions were compared with an ordinary one-way ANOVA (ns, $P > 0.05$; **$P < 0.01$). Error bars represent mean ± SD.

C U2-OS cells were transfected with nontargeting or *TSG101*-targeting siRNAs. NADH measurements were performed 72 h after the siRNA transfection. Conditions were compared with Student's *t*-test (Welch Correction) (ns, $P > 0.05$). Error bars represent mean ± SD. Results were obtained from three biologically independent experiments.

D U2-OS cells were transfected with nontargeting (Mock) or *TSG101*-directed siRNAs, with mCherry-*TSG101*, or treated with olaparib (10 μM, 16 h before irradiation), as indicated. Cells were irradiated (20 Gy) 10 min before analysis and NAD+/NADH levels were determined. The result is representative of four independent experiments. The conditions were compared with an ordinary one-way ANOVA (*$P < 0.05$). Error bars represent mean ± SD.

© 2022 The Authors

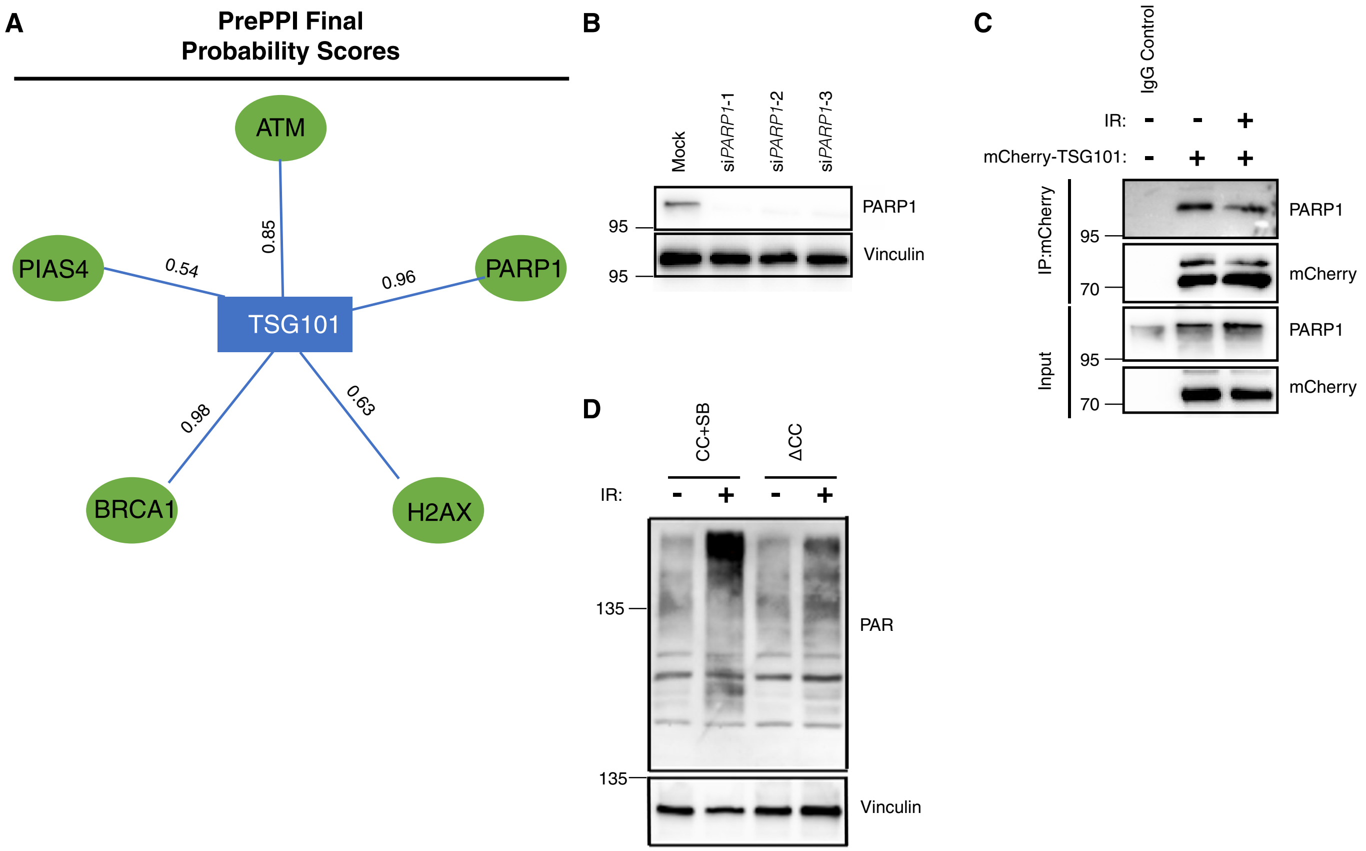

Figure EV4. TSG101 interacts with and enzymatically activates PARP1.

A Final protein–protein interaction probability scores for the indicated interactions were obtained from the PrePPI database (Zhang *et al*, 2013).

B Validation of PARP1 knockdown in A. Different PARP1 siRNAs were used in each independent PLA experiment summarized in Fig 4B. The representative image in Fig 4A was obtained using siPARP1(3).

C U2-OS cells were transfected with the full-length mCherry-tagged TSG101 plasmid and DNA damage was induced by irradiation (20 Gy, 45 min before analysis), as indicated. Immunoprecipitation of mCherry was performed using whole-cell extracts. The result is representative of three biologically independent experiments.

D TSG101 deletion constructs CC+SB and ΔCC were expressed in U2-OS cells. Untreated or irradiated cells were harvested (90 min after 20 Gy exposure) and analyzed by Western blotting for PAR levels using the Pan ADPr reagent. Vinculin was detected as a loading control.

 © 2022 The Authors

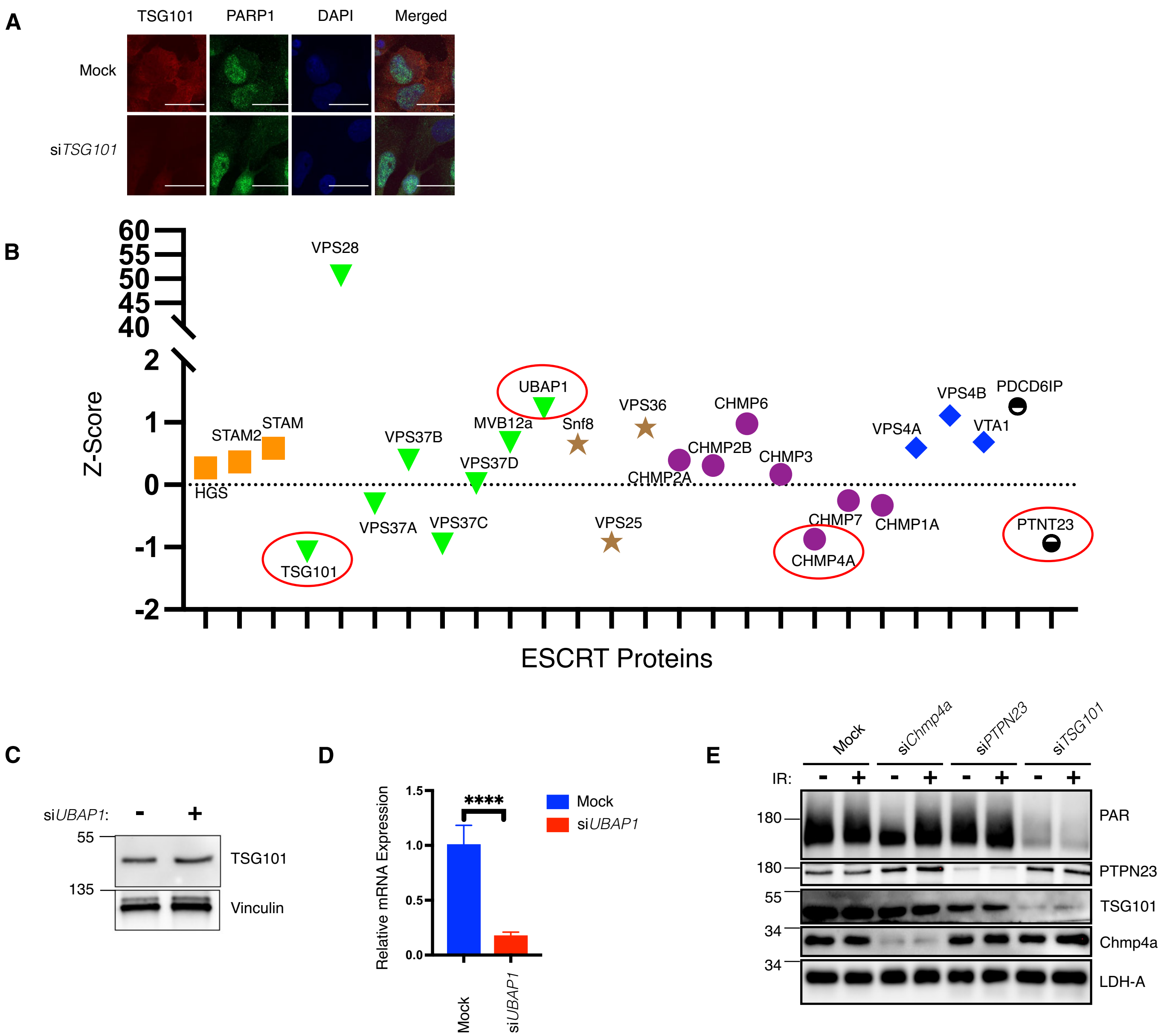

Figure EV5. Control of PARylation by TSG101 is ESCRT complex-independent.

A U2-OS cells were transfected with nontargeting (mock) or *TSG101*-targeting siRNAs. Indirect immunofluorescence visualizes TSG101 (red) and PARP1 (green). DAPI staining shows the nuclei in blue. Scale bar is 10 μm. The image is representative of three biologically independent experiments.

B Z-scores of the mean values of the depicted ESCRT complex members and accessory proteins (ESCRT-0: orange, ESCRT-I: green, ESCRT-II: brown, ESCRT-III: purple, and ESCRT-IV: red) from Dataset EV1. Subsequently analyzed members CHMP4A, PTPN23, TSG101, and UBAP1 are highlighted with red circles.

C U2-OS cells were transfected with nontargeting or *UBAP1*-targeting siRNAs. Whole-cell extracts were immunoblotted with antibodies against the indicated proteins. Vinculin was used as a loading control.

D Total RNA was extracted from cells analyzed in B and converted to cDNA. The relative mRNA expression of *UBAP1* was normalized to two housekeeping genes, *RPL13A*, and *TBP2*. Data are from six biologically independent experiments. Conditions were compared with an unpaired *t*-test with Welch's correction (****$P < 0.0001$). Error bars represent mean ± SD.

E U2-OS cells were transfected with nontargeting siRNAs (mock) or with siRNAs directed against *CHMP4A*, PTPN23, or TSG101 and irradiated (20 Gy) or not, as indicated. PAR levels and expression of indicated proteins were monitored by western blotting with the respective agents and antibodies. LDH-A served as a loading control.

Figure EV6. Efficient apoptosis protection and DNA repair following DNA double strand break generation requires TSG101.

A U2-OS cells were lentivirally transduced with two independent guide RNAs targeting the *TSG101* locus. Due to the lethal side effects of TSG101 deletion on long-term proliferation, bulk cells were used instead of the clonally expanded cells. DNA damage was induced by irradiation (20 Gy, 3 or 9 h before analysis) in wild-type or TSG101 CRISPR knockout bulk cells. Total mRNA was extracted and expression of *NUAK*, *PTX3*, and *PUMA* was analyzed with RT–qPCR. The mRNA expression was normalized to the housekeeping genes *ACTA1*, *RPL13A*, and *TBP2*. Data are from three biologically independent experiments. The conditions were compared with an ordinary one-way ANOVA (***$P < 0.001$; ****$P < 0.0001$). Error bars represent mean ± SEM.

B The efficiency of *TSG101* CRISPR in the cells used in A is shown with immunoblotting. Whole-cell extracts were immunoblotted with antibodies against TSG101. Vinculin was used as a loading control.

C Irradiation-induced cleaved caspase-3 activation in nontargeting gRNA or *Tsg101*-targeting gRNA transduced MEF cells was measured using a colorimetric assay. For knockout efficiencies see Fig EV6E. Bulk cells from *Tsg101* guide-1 were used in this assay. Results were obtained from four biologically independent experiments. The conditions were compared with an unpaired *t*-test (****$P < 0.0001$). Error bars represent mean ± SD.

D Percentage of γH2AX positive staining from Fig 6D is shown. Results were obtained from blind counting of approximately 100 cells for each condition from 3 biologically independent experiments. The conditions were compared with an unpaired *t*-test (****$P < 0.0001$). Error bars represent mean ± SD.

E Wild-type MEF cells from Fig 6J (control or *Tsg101* guide RNA transduced cells) were analyzed for *Tsg101* CRSIPR/Cas9 knockout efficiency. Whole-cell extracts were obtained from these cells and immunoblotted with the indicated antibodies. Vinculin was used as a loading control.

F U2-OS cells were transfected with nontargeting (mock) or two different *TSG101*-targeting siRNAs. Nonirradiated cells (samples 1–3) were compared with senescent cells 7 days postirradiation (samples 4–6). Total mRNA was extracted and expression of *CXCL8*, *IL-6*, and *TSG101* was analyzed by RT–qPCR. The mRNA expression of these genes was normalized to the three housekeeping genes *ACTA1*, *RPL13A*, and *TBP2*. Data are from three biologically independent experiments. Conditions were compared with an ordinary one-way ANOVA (ns, $P > 0.05$; *$P < 0.05$; **$P < 0.01$, ***$P < 0.001$). The conditions were compared with an unpaired *t*-test (****$P < 0.0001$). Error bars represent mean ± SEM.

G Representative brightfield images of *TSG101*-targeting or nontargeting siRNA-transfected *BRCA1* wild-type and mutant MDA-MB 231 and 436 breast cancer cells are shown.

 © 2022 The Authors

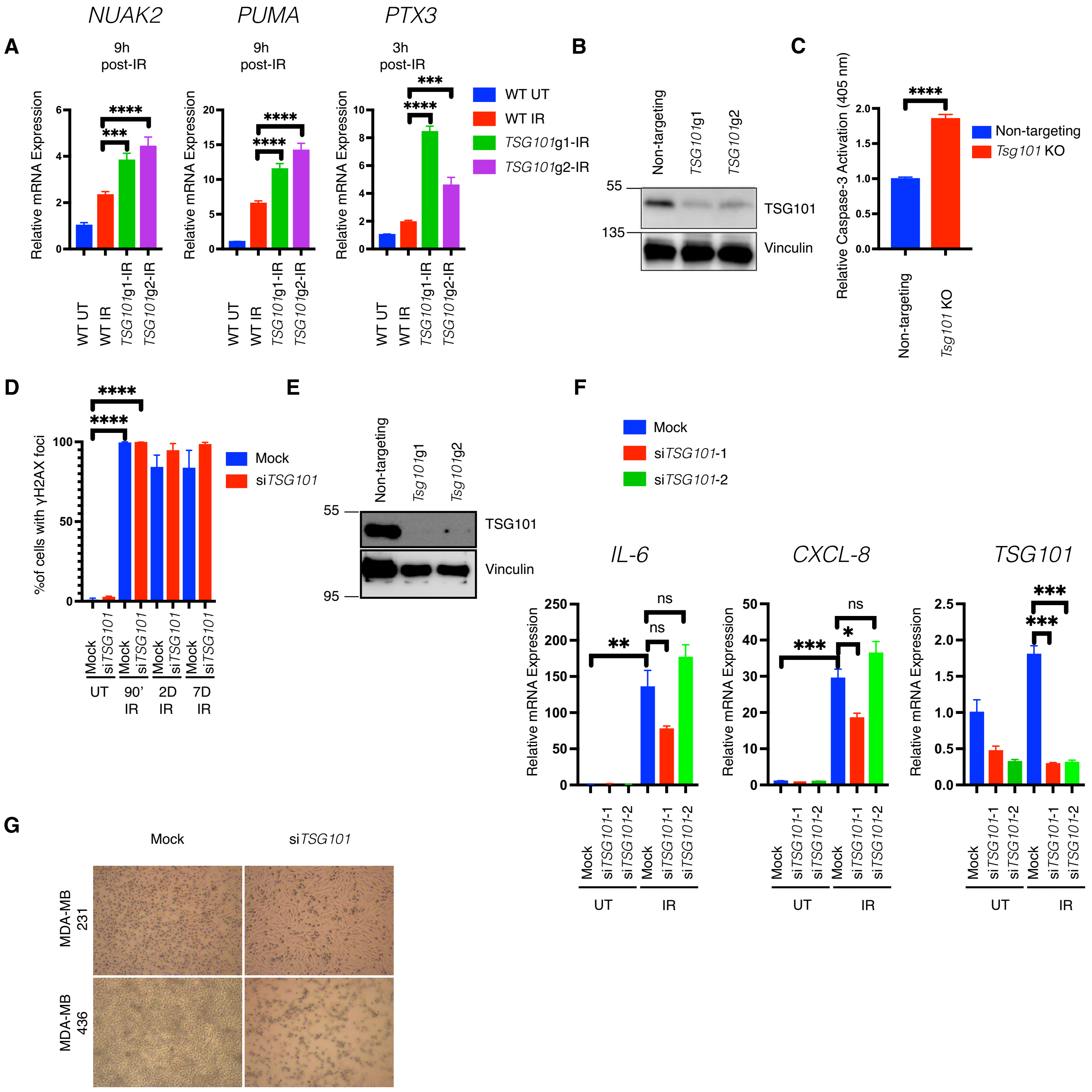
A
NUAK2
9h post-IR
PUMA
9h post-IR
PTX3
3h post-IR
Relative mRNA Expression
WT UT
WT IR
TSG101g1-IR
TSG101g2-IR
B
Non-targeting
TSG101g1
TSG101g2
55
135
TSG101
Vinculin
C
Relative Caspase-3 Activation (405 nm)
Non-targeting
Tsg101 KO
D
%of cells with γH2AX foci
Mock
siTSG101
UT
90' IR
2D IR
7D IR
E
Non-targeting
Tsg101g1
Tsg101g2
55
95
TSG101
Vinculin
F
Mock
siTSG101-1
siTSG101-2
IL-6
CXCL-8
TSG101
Relative mRNA Expression
UT
IR
ns
G
Mock
siTSG101
MDA-MB 231
MDA-MB 436

Figure EV6.
