# Supplementary material for: TSG101 associates with PARP1 and is essential for PARylation and DNA damage‐induced NF‐κB activation
Source: EMBO J. 2022 Sep 20;41(21):e110372. doi: 10.15252/embj.2021110372 (PMC9627669; doi:10.15252/embj.2021110372)
Supplement: Supplementary file 15 [file EMBJ-41-e110372-s015.pdf]

THE EMBO JOURNAL

TSG101 Associates with PARP1 and is Essential for PARylation and DNA Damage-induced NF-κB Activation

Ahmet Bugra Tufan, Katina Lazarow, Marina Kolesnichenko, Anje Sporbert, Jens Peter von Kries, and Claus Scheidereit
DOI: 10.15252/embj.2021110372

Corresponding author(s): Claus Scheidereit (scheidereit@mdc-berlin.de)

Review Timeline:

Submission Date:	6th Dec 21
Editorial Decision:	17th Jan 22
Revision Received:	24th Jun 22
Editorial Decision:	1st Aug 22
Revision Received:	11th Aug 22
Accepted:	25th Aug 22

Editor: Hartmut Vodermaier

Transaction Report:

(Note: With the exception of the correction of typographical or spelling errors that could be a source of ambiguity, letters and reports are not edited. Depending on transfer agreements, referee reports obtained elsewhere may or may not be included in this compilation. Referee reports are anonymous unless the Referee chooses to sign their reports.)

1st Editorial Decision 17th Jan 2022

Thank you for submitting your manuscript on TSG101 in PARP1-mediated NF-kB activation to our editorial office. With some delay related to the holiday break, we have now received the reports from the expert referees (copied below), and further discussed them within our team. Our conclusion from these considerations was, I regret to say, that the study appears presently too preliminary to warrant publication in The EMBO Journal.

As you will see, the referees acknowledge the potential interest of identifying TSG101 as a novel mediator of PARP1 activation during the DNA damage response, but remain (to varying degrees) unconvinced that such possibly far-reaching conclusions are strongly justified based on the present dataset. Recurrent concerns relate to partly subtle/inconsistent results, use of only a single siRNA without rescue in many key experiments, and insufficient analyses of several PARP1-related aspects, including the potentially important synthetic lethality claims. Referee 1 additionally raises a main caveat regarding the conclusions on an ESCRT-independent function of TSG101 in PARP1 regulation. Furthermore, the reviewers also note that stronger PLA and biochemical data would be required to support direct TSG101-PARP1 interactions, also given the apparent lack of TSG101 recruitment to sites of damage. I will not to go through all points of criticism in detail here, but hope you appreciate the given their extent and consequence, I do not see myself in a position to to invite a formal revision of the present manuscript for The EMBO Journal.

Nevertheless, should further work along the lines of the referees' comments and suggestions allow you to obtain more definitive insights and stronger support for the proposed model, I would in light of the overall interest of the findings remain open to looking once more at a new version of the study (accompanied by a detailed response letter) at a future point. With the required extension and substantiation falling beyond the scope of a regular revision and also being of unclear outcome, I hope you understand that I presently cannot make any strong commitments to such a resubmission, which would only be sent back to our referees if we felt that their key issues may been satisfactorily answered and the novelty at the time of resubmission still intact.

I am sorry that the reports do not allow me to be more positive at the current stage, but hope that you will nevertheless find the referees' comments and suggestions helpful when considering how to proceed further with this study. Thank you once more for having had the opportunity to consider this work for publication.

****

Referee #1:

The manuscript by Tufan et al reports a novel role of TSG101 in the activation of NF-κB and PARP1 induced by DNA damage. TSG101 is best known as a component of a multifunctional ESCRT-I complex (endosomal sorting complex required for transport), acting in various processes involving remodeling of intracellular membranes. The authors further identify an interaction of TSG101 with PARP1. Mechanistically, depletion of TSG101 is proposed to inhibit the formation of a nuclear PARP1-IKKγ signalosome required for NF-κB activation. The authors postulate that this function of TSG101 is independent of its

contribution to ESCRT-I. Cells deprived of TSG101 are defective in DNA repair and prone to apoptosis. Finally, the authors propose a synthetic lethality interaction between TSG101 and BRCA1/2.
Overall, the topic is interesting and the findings could potentially provide novel insights into DNA damage-induced NF-κB activation. Unfortunately, for the most part the results are unconvincing or flawed, at places overinterpreted, lacking important controls, with poor or missing description. All this makes the study difficult to understand and the data hard to interpret. As it stands, the manuscript does not conform to standards of proper data generation, description and analysis.

Major issues
1. Practically all data and conclusions of the study are based on one (!) siRNA used to deplete TSG101. This is not acceptable in the light of very common off-target effects of siRNAs. There is only one panel (EV6F) where two siRNAs against TSG101 are used and they exhibit opposite effects although their silencing efficiency is similar: they decrease or increase expression of IL-6 and CXCL8 in comparison to mock control. This casts serious doubts about the validity of most conclusions of the study. Towards the end, in a few panels the authors perform CRISPR/Cas9 knockout of TSG101 using two sgRNAs (Fig. 6J and EV6A-E). However, this is insufficient and all key points of the study would need to be documented with at least 2 consistently acting siRNAs, sgRNAs and/or rescue experiments.
2. Generally the manuscript lacks many important details of experimental procedures (poor description in the main text, figure legends and methods; see details below). In many places the reader is left to guess what exactly was done. This concerns for example the starting point of the study, namely a genome-wide siRNA screen. Data of which screen (in the absence or presence of etoposide) are reported in Table EV1? In this table, what are the descriptions of GENO, EXTD? It seems that probably 3 siRNAs were used against TSG101 in the screen but their results (luminescence reads and Z-scores) are not consistent. It remains unclear which siRNA was further used throughout the study and on which basis it was selected. Moreover, did the authors compare the NF-κB activation in siRNA-transfected cells treated or not with etoposide? Which "visual inspection of the transfected cells" was performed in the screen?
3. In many figures, it is not clear if cells were treated with etoposide or irradiated to induce DNA damage and the exact treatment conditions were not specified. It is not explained why sometimes etoposide and sometimes irradiation were used, so the choice of treatment looks accidental.
4. There are inconsistencies and unclear data regarding PARylation experiments. E.g. Fig. 4D (for which there is no legend at all because of wrong listing/numbering): if EV stands for empty vector transfection as control conditions, why there is no increase in PARylation upon irradiation, like shown in Fig. 3B and 3G? Further in Fig. 4D, overexpression of TSG101 constructs deltaCC or UEV (not binding PARP1) should be tested to confirm the requirement of the CC domain of TSG101 for extensive PARylation upon DNA damage. In parallel, blotting for mCherry should be done to confirm expression of deletion constructs. Moreover, throughout the manuscript the description for "10H PAR" designation on blots is not provided and the difference to blotting against PanADPr is not understandable. In Fig. EV3C, why is PARylation the highest in control cells and even lower upon etoposide treatment?
5. Fig. 4F, the claimed dose-dependence of PARylation on TSG101 levels is not obvious from the presented blot. Moreover, to show the specificity of TSG101 in inducing PARylation, another unrelated protein should be used as negative control.
6. PLA experiments (Fig. 4A) require important negative controls to confirm the specificity of primary antibodies. For this, PLA assay with a set of PARP1+TSG101 primary antibodies should be performed in cells transfected with siTSG101 or siPARP1 to demonstrate no signal in the absence of a given antigen. The specificity control in EV5A is not convincing, as some residual TSG101 staining in the nucleus of siTSG101 transfected cells is visible that by eye appears to be more intense than in the nucleus of mock transfected cells. More images should be presented and quantitatively analyzed to demonstrate lack of nuclear TSG101 staining in siTSG101 transfected cells.
7. Neither immunoprecipitation, nor PLA experiments prove that the interaction of TSG101 and PARP1 is direct, as claimed by the authors.
8. The authors claim that the newly described function of TSG101 is independent of ESCRT-I and ESCRT in general. Both conclusions are not supported by the data. In contrast to what it is written, the authors are not the first to show decreased levels of TSG101 protein upon depletion of another ESCRT-I component VPS28. Cross-destabilization of various ESCRT-I components was investigated previously and downregulation of TSG101 upon VPS28 depletion was reported (https://doi.org/10.1242/jcs.250951). According to that work, knock-down of TSG101 caused loss or strong depletion of all ESCRT-I components, while depletion of other ESCRT-I members (especially the redundant ones such as UBAP-1) caused less pronounced effects. In fact, it is believed that UBAP-1 is specific for ESCRT-I complexes acting in endosomal sorting, whereas versions of ESCRT-I containing MVB12 paralogs instead of UBAP-1 act in other cellular processes (https://doi.org/10.1242/jcs.140673). The authors use UBAP-1 depletion to argue for an ESCRT-I-independent role of TSG101. However, it cannot be excluded that depletion of UBAP-1 (Fig. 5G) does not affect PARP1-GFP recruitment kinetics due to the loss of just one redundant ESCRT-I component instead of an almost complete loss of all ESCRT-I members (as in TSG101-depleted cells).
Similarly, the authors cannot claim that the role of TSG101 is independent of all ESCRT complexes because they did not test other ESCRT subunits. There is a striking omission in Fig. EV5B (which is uninformative and unclear, see below) of PTPN23 and CHMP4A (an accessory TSG101 interactor and an ESCRT-III component, respectively) that show up also as putative positive regulators of NF-κB in the genome-wide screen (based on data in Table EV1; these results were not commented on in the manuscript). Why are these genes not shown in EV5B? Do they act via mechanisms different than TSG101 on NF-κB activity in cells with DNA damage? Depletion of these proteins could have been used to verify the postulated ESCRT-independent function of TSG101.
9. For Fig. EV5B to be informative, it should present the results of the genome-wide screen for all ESCRT family members and

accessory proteins, grouped into ESCRT subcomplexes, with an indication for each member whether or not its knockdown positively/negatively affected NF-κB activation upon DNA damage.
10. The conclusion about the synthetic lethality of TSG101 and BRCA1/2 is at best questionable. The authors use a very fragmentary set of (somewhat outdated - 21Q1) DepMap data to support their claims by presenting just 6 BRCA1-deficient breast cancer cell lines in Fig. 6I (what "BRCA1-deficient" means has not been specified). However, an analysis of updated (21Q4) DepMap set shows that in 1045 out of 1054 cell lines TSG101 is an essential gene whose depletion alone compromises cell viability. It is still possible that BRCA1 deficiency would make TSG101-depleted cells die faster but this should be shown by dedicated experiments with depletion or knockout of BRCA1 combined with depletion of TSG101 in BRCA1 wild-type cells, such as MDA-MB-231 and some further lines. Moreover, MDA-MB-231 cells should also be included in the analysis of up-to-date DepMap data, along with median TSG101 dependency of BRCA1 wildtype cells instead of median for all breast cancer cell lines.
11. All immunoblotting data in the manuscript are not quantitative and only representative blots are shown. To demonstrate the reproducibility of observed phenotypes, densitometry analysis of immunoblotting images for all biological replicates should be performed (at least for main figures) followed by statistical analyses . Also, if needed, protein abundance should be adjusted to a normalization control (e.g. vinculin) (like in EV3G). Moreover, blots are often overexposed and overexposed loading controls make it difficult to confirm the equal loading of wells (e.g. EV3D, G).

Minor issues
- Page 7: "Collectively, our data show therefore that TSG101 functions like an essential cofactor for PARP1 and regulates the DNA damage-induced NF-κB pathway activation by enabling PAR-dependent interactions between ATM, PARP1, IKKγ, and PIASy." This conclusion is too far reaching, since the authors did not show altered interactions between PARP1 and other proteins upon TSG101 downregulation.
- Fig. EV3G, decreases in protein levels of PARP1 and TSG101 in NAA15-depleted cells should be analyzed by densitometry and protein levels corrected with loading control. In a presented blot it is difficult to assess to what extent these proteins are downregulated under examined conditions.
- Is there any particular reason for using the U2-OS cell line in parallel to HEK293? What is the BRCA1 status in U2-OS cells?
- Fig. EV6A, as control, basal mRNA levels of NUAK2, PUMA and PTX3 in untreated TSG101 knockout cells should be shown to confirm that expression of these genes is induced by irradiation and modulated by TSG101 deficiency.
- Fig. 6J - please indicate clearly that the presented image was assembled from two separate culture plates.

Omissions/unclarities in figure legends
- Fig. 1A, how RLU were calculated for siRNA targeting a given gene (is it a value for a single siRNA, averaged values for all if more than one were used? another method?)
- Fig. EV1D - which hits from the primary screen were selected for the analysis: positive, negative or both?
- Fig. 2C - the values are "relative', what is 1 then? An expression of a given gene in non-irradiated cells? No information on statistical analysis.
- Fig. EV2A - the authors should present p-ATM and p-65 status upon irradiation combined with TSG101 deficiency (to compare with Fig. 2B)
- Fig. EV2B - no y-axis values given, how to interpret relative NFKBIA expression scored as almost 1, does etoposide induce expression of NFKBIA at all?
- Fig. 3C and D - TSG101 blots look the same, this should be stated in the legend
- Fig. 6B - what are mock conditions? No error bars, no information on the number of experiments for statistical analysis.
- Fig. 6D-G - is TSG101 depletion by siRNA still present after 7 days post-transfection? Immunoblotting control should be provided.
- Fig. EV6D - wrong description of colors used for graph bars
- In many panels: what is mock control - etoposide-treated/irradiated cells with or without control non-targeting siRNA transfection? It should be clearly stated in each figure legend.
- In many panels: no explanation of abbreviations: e.g. UT/IR, 10H PAR, EV
- In many panels: no information on statistics used and error bars presented on the graphs
- No or misleading information on the exact n number, e.g. in Fig. 2A "the data represent three biological and five technical replicates" so how was the n number calculated, 3 or 5?
- Data of qRT-PCR should be averaged from several independent experiments, instead of showing a representative experiment like in Fig. 2C. For other qRT-PCR panels (Fig. 3F, 6C) there is no information on reproducibility at all.

Omissions in Methods
- No catalogue numbers/manufacturers provided for crucial reagents (like siRNA, drugs, NAD assay kit)
- No information on non-targeting siRNA sequence(s)
- No statistical analysis information provided for non-fluorescent tests (like in EV2)
- 'Key Resources Table for the secondary antibodies and the fluorophores' not provided

Referee #2:

Tufan et al. performed a genome-wide siRNA screen to identify regulators of the NF-κ
B pathway, based on a luciferase reporter readout. While the initial screen was done with the DNA damaging agent etoposide, a

counter screen was performed for selected hits with the pro-inflammatory cytokine TNFα. This allowed for identification of NF-κB activators that are specific to the DNA damage-induced pathway. The screens identified several known and expected NF-κB regulators, but also some new ones, including TSG101. The authors validate that TSG101 is involved in DNA damage-induced NF-κB activation and NF-κB target gene expression and reveal that TSG101 interacts with PARP1 and is required for PARP1-mediated PAR formation. In the absence of TSG101, impaired PAR formation is associated with elevated PARP1 trapping on chromatin and reduced formation of a PARP1-IKKγ signalosome needed for IKK-NF-κB activation. Furthermore, absence of TSG101 phenocopies the effect of PARP inhibition, causing synthetic lethality in BRCA-deficient cells. Overall, this is an original, well-written study and the identification of TSG101 as novel PARP1 activator being required for PARP1-IKKγ signalosome formation and NF-κB activation after DNA damage is interesting, even though the mechanism how TSG101 activates PARP1 remains unclear. There are several additional points, however, which are not fully convincing, and which would require additional experimental validation:

Main points:

1) Most experiments were done with a single siRNA against TSG101. To rule out off-target effects, at least a second independent siRNA against TSG101 should be included to confirm key results, such as the NF-κB activation and NF-κB target gene induction in Fig. 2, the PARP1 activation and PAR formation in Fig. 3, the PARP1 retention on damaged chromatin in Fig. 5, and the DNA repair defects and apoptosis in Fig. 6.

2) It would be important to show that the severe defect in PAR formation after TSG101 knockdown (Fig. 3) can be rescued by expression of siRNA-resistant TSG101, and to test if it can be rescued by TSG101 fragments that can/cannot interact with PARP1.

3) The authors write that "TSG101 is required for the induced expression of selected NF-κB target genes (CCL2, CXCL10, ICAM1, CXCL8, NFKBIA)". How was this selection done? Are other DNA damage-induced NF-κB target genes not dependent on TSG101? RNA-seq experiments could clarify this point.

4) PARP1 trapping on chromatin can be analyzed biochemically (PMID: 23118055) or by IF after pre-extraction (PMID: 32755579, PMID: 33462394). At least one of these approaches should be used to complement the PARP1 recruitment kinetics shown in Fig. 5.

5) In the screens, PARG is identified as negative regulator of IKK-NF-κB signaling. Given that TSG101 knockdown impairs IKK-NF-κB signaling by reducing PAR levels, it would be very interesting to test whether PARG inhibition rescues PAR formation and IKK-NF-κB activation in TSG101-deficient cells.

6) For the experiments shown in Fig. 6E and 6G, are similar effects observed when Olaparib is used instead of TSG101 knockdown? And would the combination of TSG101 and Olaparib be epistatic or rather additive/synergistic? In terms of potential clinical relevance, combining PARP inhibition with TSG101 loss could also be interesting in the context of the experiments shown in Fig. 6H and 6J (6J should be quantified).

Additional points:

1) The specificity of the PLA assays should be validated by single knockdowns of the two proteins, TSG101 and PARP1.

2) In Fig. 4E, the control condition seems to be missing.

3) It would be informative to show PARP1 levels for the experiments in Fig. 3B-D.

4) The results shown in Fig. 4F are not very clear. It could be replaced by a better replicate result or repeated.

5) The results shown in Fig. 5G should be quantified as was done in Fig. 5C,D.

6) The specificity of the TSG101 IF signal should be confirmed by TSG101 knockdown.

7) Most PARP inhibitors, including Olaparib, target PARP1, but also inhibit PARP2 (and perhaps also other PARP family members). It would therefore be better to refer to these compounds as "PARP inhibitor/s" rather than "PARP1 inhibitor/s".

8) At the end of the introduction, the authors write "unrepaired DNA foci accumulate". Perhaps better: "γH2AX foci indicating unrepaired DNA damage accumulate".

9) In the discussion, the list of PARP1 interaction partners that regulate PARP1 activity and trapping could easily be extended to include PARP2/3, PARG, TRIP12, RNF4, p97.

10) In the sentence "..., excluding that any remote mechanisms ..." please check grammar.

Referee #3:

EMBOJ-2021-110372 by Tufan et al.
"TSG101 Associates with PARP1 and is Essential for PARylation and DNA Damage-induced NF-κB Activation"

Summary
In Tufan et al., the authors investigated targets in the DNA-damage-dependent NFkB activation pathway. They found the protein TSG101 in their screen that is an activator of NFkB signaling. Moreover, TSG101 interacts with and activates PARP-1-mediated ADP-ribosylation in order to stimulate NFkB activity. While this protein was previously shown to be involved the endosomal sorting complex, this study describes a new role for it. From their results, the authors conclude that TSG101 might serve as "a therapeutic target to achieve synthetic lethality in cancer treatment."

Review
This paper addresses an important aspect of biology related to cancer biology with potential therapeutic implications. The new role for TSG101 is interesting and may have broader implications. Nonetheless, the authors still need to address a few issues, including additional controls.

Strengths: This work discovers a previously unknown function of TSG101 in the DNA-damage response using detailed biochemical assays. This work has potential therapeutic implications.

Weaknesses: Aspects of the work are incomplete and a number of experiments require additional controls.

Additional Comments:

1. In the screen shown in Figure 1, the authors should show where PARP-1 itself is enriched. This is a critical control in view of their subsequent experiments and the overall hypothesis of the study.
2. For Figure 2B, the authors should show the level of total p65 along with the changes in p65 phosphorylation.
3. Figure 3A should include the PAR levels in order to assess the effectivity of PARP-1 inhibition by Olaparib.
4. A major conclusion the authors draw from Figure 3 is that TSG101 is required for PARP-1 interaction with ATM, IKK, and other components of the NFkB signalosome. However, no data has been shown to support this.
5. In Figure 4A, the PLA signal appears to be stronger in the Olaparib treated condition. If this holds true, it goes against the later conclusions. Quantifying the PLA data is therefore essential to support the author's claims.
6. The data in Figure 4E seems counter intuitive. If I understand this correctly, increased binding of PARP-1 to the CC+SB domains should increase the PAR levels and, subsequently, lead to more NAD+ being consumed. However, the results shown here are in the opposite direction. Can the authors clarify this?
7. Is the retention of PARP-1 observed with Olaparib due to PARP trapping? Furthermore, does TSG101 depletion cause the same phenomenon?
8. The in the sentence "We selected the 1.000 top hits as candidate activators of the pathway and 100 further hits," should 1.000 be written as 1000?

*** As a service to authors, The EMBO Journal offers the possibility to directly transfer declined manuscripts to another EMBO Press title (EMBO Reports, EMBO Molecular Medicine, Molecular Systems Biology) or to the open access journal Life Science Alliance launched in partnership between EMBO Press, Rockefeller University Press and Cold Spring Harbor Laboratory Press. The full manuscript (including reviewer comments, where applicable and if chosen) will be automatically forwarded to the receiving journal, to allow for fast handling and a prompt decision on your manuscript. For more details of this service, and to transfer your manuscript to another EMBO title please follow this link:
Link Not Available

Response to the referees' criticisms and comments

Manuscript EMBOJ-2021-110372

Tufan et al. TSG101 Associates with PARP1 and is Essential for PARylation and DNA Damage-induced NF-κB Activation

The original statements are indicated in italics and our responses appear in blue. New text in the revised manuscript is marked in blue. Please note that in addition to the figure alterations and new figure panels listed in the individual responses, all figures were now changed to Adobe Illustrator format.

Editor:

Thank you for submitting your manuscript on TSG101 in PARP1-mediated NF-kB activation to our editorial office. With some delay related to the holiday break, we have now received the reports from the expert referees (copied below), and further discussed them within our team. Our conclusion from these considerations was, I regret to say, that the study appears presently too preliminary to warrant publication in The EMBO Journal.

As you will see, the referees acknowledge the potential interest of identifying TSG101 as a novel mediator of PARP1 activation during the DNA damage response, but remain (to varying degrees) unconvinced that such possibly far-reaching conclusions are strongly justified based on the present dataset. Recurrent concerns relate to partly subtle/inconsistent results, **use of only a single siRNA without rescue in many key experiments**, and insufficient analyses of several PARP1-related aspects, including the potentially important **synthetic lethality claims**. Referee 1 additionally raises a main caveat regarding the conclusions on an **ESCRT-independent function** of TSG101 in PARP1 regulation. Furthermore, the reviewers also note that **stronger PLA and biochemical data would be required to support direct TSG101-PARP1 interactions**, also given the apparent lack of TSG101 recruitment to sites of damage. I will not to go through all points of criticism in detail here, but hope you appreciate the given their extent and consequence, I do not see myself in a position to to invite a formal revision of the present manuscript for The EMBO Journal.

We thank the editor for the synopsis of the major issues that have to be solved. We have successfully addressed all these major points, as well as all further specific points of the three referees.

Nevertheless, should further work along the lines of the referees' comments and suggestions allow you to obtain more definitive insights and stronger support for the proposed model, I would in light of the overall interest of the findings remain open to looking once more at a new version of the study (accompanied by a detailed response letter) at a future point. With the required extension and substantiation falling beyond the scope of a regular revision and also being of unclear outcome, I hope you understand that I presently cannot make any strong commitments to such a resubmission, which would only be sent back to our referees if we felt that their key issues may been satisfactorily answered and the novelty at the time of resubmission still intact.

I am sorry that the reports do not allow me to be more positive at the current stage, but hope that

you will nevertheless find the referees' comments and suggestions helpful when considering how to proceed further with this study. Thank you once more for having had the opportunity to consider this work for publication.

Referee #1:

The manuscript by Tufan et al reports a novel role of TSG101 in the activation of NF-κB and PARP1 induced by DNA damage. TSG101 is best known as a component of a multifunctional ESCRT-I complex (endosomal sorting complex required for transport), acting in various processes involving remodeling of intracellular membranes. The authors further identify an interaction of TSG101 with PARP1. Mechanistically, depletion of TSG101 is proposed to inhibit the formation of a nuclear PARP1-IKKγ signalosome required for NF-κB activation. The authors postulate that this function of TSG101 is independent of its contribution to ESCRT-I. Cells deprived of TSG101 are defective in DNA repair and prone to apoptosis. Finally, the authors propose a synthetic lethality interaction between TSG101 and BRCA1/2.

Overall, the topic is interesting and the findings could potentially provide novel insights into DNA damage-induced NF-κB activation.

We thank the reviewer for agreeing with the general importance of our findings.

Unfortunately, for the most part the results are unconvincing or flawed, at places overinterpreted, lacking important controls, with poor or missing description. All this makes the study difficult to understand and the data hard to interpret. As it stands, the manuscript does not conform to standards of proper data generation, description and analysis.

We have extensively revised the manuscript and have included a number of new experiments, which address all raised issues in full and confirm our previous hypotheses.

Major issues
1. Practically all data and conclusions of the study are based on one (!) siRNA used to deplete TSG101. This is not acceptable in the light of very common off-target effects of siRNAs. There is only one panel (EV6F) where two siRNAs against TSG101 are used and they exhibit opposite effects although their silencing efficiency is similar: they decrease or increase expression of IL-6 and CXCL8 in comparison to mock control. This casts serious doubts about the validity of most conclusions of the study. Towards the end, in a few panels the authors perform CRISPR/Cas9 knockout of TSG101 using two sgRNAs (Fig. 6J and EV6A-E). However, this is insufficient and all key points of the study would need to be documented with at least 2 consistently acting siRNAs, sgRNAs and/or rescue experiments.

We thank the reviewer for these helpful suggestions. In the original submission we used three different siRNAs against TSG101 in the primary genome-wide screen, and three additional different siRNAs for the TNFα counter screen. This is a standard procedure for the Screening Unit in order to minimize siRNA off-target effects. However, we agree with the reviewer and now provide several independent siRNAs for other main experiments. We have now performed all key experiments with three independent siRNAs against TSG101. Using the additional siRNAs, we fully confirmed our previous observations regarding DNA damage-induced p65 phosphorylation (**new Figure EV2B**), NF-κB activation (assessed by EMSA) in **new Figure 2D**, irradiation-induced poly(ADP ribosyl)ation in **new Figure EV3A**, etoposide-induced

cell death in **(Figure for referees R1)**, as well as irradiation-induced caspase 3 cleavage (**Figure for referees R2**).

In Figure EV6F, we showed that TSG101 does not play a dominant role in senescence associated NF-κB-dependent gene expression of so-called SASP (senescence associated secretory phenotype) genes. As we have previously reported, SASP expression detected 7 days after DNA damage induction is independent of PARP signalosome formation and the acute DNA damage response observed hours or less after induction (Kolesnichenko et al., EMBO J 2021). In agreement with this, expression of both cytokines was still upregulated in the absence of TSG101. The increases in gene expression are larger than 100 or 20-fold in *IL6* and *CXCL8* expression, respectively. In this regard, differences amongst first and second siRNA-transfected conditions to non-targeting siRNA transfected condition does not demonstrate opposing effects, they rather display an experimental variation.

We further used two different sgRNAs against *TSG101* in murine and human cells in the original manuscript in **Figures 6J, EV6E** and **Figures EV6A and B**, respectively. In these experiments, these sgRNAs gave entirely consistent results in ablating cell viability of *Brca1*$^{-/-}$ cells and increasing pro-apoptotic gene expression in human cells.

We are also grateful for the suggestions of reviewers 1 and 2 to perform rescue experiments. We designed an siRNA-resistant TSG101 plasmid and were able to show that DNA damage-induced poly(ADP-ribosyl)ation is increased again by ectopic *TSG101* overexpression in cells that carry a knockdown of endogenous *TSG101* (**new Figures EV3B and R3**). Taken together, all of these results reinforce our initial observation that TSG101 is required for cellular generation of PARylation and for PAR-dependent NF-κB activation.

2. Generally the manuscript lacks many important details of experimental procedures (poor description in the main text, figure legends and methods; see details below). In many places the reader is left to guess what exactly was done. This concerns for example the starting point of the study, namely a genome-wide siRNA screen. Data of which screen (in the absence or presence of etoposide) are reported in Table EV1? In this table, what are the descriptions of GENO, EXTD? It seems that probably 3 siRNAs were used against TSG101 in the screen but their results (luminescence reads and Z-scores) are not consistent. It remains unclear which siRNA was further used throughout the study and on which basis it was selected. Moreover, did the authors compare the NF-κB activation in siRNA-transfected cells treated or not with etoposide? Which "visual inspection of the transfected cells" was performed in the screen?

We apologize for the lack of detail of some procedures in the original manuscript and have now included the requested information. The data reported in **Table EV1 (now Dataset EV1)** from the primary screen were generated in the presence of etoposide. We now included detailed information about the screening setup in the Materials and Methods section and also in legends to the table. In **Table EV1 (now Dataset EV1)**, the abbreviations GENO, DRUG, and EXTD originate in the Ambion Silencer Human Genome siRNA library v3. The library is divided by the manufacturer into a “Human Genome” (GENO), “Human Drug” (DRUG), and “Human Drug Extension” (EXTD) subset. In the primary screening the full library was screened.

We used 3 different siRNAs per gene for the initial screen. In total, more than 65.000 siRNAs were used in the primary genome-wide screen, as was indicated in **Figure 1B** and **Table EV1 (now Dataset EV1)**. The variation in efficacy of the single siRNAs per gene is expected because

of assumed differences in knockdown efficiency for different siRNAs. Nevertheless, as pointed out in the paper, we re-identified many known pathway regulators and general regulators, such as the 13 components of the Pol II complex, supported by at least two independent siRNAs. To further refine the results, we performed a counter screen, only with the selected candidates from the primary screen. TSG101 was included in the secondary (counter screen) along with other putative regulators (**Table EV4, now Dataset EV3**), even though one of the siRNAs, as the referee pointed out, did not lead to abrogated NF-κB activation. We postulate that this might be due to lack of efficacy of this particular siRNA under the screening condition, as pointed out above. Our counter screen allowed to refine and validate the targets from the primary screen.

In response to the referee's question regarding the use of etoposide, we did use it in the primary screen and this was stated in the results section and elsewhere. In the counter screen we used etoposide and TNFα, in each case relative to unstimulated controls.

Our Materials and Methods section includes a protocol that was used for visual inspection of the cells in the screen. In brief, the cells were checked by automated microscopy and 25 specimen per well were acquired.

3. *In many figures, it is not clear if cells were treated with etoposide or irradiated to induce DNA damage and the exact treatment conditions were not specified. It is not explained why sometimes etoposide and sometimes irradiation were used, so the choice of treatment looks accidental.*

We have now specified the treatment conditions. The use of etoposide or irradiation was in fact indicated in the captions of all figures and Expanded View figures, except for **Figure 2C** and **Figures EV2B and 6F**, where this information was provided in the legends. The choice of treatment is not accidental: we used both etoposide or irradiation interchangeably to demonstrate that the observed effects are not specific to properties of either treatment or agent but are caused by DNA double strand breaks. We have also done this in our original description of this pathway (Stilmann et al., 2009, Hinz et al., 2010). Importantly, results obtained with both treatments in the various types of experiments are equivalent and confirm our conclusions.

4. There are inconsistencies and unclear data regarding PARylation experiments. E.g. Fig. 4D (for which there is no legend at all because of wrong listing/numbering): if EV stands for empty vector transfection as control conditions, why there is no increase in PARylation upon irradiation, like shown in Fig. 3B and 3G?

We apologize for the omission: EV stands for Empty Vector, and this information is now contained in the legends of the respective figures. However, we have replaced the previous **Figure 4D** with **new Figure EV4D** focusing on the TSG101 deletion mutants CC+SB, containing essentially the coiled coil domain, and ΔCC, devoid of the coiled coil domain (see below).

Further in Fig. 4D, overexpression of TSG101 constructs deltaCC or UEV (not binding PARP1) should be tested to confirm the requirement of the CC domain of TSG101 for extensive PARylation upon DNA damage.

We thank the referee for this very helpful suggestion and now included these data as a new **new Figure EV4D**. Ectopic expression of TSG101 deletion mutant CC+SB strongly enhanced IR-induced PARylation, in contrast to TSG101ΔCC, devoid of the coiled coil domain, which does

not interact with PARP1 (**Figure 4C and D**). Importantly, the different effects on PARylation were mirrored by the increased NADH consumption after CC+SB expression and irradiation compared to ΔCC and the inverse effect on the NAD+/NADH ratio (**Figure 4E**). The experiments were performed in cells that contain endogenous TSG101, so the PARylation induction in response to irradiation of around 1.5-fold we observed in presence of the inactive ΔCC is comparable to the one we detected through quantitation in non-transfected cells (**new Figure EV3A**), whereas the IR-response in the presence of ectopic CC+SB is much higher (**new Figure EV4D)**. These new data strengthen our conclusion that via coiled coil mediated interaction, TSG101 enhances the catalytic activity of PARP1.

Moreover, throughout the manuscript the description for "10H PAR" designation on blots is not provided and the difference to blotting against PanADPr is not understandable. In Fig. EV3C, why is PARylation the highest in control cells and even lower upon etoposide treatment?

We have now added the description of the 10H PAR and PanADPr designations in the legends and in the manuscript. The 10H PAR monoclonal antibody detects PAR chains that are longer than 10 units, whereas the PanADPr reagent detects PAR chains of all sizes, apart from mono ADP ribosylation.

For the entire revision, we introduced eight new figure panels. In order to accommodate all improvements which addressed TSG101 data, we decided to remove the data describing an analysis of ACADVL and NAA15, which did not directly contribute to the main focus of the paper, centered around TSG101. In line with this, we have also removed old figure EV3C. Removal of these figure panels does not change our main conclusions, requirement of TSG101 for any type of PARylation.

Regarding the observed activation of PARylation in untreated control samples: This is caused by known activating effects of shearing forces on PARP by cell harvesting, lysis and fractionation procedures (Jungmichel et al., 2013). We indicated this in the legends of the relevant figures 3A and C. The effect is more evident for weaker (etoposide) compared to stronger (irradiation) PARP1 activators and can be reduced by addition of PARP inhibitors to the extraction buffer. However, the essential point we make is the strict requirement of TSG101 for any type of PAR generation, including the artificial *in vitro* PAR generation in some of the figures.

5. Fig. 4F, the claimed dose-dependence of PARylation on TSG101 levels is not obvious from the presented blot. Moreover, to show the specificity of TSG101 in inducing PARylation, another unrelated protein should be used as negative control.

In the **new Figure 4F**, we show a representative image of the PARylation observed under different conditions. We used only one concentration of TSG101 in the reaction and therefore we are not analyzing dose-dependent increases in PARylation.

In order to demonstrate the specificity of TSG101 in inducing PARylation, we also used similar amounts of unrelated proteins, BSA and RNF113A, as negative controls, as suggested by the referee. RNF113A is similar in size to TSG101. Our data (**new Figure 4F**) confirm again that PARylation by PARP1 is strongly enhanced by TSG101, but not by the other proteins used in equivalent amounts.

6. PLA experiments (Fig. 4A) require important negative controls to confirm the specificity of primary antibodies. For this, PLA assay with a set of PARP1+TSG101 primary antibodies should be performed in cells transfected with siTSG101 or siPARP1 to demonstrate no signal in the absence of a given antigen.

We apologize for this omission and have now included the required negative controls by performing PLA in cells transfected with siRNA (three different siRNAs were used) against PARP1 (**new Figure 4B, new Figure EV4B**). The results demonstrate that the PLA signal depends on the presence of PARP1 and on both antibodies (PARP1 and TSG101) used in conjunction (**new Figure 4A**). We quantitated these results in the **new Figure 4B**.

The specificity control in EV5A is not convincing, as some residual TSG101 staining in the nucleus of siTSG101 transfected cells is visible that by eye appears to be more intense than in the nucleus of mock transfected cells. More images should be presented and quantitatively analyzed to demonstrate lack of nuclear TSG101 staining in siTSG101 transfected cells.

We now replaced these images with representative ones (**new Figure EV5A**). Moreover, as suggested, we have quantitated the signal intensity and confirm that TSG101 staining is specific and also that the TSG101 knockdown worked efficiently, as was shown in several other experiments as well.

7. Neither immunoprecipitation, nor PLA experiments prove that the interaction of TSG101 and PARP1 is direct, as claimed by the authors.

The PLA method depends on the recognition of target molecules in very close proximity (<40 nm) by the pairs of affinity probes and many examples in the literature show that established known direct protein-protein interactions are readily detected. Several of our experiments show that the two proteins interact under different experimental conditions. It is possible, however, as the referee alluded to, that an unknown third molecule is required and that in intact cells the interaction occurs via a third factor. However, the stimulation of PARP1 catalytic activity *in vitro* by TSG101, using only these two purified proteins (**new Figure 4G**) can only be explained by a direct interaction. Nevertheless, we now deleted the word "direct" in the manuscript in context with the TSG101-PARP1 interaction.

*8. The authors claim that the newly described function of TSG101 is independent of ESCRT-I and ESCRT in general. Both conclusions are not supported by the data. In contrast to what it is written, the authors are not the first to show decreased levels of TSG101 protein upon depletion of another ESCRT-I component VPS28. Cross-destabilization of various ESCRT-I components was investigated previously and downregulation of TSG101 upon VPS28 depletion was reported (*https://doi.org/10.1242/jcs.250951*).*

We thank the referee for drawing our attention to this recent study (Kolmus et al., 2021), which we cite now in our article. According to that work, knock-down of TSG101 caused loss or strong depletion of all ESCRT-I components, while depletion of other ESCRT-I members (especially the redundant ones such as UBAP-1) caused less pronounced effects. In fact, it is believed that UBAP-1 is specific for ESCRT-I complexes acting in endosomal sorting, whereas versions of ESCRT-I containing MVB12 paralogs instead of UBAP-1 act in other cellular processes (https://doi.org/10.1242/jcs.140673) We are happy to report that our findings confirm those of that study and we also see destabilization of TSG101 through downregulation of VPS28.

The authors use UBAP-1 depletion to argue for an ESCRT-I-independent role of TSG101. However, it cannot be excluded that depletion of UBAP-1 (Fig. 5G) does not affect PARP1-GFP recruitment kinetics due to the loss of just one redundant ESCRT-I component instead of an almost complete loss of all ESCRT-I members (as in TSG101-depleted cells).

Similarly, the authors cannot claim that the role of TSG101 is independent of all ESCRT complexes because they did not test other ESCRT subunits.

We agree with this point and therefore we performed suggested experiments (**new Figure EV5E**). We show that depletion of PTPN23 or of CHMP4A, in contrast to that of TSG101, has no effect on poly(ADP ribosyl)ation.

There is a striking omission in Fig. EV5B (which is uninformative and unclear, see below) of PTPN23 and CHMP4A (an accessory TSG101 interactor and an ESCRT-III component, respectively) that show up also as putative positive regulators of NF-κB in the genome-wide screen (based on data in Table EV1; these results were not commented on in the manuscript). Why are these genes not shown in EV5B? Do they act via mechanisms different than TSG101 on NF-κB activity in cells with DNA damage? Depletion of these proteins could have been used to verify the postulated ESCRT-independent function of TSG101.

We have now removed **old Figure EV5B** and replaced it by the endpoint results of the primary screening, showing all ESCRT components and accessory proteins, including PTPN23 and CHMP4A (**new Figure EV5B**). As pointed out by the referee, both PTPN23 and CHMP4A are positive regulators of the DNA damaged induced NF-κB pathway, however our analysis of poly(ADP-ribosyl)ation after siRNA-mediated depletion of these proteins (**Figure EV5E**) shows that they have no impact on PARylation. Therefore, they might act via a different mechanism than TSG101 on induced NF-κB reporter activity. That is an interesting possibility, however it is beyond the scope of the current paper.

9. For Fig. EV5B to be informative, it should present the results of the genome-wide screen for all ESCRT family members and accessory proteins, grouped into ESCRT subcomplexes, with an indication for each member whether or not its knockdown positively/negatively affected NF-κB activation upon DNA damage.

We thank the referee for this helpful suggestion and have included it in the **new Figure EV5B**.

10. The conclusion about the synthetic lethality of TSG101 and BRCA1/2 is at best questionable. The authors use a very fragmentary set of (somewhat outdated - 21Q1) DepMap data to support their claims by presenting just 6 BRCA1-deficient breast cancer cell lines in Fig. 6I (what "BRCA1-deficient" means has not been specified). However, an analysis of updated (21Q4) DepMap set shows that in 1045 out of 1054 cell lines TSG101 is an essential gene whose depletion alone compromises cell viability.

It is still possible that BRCA1 deficiency would make TSG101-depleted cells die faster but this should be shown by dedicated experiments with depletion or knockout of BRCA1 combined with depletion of TSG101 in BRCA1 wild-type cells, such as MDA-MB-231 and some further lines. Moreover, MDA-MB-231 cells should also be included in the analysis of up-to-date DepMap data, along with median TSG101 dependency of BRCA1 wildtype cells instead of median for all breast cancer cell lines

We updated the data in **new Fig 6I** using the newest 22Q1 data set of DepMap. Of the 46 breast cancer cell lines contained in the data set, all of the cell lines with damaging BRCA1 mutations scored with much higher sensitivity to CRISPR-mediated TSG101 inactivation than

the average of the cell lines (**Fig 6I**). BRCA2 mutations were much less pronounced. As the referee points out, TSG101 depletion generally impairs cell viability. However, in the presence of inactivated BRCA1, viability is affected at a significantly higher level. To further address the comments above, we specified the *BRCA1/2* deficiency status for each of these cell lines in the **new Table EV3**. This table precisely describes the damaging mutations and it is clear that defective BRCA1 is most critical regarding synthetic lethality with TSG101.

As suggested, synthetic lethality was experimentally verified the by using TSG101 siRNA in BRCA1 deficient and proficient cells (**new Figure 6G**). We would like to point out that our original manuscript already contained data showing siRNA depletion of TSG101 in MDA-MB-231 and MDA-MB-436. We showed that a knockdown of TSG101 significantly decreased the viability of MDA-MB-436 cells bearing mutant BRCA1 and BRCA2 genes, but not of MDA-MB-231 cells with wild type BRCA1/BRCA2 (**old Figure 6H**). Furthermore, in the same panel, we showed that the viability-reducing effect of TSG101 depletion was equivalent to the survival-decreasing effect of the PARP-inhibitor olaparib. We furthermore showed that the combined depletion of TSG101 and addition of olaparib did not significantly cause additional impairment of viability compared to each condition alone, suggesting epistatic regulation, i.e. both treatments likely act through impaired PAR formation (**new Figure 6H**). We believe this exploration of synthetic lethality between TSG101 and BRCA1 and/or other proteins is an exciting research direction and will be pursued in the future, however is beyond the scope of the current project.

11. All immunoblotting data in the manuscript are not quantitative and only representative blots are shown. To demonstrate the reproducibility of observed phenotypes, densitometry analysis of immunoblotting images for all biological replicates should be performed (at least for main figures) followed by statistical analyses. Also, if needed, protein abundance should be adjusted to a normalization control (e.g. vinculin) (like in EV3G). Moreover, blots are often overexposed and overexposed loading controls make it difficult to confirm the equal loading of wells (e.g. EV3D, G).

As suggested, we have quantitated key experiments (**Figures 2D, 4C, 5C, 5D, 6F, EV3A, and EV3B**) using at least three biological replicates. Results of quantitation including statistical analysis appear next to the representative blot. For overexposed blots, we have repeated the experiment and replaced the blot.

Minor issues

- Page 7: "Collectively, our data show therefore that TSG101 functions like an essential cofactor for PARP1 and regulates the DNA damage-induced NF-κB pathway activation by enabling PAR-dependent interactions between ATM, PARP1, IKKγ, and PIASy." This conclusion is too far reaching, since the authors did not show altered interactions between PARP1 and other proteins upon TSG101 downregulation.

We agree that we did not formally show that these interactions are lost upon TSG101 depletion and have rephrased this sentence in the introduction. However, as pointed out in the manuscript, we previously showed that TSG101 is attracted to IKKγ in a DNA damage-induced manner, as expected for a PAR/PARP1 dependent process (Mikuda et al., 2018). We have previously shown in detail that DNA damage-induces an interaction between ATM, PARP1, IKKγ, and PIASy that is PAR-dependent and required for NF-κB activation (Stilmann et al., 2009). Since TSG101 depletion ablates PAR levels, we propose that the link between

TSG101 and DNA damage-induced NF-κB activation occurs through the formation of this short-lived signalosome.

- Fig. EV3G, decreases in protein levels of PARP1 and TSG101 in NAA15-depleted cells should be analyzed by densitometry and protein levels corrected with loading control. In a presented blot it is difficult to assess to what extent these proteins are downregulated under examined conditions.

As explained under point 4, referee 1, upon revision, we took out the previous data regarding NAA15 and ACADVL in order to accommodate new figure panels which are focused on TSG101.

- Is there any particular reason for using the U2-OS cell line in parallel to HEK293? What is the BRCA1 status in U2-OS cells?

We are using U2OS cells, because they do not have BRCA1 mutations and because they are standard cells for the investigation of the DNA damage response, DNA repair, PARP1 recruitment kinetics and so forth and have been used in multiple studies in the literature. We also used human breast cancer cells and murine fibroblasts to confirm our results.

- Fig. EV6A, as control, basal mRNA levels of NUAK2, PUMA and PTX3 in untreated TSG101 knockout cells should be shown to confirm that expression of these genes is induced by irradiation and modulated by TSG101 deficiency.

These data are included in **Figure EV6A**.

- Fig. 6J - please indicate clearly that the presented image was assembled from two separate culture plates.

We have now indicated in the legend that the image is assembled from two separate culture plates.

Omissions/unclarities in figure legends
- Fig. 1A, how RLU were calculated for siRNA targeting a given gene (is it a value for a single siRNA, averaged values for all if more than one were used? another method?)

We have included a detailed description in the Materials and Methods section. The value used is for a single siRNA.

- Fig. EV1D - which hits from the primary screen were selected for the analysis: positive, negative or both?

We selected 320 positive hits for the rescreen with TNFα. **Table EV4** in the original manuscript (**now Dataset EV3**) contains the gene names of these 320 candidates.

- Fig. 2C - the values are "relative', what is 1 then? An expression of a given gene in non-irradiated cells? No information on statistical analysis.

We normalized the expression to control, which was set to 1. We have now included the details of the statistical analysis in the legends.

- Fig. EV2A - the authors should present p-ATM and p-65 status upon irradiation combined with TSG101 deficiency (to compare with Fig. 2B).

For this experiment, we are analyzing cells eight hours post irradiation to detect the luciferase signal. At this time point p65 phosphorylation as well as ATM autophosphorylation return to

basal level (Kolesnichenko et al. 2021, and references therein). This figure was used to demonstrate the knockdown efficiencies of TSG101 and ATM siRNAs.

- Fig. EV2B - no y-axis values given, how to interpret relative NFKBIA expression scored as almost 1, does etoposide induce expression of NFKBIA at all?

We apologize for this mistake and we have now added the y axis values in this panel (now **Figure EV2C**) for CXCL10. Furthermore, the normalization of *CXCL10* and *NFKBIA* gene expression data of TSG101 silenced cells was done relative to etoposide treated, non-targeting siRNA transfected control cells.

- Fig. 3C and D - TSG101 blots look the same, this should be stated in the legend

In **old Figures 3C and D** higher and lower molecular weight regions of the gel were shown. We have now replaced these panels by a **new Figure 3C**.

- Fig. 6B - what are mock conditions? No error bars, no information on the number of experiments for statistical analysis.

The mock condition is non-targeting siRNA transfected untreated cells. We have changed this panel and include a dose-response survival analysis of etoposide treated cells in the absence or presence of TSG101.

- Fig. 6D-G - is TSG101 depletion by siRNA still present after 7 days post-transfection? Immunoblotting control should be provided.

The transfection was performed 3 days prior to analysis. Therefore, cells at time point 7D were irradiated 7 days before analysis and transfected with siRNA 3 days before analysis. This methodology allowed us to perform processing and analysis of all samples at the same time points, thereby eliminating errors stemming from differential fixation. We have confirmed before that siRNA knockdown persists for 3 days (Kolesnichenko et al 2021).

- Fig. EV6D - wrong description of colors used for graph bars

We have corrected this mistake.

- In many panels: what is mock control - etoposide-treated/irradiated cells with or without control non-targeting siRNA transfection? It should be clearly stated in each figure legend.

Mock refers to transfection with non-targeting siRNA. We have specified this in the respective legends.

- In many panels: no explanation of abbreviations: e.g. UT/IR, 10H PAR, EV

UT: Untreated, IR: Irradiation, 10H PAR: PAR binding antibody, EV: Empty vector. We have now added this information to the respective legends and apologize for this omission.

- In many panels: no information on statistics used and error bars presented on the graphs

All information on statistics are provided and the information regarding type of statistical analysis, number of replicates, and standard deviation remains in legends, as was the case in the original manuscript

- No or misleading information on the exact n number, e.g. in Fig. 2A "the data represent three biological and five technical replicates" so how was the n number calculated, 3 or 5?

The n number was 3 in these cases.

- Data of qRT-PCR should be averaged from several independent experiments, instead of showing a representative experiment like in Fig. 2C. For other qRT-PCR panels (Fig. 3F, 6C) there is no information on reproducibility at all.

We apologize, this was stated incorrectly, the number refers to the average.

Omissions in Methods
- No catalogue numbers/manufacturers provided for crucial reagents (like siRNA, drugs, NAD assay kit)
- No information on non-targeting siRNA sequence(s)
- No statistical analysis information provided for non-fluorescent tests (like in EV2)
- 'Key Resources Table for the secondary antibodies and the fluorophores' not provided

We apologize for this omission and we have now included this information in the Materials and Methods section and Expanded View Table 9 (previous Expanded View Table 8).

Altogether, we sincerely thank referee 1 for the extraordinary insight and oversight and extensive invested work time, which has allowed us to make a number of essential improvements and corrections of our work! We hope that our responses have completely ruled out the principal concerns and that the referee can now share our excitement about the work and the conclusions regarding the dissection of the pathway and the essential role of TSG101 in PARylation.

Referee #2:

Tufan et al. performed a genome-wide siRNA screen to identify regulators of the NF-κB pathway, based on a luciferase reporter readout. While the initial screen was done with the DNA damaging agent etoposide, a counter screen was performed for selected hits with the pro-inflammatory cytokine TNFα. This allowed for identification of NF-κB activators that are specific to the DNA damage-induced pathway. The screens identified several known and expected NF-κB regulators, but also some new ones, including TSG101. The authors validate that TSG101 is involved in DNA damage-induced NF-κB activation and NF-κB target gene expression and reveal that TSG101 interacts with PARP1 and is required for PARP1-mediated PAR formation. In the absence of TSG101, impaired PAR formation is associated with elevated PARP1 trapping on chromatin and reduced formation of a PARP1-IKKγ signalosome needed for IKK-NF-κB activation. Furthermore, absence of TSG101 phenocopies the effect of PARP inhibition, causing synthetic lethality in BRCA-deficient cells. Overall, this is an original, well-written study and the identification of TSG101 as novel PARP1 activator being required for PARP1-IKKγ signalosome formation and NF-κB activation after DNA damage is interesting, even though the mechanism how TSG101 activates PARP1 remains unclear. There are several additional points, however, which are not fully convincing, and which would require additional experimental validation:

We thank the referee for recognizing our work as original, well-written and the results as interesting. We have, as suggested, experimentally addressed the points raised by referee.

Main points:

1) Most experiments were done with a single siRNA against TSG101. To rule out off-target effects, at least a second independent siRNA against TSG101 should be included to confirm key results, such as the NF-κB activation and NF-κB target gene induction in Fig. 2, the PARP1 activation and PAR formation in Fig. 3, the PARP1 retention on damaged chromatin in Fig. 5, and the DNA repair defects and apoptosis in Fig. 6.

We thank the referee for this very useful suggestion and we have now used two additional siRNAs to confirm our findings. These data are now present as **new Figure 2D** (EMSA), **new Figure EV2B** (p65 phosphorylation) and **new Figure EV3A** (PARylation). These new experiments with additional siRNAs against TSG101 fully confirm our model that TSG101 is required for PARylation and PAR dependent NF-κB activation. We also refer to our response to referee 1. In the original manuscript, we have used three different siRNAs against TSG101 in the primary screen and further three different TSG101 siRNAs in the counter screen. We also used different sgRNAs against TSG101.

2) It would be important to show that the severe defect in PAR formation after TSG101 knockdown (Fig. 3) can be rescued by expression of siRNA-resistant TSG101, and to test if it can be rescued by TSG101 fragments that can/cannot interact with PARP1.

As suggested, we performed a quantified rescue experiment using a TSG101 expressing plasmid resistant to siRNA against TSG101 (**new Figure EV3B**). This experiment shows that ectopic overexpression of wildtype TSG101 rescues PARylation in TSG101 depleted cells. The rescuing mutation is shown in **Figure R3**. The result confirms that the observations regarding PARylation are due to TSG101 depletion and not to off-target effects. Since the result of the *in vitro* PARylation experiment using purified proteins can only be explained by a direct interaction, we did not further pursue rescue experiments with other PARP1-interacting and non-interacting TSG101 mutants.

3) The authors write that "TSG101 is required for the induced expression of selected NF-κB target genes (CCL2, CXCL10, ICAM1, CXCL8, NFKBIA)". How was this selection done? Are other DNA damage-induced NF-κB target genes not dependent on TSG101? RNA-seq experiments could clarify this point.

By using RNA-seq, we have previously identified *bona fide* NF-κB targets after DNA damage induction in U2OS cells in a systematic manner (Kolesnichenko et al., 2021) and selected the candidates tested here, which were also known from diverse other studies and are classical direct NF-κB targets. We have not performed RNAseq analysis for this project, however, we validated the selection of NF-κB targets using RT-qPCR. We would exclude that other DNA damage-induced NF-κB target genes are independent of TSG101, since TSG101 is essential for NF-κB activation, as we have shown for the levels IKK activation/p65 phosphorylation, DNA binding and transcriptional activity (**Figure 2**). This does not exclude the possibility that additional DNA damage-induced transcription factors (AP1, for instance) could lead to a certain level of activation of some genes even in the absence of NF-κB.

4) PARP1 trapping on chromatin can be analyzed biochemically (PMID: 23118055) or by IF after pre-extraction (PMID: 32755579, PMID: 33462394). At least one of these approaches should be used to complement the PARP1 recruitment kinetics shown in Fig. 5.

We believe that the live cell imaging technique gives the best-resolved analysis and the recruitment behavior and kinetics of PARP1 in presence and absence of TSG101 and the data can be best compared to reports in Mortusewicz et al., 2007, Stilmann et al. 2009 and other reports in the literature where PARP1 was analysed using the same method. We agree that a detailed further biochemical investigation of PARP1 chromatin trapping might provide interesting insights, but would require a comprehensive parallel biochemical analysis of TSG101 and is beyond the scope of this article.

5) In the screens, PARG is identified as negative regulator of IKK-NF-κB signaling. Given that TSG101 knockdown impairs IKK-NF-κB signaling by reducing PAR levels, it would be very interesting to test whether PARG inhibition rescues PAR formation and IKK-NF-κB activation in TSG101-deficient cells.

The fact that PARG was identified as a negative regulator underscores the quality of the genome-wide screen. Our data clearly reveal that TSG101 promotes PAR formation through interaction with PARP1. Inhibition or silencing of PARG may cause elevation of some residual PAR levels. However, the biological significance of this regulation may be unclear and require more analysis, addressing the type of PAR polymers, involvement of other PARP family members, etc. Again, this would not remain within the focus of the manuscript. In addition to PARG with its predicted function in the pathway, our screen revealed scores of additional interesting new regulators, which could not yet be further investigated in the frame of this one article.

6) For the experiments shown in Fig. 6E and 6G, are similar effects observed when Olaparib is used instead of TSG101 knockdown? And would the combination of TSG101 and Olaparib be epistatic or rather additive/synergistic? In terms of potential clinical relevance, combining PARP inhibition with TSG101 loss could also be interesting in the context of the experiments shown in Fig. 6H and 6J (6J should be quantified).

We used olaparib and TSG101 silencing under various conditions in parallel and in combination (**Figure 3A**, **new Figure 4A and B**, **Figure 5C and D**, and **new Figure 6H** (below) and in each case the effect was very similar and explainable by abrogated PARylation. We thank the referee for the suggestion to test combined TSG101 silencing and PARP inhibition. As recommended, we have performed the experiment and the data are included as **new Figure 6H**. In fact, the combination of TSG101 silencing and olaparib administration does not significantly further enhance cell killing of BRCA1/2 defective breast cancer cells. Therefore, PARP inhibition and loss of TSG101 function are epistatic. The clinical relevance of this findings is now addressed in the discussion.

Additional points:

1) The specificity of the PLA assays should be validated by single knockdowns of the two proteins, TSG101 and PARP1.

We thank the referee for raising this point and we now included these important negative controls in the **new Figures 4A, B and EV4B**. We used knockdown of BRCA1 with different

siRNAs or the use only of single antibodies against PARP1 or TSG101, which in each case eliminated the specific signals.

2) In Fig. 4E, the control condition seems to be missing.

Because we focused on the relative effect of the two transfected TSG101 mutants, the untransfected samples were not shown in this figure. However, the values can be compared to NAD^+/NADH ratios shown in EV3D for overexpressed or silenced TSG101.

3) It would be informative to show PARP1 levels for the experiments in Fig. 3B-D.

Vinculin is used as a loading control. PARP1 (**see Figure 3A**) is an abundant nuclear protein and its expression is not affected by siTSG101. Therefore, we did not include a further blot in **new Figures 3B and C**.

4) The results shown in Fig. 4F are not very clear. It could be replaced by a better replicate result or repeated.

We have now replaced the figure with a new *in vitro* PARylation experiment as a **new Figure 4F**. In addition to purified TSG101, we also used equivalent protein amounts of BSA or RNF113A as negative controls. The latter demonstrate the specificity of TSG101 in inducing PARylation. RNF113A is similar in size to TSG101. The data demonstrate that PARylation, *i.e.* the enzymatic activity of PARP1, is strongly enhanced by TSG101.

5) The results shown in Fig. 5G should be quantified as was done in Fig. 5C,D.

We believe that the result in **Figure 5G** shows in a qualitatively completely convincing and impressive manner that PARP1-GFP is rapidly recruited into the laser micro-irradiation sites within a second and that the signal is completely lost after 15 minutes. There is no change upon silencing of UBAP1.

6) The specificity of the TSG101 IF signal should be confirmed by TSG101 knockdown.

We have now included a new figure showing that the TSG101 IF signal is lost upon knockdown of TSG101 (**new Figure EV5A**).

7) Most PARP inhibitors, including Olaparib, target PARP1, but also inhibit PARP2 (and perhaps also other PARP family members). It would therefore be better to refer to these compounds as "PARP inhibitor/s" rather than "PARP1 inhibitor/s".

We have rephrased this correspondingly throughout the manuscript.

8) At the end of the introduction, the authors write "unrepaired DNA foci accumulate". Perhaps better: "γH2AX foci indicating unrepaired DNA damage accumulate".

We thank the referee for this suggestion. We have incorporated the improved phrase in the text.

9) In the discussion, the list of PARP1 interaction partners that regulate PARP1 activity and trapping could easily be extended to include PARP2/3, PARG, TRIP12, RNF4, p97.

We have now extended the discussion section to include the proteins listed above.

10) In the sentence "..., excluding that any remote mechanisms ..." please check grammar.

We have now corrected this sentence in the revised manuscript.

Finally, we want to thank referee 2 for the time invested, for careful reading and the excellent constructive criticisms and the very helpful suggestions which allowed us to improve the manuscript. As the referee can see, all the alterations and new experiments supported and strengthened our previous conclusions. We hope, that the referee is satisfied with the improvements and can share our excitement about the study!

Referee #3:

EMBOJ-2021-110372 by Tufan et al.
"TSG101 Associates with PARP1 and is Essential for PARylation and DNA Damage-induced NF-κB Activation"

Summary

In Tufan et al., the authors investigated targets in the DNA-damage-dependent NFkB activation pathway. They found the protein TSG101 in their screen that is an activator of NFkB signaling. Moreover, TSG101 interacts with and activates PARP-1-mediated ADP-ribosylation in order to stimulate NFkB activity. While this protein was previously shown to be involved the endosomal sorting complex, this study describes a new role for it. From their results, the authors conclude that TSG101 might serve as "a therapeutic target to achieve synthetic lethality in cancer treatment."

Review

This paper addresses an important aspect of biology related to cancer biology with potential therapeutic implications. The new role for TSG101 is interesting and may have broader implications. Nonetheless, the authors still need to address a few issues, including additional controls.

Strengths: This work discovers a previously unknown function of TSG101 in the DNA-damage response using detailed biochemical assays. This work has potential therapeutic implications.

We thank the referee for this positive assessment of our study.

Weaknesses: Aspects of the work are incomplete and a number of experiments require additional controls.

We appreciate this positive general feedback and hope that the changes described below led to a further improvement of the manuscript.

Additional Comments:

1. In the screen shown in Figure 1, the authors should show where PARP-1 itself is enriched. This is a critical control in view of their subsequent experiments and the overall hypothesis of the study.

PARP1 may affect gene expression through multiple mechanisms, often involving the poly(ADP-ribosyl)ation of chromatin proteins. This may positively or negatively regulate the transcriptional outcome. Previous studies proposed that PARP1 may positively affect transcriptional activity of activity of cytokine-induced NF-κB via interaction of co-activators (Hassa et al., J. Biol. Chem. 2003, PMID: 12960163), but it may also negatively control the activity of transcription factors, as was recently for MyoD (Matteini et al. Sci Rep, 2020, PMC7493885). In any case, a negative role of PARP1 in etoposide-induced reporter gene expression has not been investigated, but may take place at different levels.

Nevertheless, our best explanation is the potential redundancy of PARP1 and PARP2, the two PARPs that are responsive to DNA damage. Indeed, we could show that PARP2 also associates with TSG101 (**new Figure R4**).

2. For Figure 2B, the authors should show the level of total p65 along with the changes in p65 phosphorylation.

We have now included the requested analysis of p65 in the **new Figure EV2B** and it is evident that the changes in pp65 are not due to alterations in p65 levels. In addition to this, it can be seen in **Figure 2B** and **new Figure EV2B** that an equal low level of basal pp65 (or cross-reaction with p65) is seen in all lanes, except for lane 2, where pp65 is strongly induced.

3. Figure 3A should include the PAR levels in order to assess the effectivity of PARP-1 inhibition by Olaparib.

We thank the referee for this very good suggestion and now include this analysis showing the efficacy of PARP inhibition by olaparib in **new Figure 3A**.

4. A major conclusion the authors draw from Figure 3 is that TSG101 is required for PARP-1 interaction with ATM, IKK, and other components of the NFkB signalosome. However, no data has been shown to support this.

We agree with the referee that we did not formally show that these interactions are lost upon TSG101 depletion and did not claim this in the manuscript. However, we previously showed that TSG101 interacts with IKKγ in a DNA damage-induced manner, as expected for a PAR/PARP1 dependent process (Mikuda et al., 2018, cited in the manuscript). We already demonstrated in previous work in great experimental detail that DNA damage triggers an interaction of ATM, PARP1, IKKγ, and PIASy that is PARP1 and PAR-dependent, involves PAR-binding motifs in PIASy and presumably in ATM and is required for activation of IKK and NF-κB only following DNA damage-induction (Stilmann et al., 2009). Since TSG101 depletion completely ablates PAR levels, we propose that the link between TSG101 and DNA damage-induced NF-κB activation occurs through the formation of this short-lived signalosome.

5. In Figure 4A, the PLA signal appears to be stronger in the Olaparib treated condition. If this holds true, it goes against the later conclusions. Quantifying the PLA data is therefore essential to support the author's claims.

We thank the referee for this suggestion. We performed additional PLA experiments and according to quantitation of biological replicates from these experiments, as requested, we show that indeed the PLA signals are more abundant in olaparib treated cells, and as well in Etoposide treated cells (**new Figures 4A, 4B and EV4B**). This, however, does not interfere with our model. This interesting observation requires additional research and will be addressed in future studies.

6. The data in Figure 4E seems counter intuitive. If I understand this correctly, increased binding of PARP-1 to the CC+SB domains should increase the PAR levels and, subsequently, lead to more NAD+ being consumed. However, the results shown here are in the opposite direction. Can the authors clarify this?

From this result the NADH consumption can be observed by decreased NADH levels in the case of irradiated PAR-producing CC-SB-transfected cells, compared to ΔCC-transfected cells,

which produce less PAR (**new Figure EV4D**). As a result of depleted NADH in CC-SB cells, the NAD+/NADH ratio is expectedly higher than in ΔCC-transfected cells (**Figure 4E**). It is also evident in **Figure EV3D** that a decreased NAD+/NADH level is observed, when consumption of NADH by PARP1 is blocked through either pharmacological inhibition by olaparib or TSG101 knockdown.

7. Is the retention of PARP-1 observed with Olaparib due to PARP trapping? Furthermore, does TSG101 depletion cause the same phenomenon?

The pretreatment of irradiated cells with olaparib leads to PARP trapping and this was previously shown by others in a number of publications (e.g. Murai et al, Cancer Res, 2020, PMID: 23118055). That olaparib and loss of TSG101 cause the same phenomenon is also evident from **Figures 5C and D**, where we explicitly showed as a main result that olaparib and TSG101 depletion result in retention of PARP1. A further new experiment now revealed that TSG101 depletion and olaparib function in an epistatic manner, *i.e.* when combined these treatments have no further significant effect on cell killing than the single treatments (**new Figure 6H**). This implies that their common effect is the lack of PARylation.

8. The in the sentence "We selected the 1.000 top hits as candidate activators of the pathway and 100 further hits," should 1.000 be written as 1000?

We thank the referee and have corrected this.

We thank referee 3 for the helpful and constructive criticism and for the time spent for going through our data. We hope that our responses and alterations, which have strengthened the manuscript, have satisfied the referees requests.

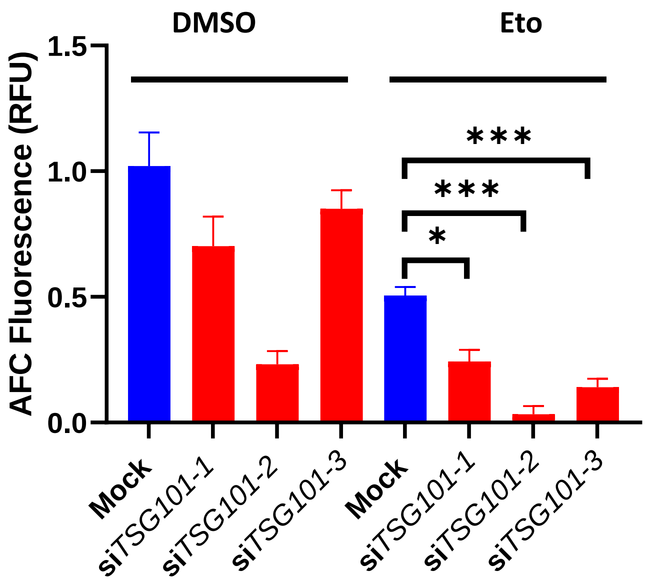

Figure R1. U2-OS cells were transfected with non-targeting (mock) or three different *TSG101*-targeting siRNAs. Cells were treated with etoposide (25 µM) for 96 hours. Relative number of viable cells in culture was measured using GF-AFC and normalized to non-targeting siRNA-transfected DMSO-treated cells. Data represent three biologically independent experiments and the conditions were compared with an ordinary one-way ANOVA (ns, $p > 0.05$; *$p < 0.05$; **$p < 0.01$, ***$p < 0.001$; ****$p < 0.0001$).

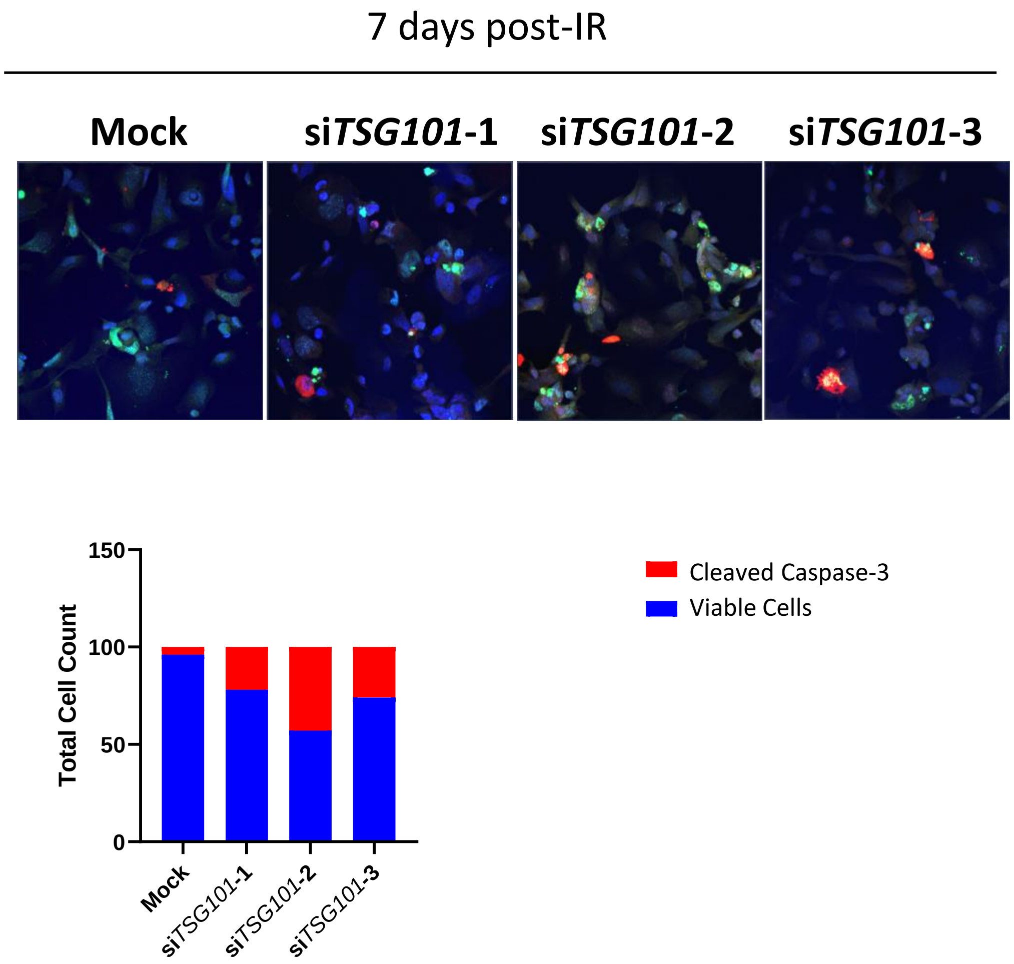

Figure R2. Cells were transfected with non-targeting or three different *TSG101*-targeting siRNAs. Immunofluorescence staining was performed with the respective antibodies (red: cleaved caspase 3; green: γH2AX). Nuclei were stained with DAPI (blue). The image is a representative of 3 biologically independent experiments.

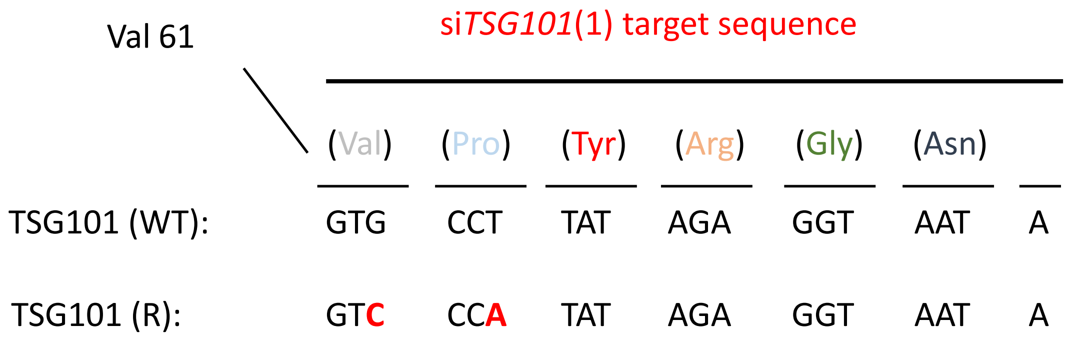

Figure R3. Generation of an siRNA resistant TSG101 construct. Alternative nucleotides were introduced by sequential side-directed mutagenesis into the codons of Val 61 and Pro 62 as indicated in red.

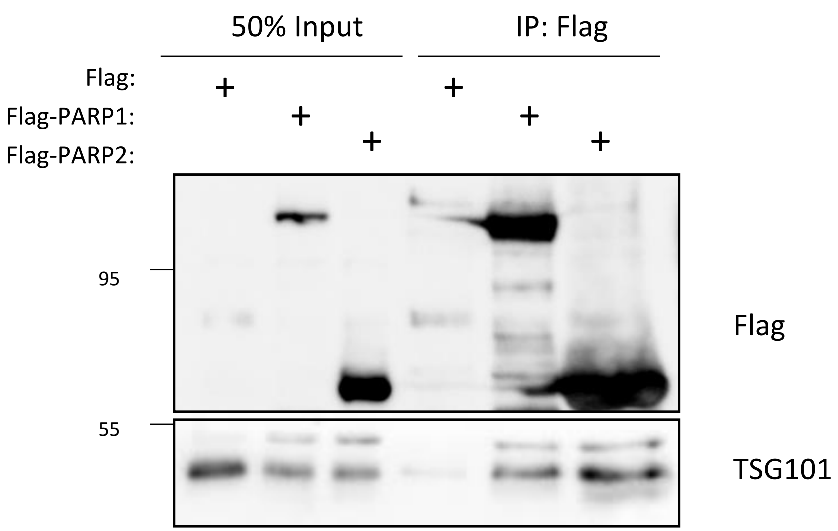

Figure R4. U2-OS cells were transfected with empty Flag vector, Flag-PARP1 or Flag-PARP2 plasmids. Immunoprecipitation of Flag was performed from whole-cell extracts and subsequently analyzed by immunoblotting.

Thank you for submitting a new version of your manuscript on TSG101 and PARylation for our consideration. It has now been re-reviewed by the three original referees, and I am happy to say that all of them consider the study significantly improved and now in principle suitable for publication. As you will see, they nevertheless retain a few specific concerns (including one unaddressed experimental issue), which I would kindly invite you to address in a final round of re-revision.

In addition, this final round of modification should also incorporate a few remaining editorial points:

Referee #1:

The manuscript by Tufan et al has now undergone a major revision. The authors responded to all previously raised concerns and for most of them provided additional data and controls as requested. The study has been significantly improved and its conclusions seem to be strengthened enough to merit publication.
However, it is still recommended to carefully review the text and the figures, as not all the corrections listed as done in the rebuttal have been actually introduced in the manuscript. For example, the authors claim that they now deleted the word "direct" in the manuscript in context of the TSG101-PARP1 interaction (point 7, referee 1) but the abstract still reads "TSG101 directly binds to PARP1". The spelling "1.000" has not been corrected (point 8 of referee 3). There seems to be a mistake in the description of lanes of new Fig. 3A - the last two nuclear fractions are marked as if treated in the same way which is inconsistent with the corresponding cytoplasmic fractions.

Referee #2:

The authors addressed many of my initial concerns and the conclusions have been strengthened by additional experimental data. In particular, the use of a 2nd and 3rd siRNA against TSG101 corroborate initial findings and previously missing specificity controls improved the manuscript.

Remaining major point(s):

1) As before (previous main point 1), I recommend using the 2nd and 3rd siRNA against TSG101 also for the PARP1 trapping experiments shown in Figure 5 (Figure 5 B-D) and for the DNA repair experiments shown in Figure 6 (Figure 6 D, F, G). This would lend robustness to the PARP1-related DNA repair phenotypes, including the synthetic lethality in BRCA-mutated MDA-MB-436 cells, and rule out off-target effects at the level of these important readouts.

Minor points:

1) Figure 4B lacks a y-axis label.
2) Figure 5E contains a white line that can probably be removed.
3) In Figure 5F it is unclear what the enlargement is supposed to illustrate and whether it is needed.

Referee #3:

The authors have addressed my concerns. The paper has been improved. This is an interesting topic and this paper adds important new observations to the literature.

Final rebuttal

Referee #1:

The manuscript by Tufan et al has now undergone a major revision. The authors responded to all previously raised concerns and for most of them provided additional data and controls as requested. The study has been significantly improved and its conclusions seem to be strengthened enough to merit publication.

We thank the referee for agreeing with our significant improvements and that the work now merits publication.

However, it is still recommended to carefully review the text and the figures, as not all the corrections listed as done in the rebuttal have been actually introduced in the manuscript. For example, the authors claim that they now deleted the word "direct" in the manuscript in context of the TSG101-PARP1 interaction (point 7, referee 1) but the abstract still reads "TSG101 directly binds to PARP1". The spelling "1.000" has not been corrected (point 8 of referee 3).

We apologize that we have overseen the omissions and have now corrected these points.

There seems to be a mistake in the description of lanes of new Fig. 3A - the last two nuclear fractions are marked as if treated in the same way which is inconsistent with the corresponding cytoplasmic fractions.

We have corrected new Fig. 3A now by adding '+' instead of '-'.

Referee #2:

The authors addressed many of my initial concerns and the conclusions have been strengthened by additional experimental data. In particular, the use of a 2nd and 3rd siRNA against TSG101 corroborate initial findings and previously missing specificity controls improved the manuscript.

We thank the referee for agreeing with the significant improvement of the manuscript.

Remaining major point(s):

As before (previous main point 1), I recommend using the 2nd and 3rd siRNA against TSG101 also for the PARP1 trapping experiments shown in Figure 5 (Figure 5 B-D) and for the DNA repair experiments shown in Figure 6 (Figure 6 D, F, G). This would lend robustness to the PARP1-related DNA repair phenotypes, including the synthetic lethality in BRCA-mutated MDA-MB-436 cells, and rule out off-target effects at the level of these important readouts.

As pointed out in our previous rebuttal letter, by using additional TSG101 siRNAs, we fully confirmed our previous observations regarding DNA damage-induced p65 phosphorylation (new Figure EV2B), NF-κB activation (assessed by EMSA) in new Figure 2D, irradiation-induced poly(ADP ribosyl)ation in new Figure EV3A, etoposide-induced cell death in (Figure for referees R1), as well as irradiation-induced caspase 3 cleavage (Figure for referees R2)". Since equivalent results were obtained with three different siRNAs against TSG101 in each of these experimentally different

settings, we have no doubt, off-target effects should not be a problem. Figures R1 and R2, which were embedded in the rebuttal letter PDF file, already contained the suggested experiments with additional siRNAs against TSG101 for Figure 6. Furthermore, usage of additional siRNAs in BRCA deficient cells were not specifically suggested by this referee (please see comment 1 of R2).

Minor points:

1) Figure 4B lacks a y-axis label.

We have now introduced a y-axis label ‘PLA dots per nucleus’.

2) Figure 5E contains a white line that can probably be removed.

This graphic error has been removed.

3) In Figure 5F it is unclear what the enlargement is supposed to illustrate and whether it is needed.

As suggested, the enlargement including the two arrows has been removed.

We thank the referee again for the careful inspection, which allowed us to fix these points.

<u>Referee #3:</u>

The authors have addressed my concerns. The paper has been improved. This is an interesting topic and this paper adds important new observations to the literature.

We thank the reviewer for the final support.

2nd Revision - Editorial Decision 25th Aug 2022

Thank you for submitting your final revised manuscript for our consideration. I have now checked your responses, and given the presence of the referee figures in the previous response letter (which I think should therefore be retained in the Review Process File), we shall be happy to accept the manuscript for The EMBO Journal now without further experiments.

EMBO Press Author Checklist

Corresponding Author Name: Claus Scheidereit
Journal Submitted to: EMBO J
Manuscript Number: EMBOJ-2022-111971

USEFUL LINKS FOR COMPLETING THIS FORM
The EMBO Journal - Author Guidelines
EMBO Reports - Author Guidelines
Molecular Systems Biology - Author Guidelines
EMBO Molecular Medicine - Author Guidelines

Reporting Checklist for Life Science Articles (updated January
This checklist is adapted from Materials Design Analysis Reporting (MDAR) Checklist for Authors. MDAR establishes a minimum set of requirements in transparent reporting in the life sciences (see Statement of Task: 10.31222/osf.io/9sm4x). Please follow the journal's guidelines in preparing your
Please note that a copy of this checklist will be published alongside your article.

Abridged guidelines for figures
1. Data
The data shown in figures should satisfy the following conditions:
- the data were obtained and processed according to the field's best practice and are presented to reflect the results of the experiments in an accurate and unbiased manner.
- ideally, figure panels should include only measurements that are directly comparable to each other and obtained with the same assay.
- plots include clearly labeled error bars for independent experiments and sample sizes. Unless justified, error bars should not be shown for technical
- if n<5, the individual data points from each experiment should be plotted. Any statistical test employed should be justified.
- Source Data should be included to report the data underlying figures according to the guidelines set out in the authorship guidelines on Data

2. Captions
Each figure caption should contain the following information, for each panel where they are relevant:
- a specification of the experimental system investigated (eg cell line, species name).
- the assay(s) and method(s) used to carry out the reported observations and measurements.
- an explicit mention of the biological and chemical entity(ies) that are being measured.
- an explicit mention of the biological and chemical entity(ies) that are altered/varied/perturbed in a controlled manner.
- the exact sample size (n) for each experimental group/condition, given as a number, not a range;
- a description of the sample collection allowing the reader to understand whether the samples represent technical or biological replicates (including how many animals, litters, cultures, etc.).
- a statement of how many times the experiment shown was independently replicated in the laboratory.
- definitions of statistical methods and measures:
  - common tests, such as t-test (please specify whether paired vs. unpaired), simple χ2 tests, Wilcoxon and Mann-Whitney tests, can be unambiguously identified by name only, but more complex techniques should be described in the methods section;
  - are tests one-sided or two-sided?
  - are there adjustments for multiple comparisons?
  - exact statistical test results, e.g., P values = x but not P values < x;
  - definition of 'center values' as median or average;
  - definition of error bars as s.d. or s.e.m.

Please complete ALL of the questions below.
Select "Not Applicable" only when the requested information is not relevant for your study.

Materials

Newly Created Materials	Information included in the manuscript?	In which section is the information available? (Reagents and Tools Table, Materials and Methods, Figures, Data Availability Section)
New materials and reagents need to be available; do any restrictions apply?	Not Applicable	

Antibodies	Information included in the manuscript?	In which section is the information available? (Reagents and Tools Table, Materials and Methods, Figures, Data Availability Section)
For **antibodies** provide the following information:<br>- Commercial antibodies: RRID (if possible) or supplier name, catalogue number and or/clone number<br>- Non-commercial: RRID or citation	Yes	Supplemental tables

DNA and RNA sequences	Information included in the manuscript?	In which section is the information available? (Reagents and Tools Table, Materials and Methods, Figures, Data Availability Section)
**Short novel DNA or RNA including primers, probes**: provide the sequences.	Yes	Supplemental tables

Cell materials	Information included in the manuscript?	In which section is the information available? (Reagents and Tools Table, Materials and Methods, Figures, Data Availability Section)
**Cell lines:** Provide species information, strain. Provide accession number in repository **OR** supplier name, catalog number, clone number, and/**OR** RRID.	Yes	Materials and methods and supplemental tables
**Primary cultures:** Provide species, strain, sex of origin, genetic modification status.	Not Applicable	
Report if the cell lines were recently **authenticated** (e.g., by STR profiling) and tested for mycoplasma contamination.	Yes	checked regularly for mycoplasma

Experimental animals	Information included in the manuscript?	In which section is the information available? (Reagents and Tools Table, Materials and Methods, Figures, Data Availability Section)
**Laboratory animals or Model organisms:** Provide species, strain, sex, age, genetic modification status. Provide accession number in repository **OR** supplier name, catalog number, clone number, **OR** RRID.	Not Applicable	
**Animal observed in or captured from the field:** Provide species, sex, and age where possible.	Not Applicable	
Please detail **housing and husbandry conditions**.	Not Applicable	

Plants and microbes	Information included in the manuscript?	In which section is the information available? (Reagents and Tools Table, Materials and Methods, Figures, Data Availability Section)
**Plants:** provide species and strain, ecotype and cultivar where relevant, unique accession number if available, and source (including location for collected wild specimens).	Not Applicable	
**Microbes:** provide species and strain, unique accession number if available, and source.	Not Applicable	

Human research participants	Information included in the manuscript?	In which section is the information available? (Reagents and Tools Table, Materials and Methods, Figures, Data Availability Section)
If collected and within the bounds of privacy constraints report on age, sex and gender or ethnicity for all study participants.	Not Applicable	

Core facilities	Information included in the manuscript?	In which section is the information available? (Reagents and Tools Table, Materials and Methods, Figures, Data Availability Section)
If your work benefited from core facilities, was their service mentioned in the acknowledgments section?	Yes	also as co authords on the manuscript

Design

Study protocol	Information included in the manuscript?	In which section is the information available? (Reagents and Tools Table, Materials and Methods, Figures, Data Availability Section)
If study protocol has been **pre-registered, provide DOI in the manuscript**. For clinical trials, provide the trial registration number **OR** cite DOI.	Not Applicable	
Report the **clinical trial registration number** (at ClinicalTrials.gov or equivalent), where applicable.	Not Applicable	

Laboratory protocol	Information included in the manuscript?	In which section is the information available? (Reagents and Tools Table, Materials and Methods, Figures, Data Availability Section)
Provide DOI OR other citation details if **external detailed step-by-step protocols** are available.	Not Applicable	

Experimental study design and statistics	Information included in the manuscript?	In which section is the information available? (Reagents and Tools Table, Materials and Methods, Figures, Data Availability Section)
Include a statement about **sample size** estimate even if no statistical methods were used.	Yes	at least three biological replicates were used
Were any steps taken to minimize the effects of subjective bias when allocating animals/samples to treatment (e.g. **randomization procedure**)? If yes, have they been described?	Not Applicable	
Include a statement about **blinding** even if no blinding was done.	Yes	blind counting was performed for several experiments requiring quantitation
Describe **inclusion/exclusion criteria** if samples or animals were excluded from the analysis. Were the criteria pre-established?<br><br>If sample or data points were omitted from analysis, report if this was due to attrition or intentional exclusion and provide justification.	Not Applicable	
For every figure, are **statistical tests** justified as appropriate? Do the data meet the assumptions of the tests (e.g., normal distribution)? Describe any methods used to assess it. Is there an estimate of variation within each group of data? Is the variance similar between the groups that are being statistically compared?	Yes	please see figure legends

Sample definition and in-laboratory replication	Information included in the manuscript?	In which section is the information available? (Reagents and Tools Table, Materials and Methods, Figures, Data Availability Section)
In the figure legends: state number of times the experiment was **replicated** in laboratory.	Yes	done in relevant figures
In the figure legends: define whether data describe **technical or biological replicates**.	Yes	was indicated

Ethics

Ethics	Information included in the manuscript?	In which section is the information available? (Reagents and Tools Table, Materials and Methods, Figures, Data Availability Section)
Studies involving **human participants**: State details of **authority granting ethics approval** (IRB or equivalent committee(s), provide reference number for approval.	Not Applicable	
Studies involving **human participants**: Include a statement confirming that **informed consent** was obtained from all subjects and that the experiments conformed to the principles set out in the WMA Declaration of Helsinki and the Department of Health and Human Services Belmont Report.	Not Applicable	
Studies involving **human participants:** For publication of **patient photos**, include a statement confirming that consent to publish was obtained.	Not Applicable	
Studies involving experimental **animals**: State details of **authority granting ethics approva**l (IRB or equivalent committee(s), provide reference number for approval. Include a statement of compliance with ethical regulations.	Not Applicable	
Studies involving **specimen and field samples:** State if relevant **permits** obtained, provide details of authority approving study; if none were required, explain why.	Not Applicable	

Dual Use Research of Concern (DURC)	Information included in the manuscript?	In which section is the information available? (Reagents and Tools Table, Materials and Methods, Figures, Data Availability Section)
Could your study fall under dual use research restrictions? Please check biosecurity documents and list of **select agents and toxins** (CDC): https://www.selectagents.gov/sat/list.htm	Not Applicable	
If you used a select agent, is the security level of the lab appropriate and reported in the manuscript?	Not Applicable	
If a study is subject to dual use research of concern regulations, is the name of the **authority granting approval and reference number** for the regulatory approval provided in the manuscript?	Not Applicable	

Reporting

The MDAR framework recommends adoption of discipline-specific guidelines, established and endorsed through community initiatives. Journals have their own policy about requiring specific guidelines and recommendations to complement MDAR.

Adherence to community standards	Information included in the manuscript?	In which section is the information available? (Reagents and Tools Table, Materials and Methods, Figures, Data Availability Section)
State if relevant guidelines or checklists (e.g., **ICMJE, MIBBI, ARRIVE, PRISMA**) have been followed or provided.	Not Applicable	
For **tumor marker prognostic studies,** we recommend that you follow the **REMARK** reporting guidelines (see link list at top right). See author guidelines, under 'Reporting Guidelines'. Please confirm you have followed these guidelines.	Not Applicable	
For **phase II and III randomized controlled trials**, please refer to the **CONSORT** flow diagram (see link list at top right) and submit the CONSORT checklist (see link list at top right) with your submission. See author guidelines, under 'Reporting Guidelines'. Please confirm you have submitted this list.	Not Applicable	

Data Availability

Data availability	Information included in the manuscript?	In which section is the information available? (Reagents and Tools Table, Materials and Methods, Figures, Data Availability Section)
Have **primary datasets** been deposited according to the journal's guidelines (see 'Data Deposition' section) and the respective accession numbers provided in the Data Availability Section?	Yes	will be done
Were **human clinical and genomic datasets** deposited in a public access-controlled repository in accordance to ethical obligations to the patients and to the applicable consent agreement?	Not Applicable	
Are **computational models** that are central and integral to a study available without restrictions in a machine-readable form? Were the relevant accession numbers or links provided?	Not Applicable	
If publicly available data were reused, provide the respective **data citations in the reference list**.	Not Applicable	
